# Study on ageing characteristics and evaluation methods of RTV silicone rubber in high humidity area

**DOI:** 10.1371/journal.pone.0251092

**Published:** 2021-06-04

**Authors:** Hao Yang, Ran Wen, Heng Zhao, Men Guo, Lu Zhang, Yu Chen

**Affiliations:** 1 School of Electronics and Information, Xi’an Polytechnic University, Xi’an, PR China; 2 State Key Laboratory of Electrical Insulation and Power Equipment, Xi’an Jiaotong University, Xi’an, PR China; 3 Electric Power Research Institute, State Grid Shaanxi Electric Power Company Xi’an, Xi’an, PR China; University of Naples Federico II, ITALY

## Abstract

Due to high humidity, the ageing of room temperature vulcanized silicone rubber (RTV) has been a serious problem in Southwestern China. In order to solve the problem of RTV life prediction, the aging classification method was established by analyzing the microtopography of RTV samples in this paper. Besides, the comprehensive analysis of RTV element content, partial element content ratio, and major chemical groups of RTV samples in each aging level were conducted. It is found that as the ageing level increases, the element contents of C, Si, O, Al change accordingly and the ratio of C:Si drops from 2.39 to 1.54, and absorption peaks of the chemical groups of Si-(CH_3_)_2_, Si-O-Si, Si-CH_3_ and C-H in CH_3_ decrease. This work can enrich the investigation of RTV, and may provide useful reference for performance evaluation and replacement of RTV in substations.

## 1 Introduction

In recent years, due to the excellent hydrophobicity and anti-pollution performance, RTV has been widely used in transformer substations [1–3]. However, under the influence of the outdoor environment, the service life of silicon rubber will be reduced [4]. And it is known that the stability of outdoor insulation is of great significance for the long-term reliable operation of power system [5].

During the service, silicon rubber endure strong electric field and can possibly be affected by many factors such as humidity, pollution and ultraviolet radiation [6]. After a long period of service, filler particles, holes and cracks may appear on RTV surface, which affects the insulation performance of RTV significantly. Due to the increase of particle clusters and holes on the surface of RTV, the contamination and moisture will accumulate, leading to the distortion of surface electric field distribution. Thus, corona discharge will occur frequently, resulting in further deterioration of the RTV surface.

Outdoor insulation performance of RTV is determined by its surface state, researchers have carried out a lot of work on polymers surface condition monitoring [7–9]. Based on the X-Day Diffraction analysis, Jia proposed that the SiO_2_ composition can be used to evaluate the surface state of RTV [10]. Xia established a relationship between thermal weight loss and RTV surface state [11]. Alok studied the performance changes of silicone rubber insulator under various stress aging, and reported the loss of aluminum trihydrate (ATH) filler in silicone rubber after aging [12]. Besides, Kumagai proposed an easy method to evaluate the state of the polymer surface by adopting the leakage current characteristics [13]. It can be concluded that most of the previous studies was carried out based on the laboratory samples, and the results can not be used to guide the field engineering unfortunately.

Besides, the relationship between physical and chemical properties and surface state can reveal the changing mechanism of RTV. Rahmat have done a lot of work to study the physical and chemical properties of polymer material. The hydrophobicity, mechanical test, leakage current test, Fourier infrared spectroscopy (FTIR) and scanning electron microscope (SEM) test are analyzed to study the influence of the multi-stress aging on high temperature vulcanized silicone rubber [14–16]. It is indicated that these analysis methods can be used to evaluate its performance and characterize the surface state.

It can be concluded that the study of RTV from on-site operating condition is relatively lacking. In this paper, the RTV samples from high humidity area are studied. Based on the microtopography of RTV surface, a new classification method is proposed. Moreover, the relationships between physical and chemical properties and surface state are established by adopting a variety of detecting methods.

## 2 Experimental samples and platforms

### 2.1. Sample information

In this paper, the RTV samples in the Yibin ±800kV UHV DC converter station are studied. Yibin ±800kV UHV DC converter station is owned by the State Grid Corporation of China, and we have got the authority to test the RTV coatings of the post insulators in this converter station. The converter station is located in Shuanglong Town, Yibin City, China, 104°35′ east longitude and 28°45′ north latitude. The annual average temperature is only 18°C, the average annual sunshine hours are 976 hours, the annual average air humidity is as high as 80%, and the climate type is mid-subtropical humid monsoon climate.

**Fig 1:**
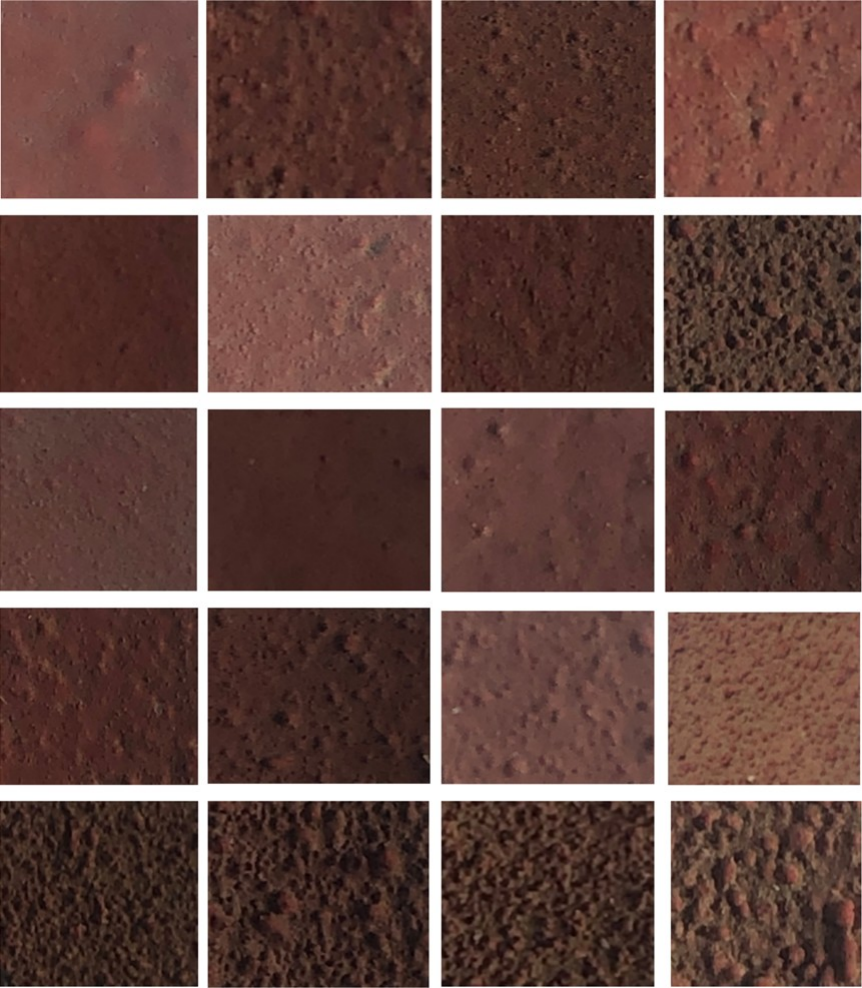
20 typical pieces of RTV samples.

All the samples have been in service for more than 7 years, and they are from high-voltage post insulator and low-voltage reactor post insulators respectively. 20 typical pieces of RTV samples are shown in Fig 1. It can be seen that these samples have filler particles, cracks and holes roughly.

**Fig 2:**
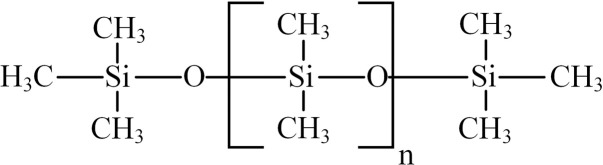
Molecular structure of polydimethylsiloxane (PDMS).

The main molecular structure of RTV used in Yinbin converter station is polydimethylsiloxane (PDMS) [17]. The molecular structure is shown in Fig 2. The structure of PDMS molecule is characterized by a helical, curved Si-O-Si long chain in the middle, and a layer of externally oriented methyl groups (-CH_3_) wrapping on the outside of the long chain.

### 2.2. Experiment platform

**Fig 3:**
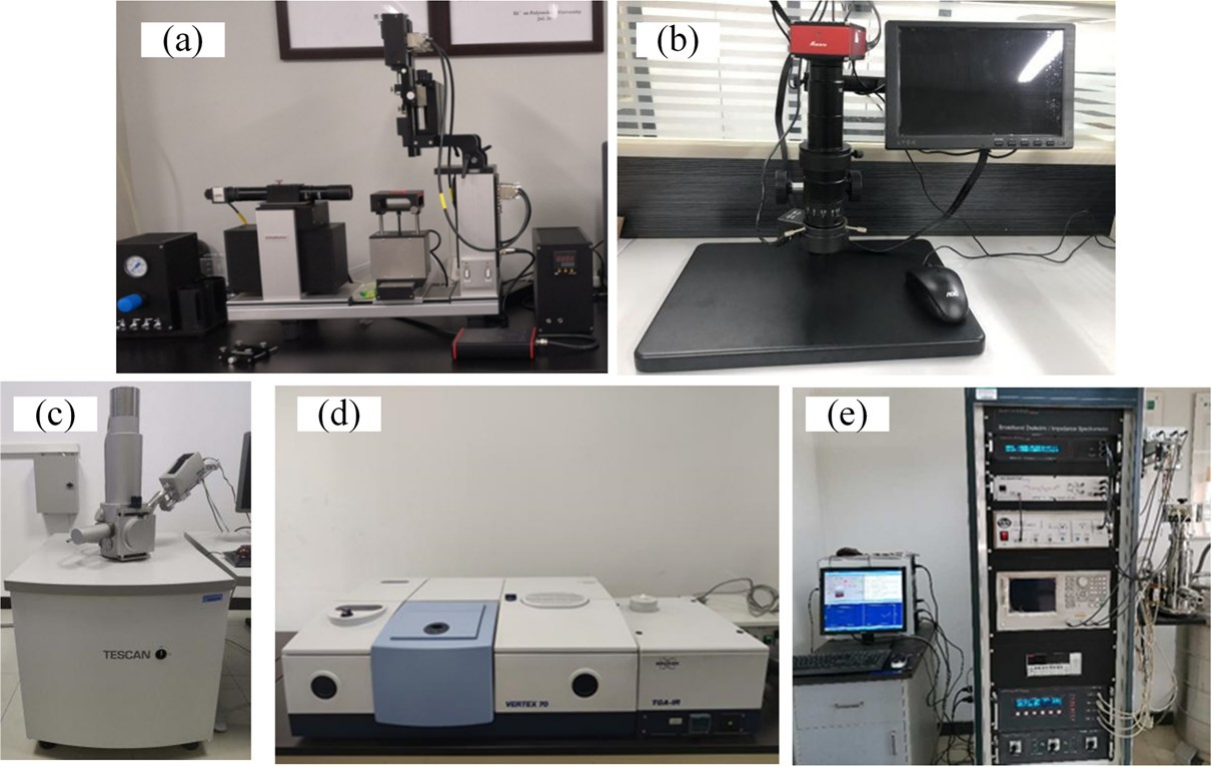
Experimental test instruments. (a) The OCA40Micro static contact angle measuring instrument; (b) The 10A series video microscope. (c) SEM-EDS. (d) The Vertex70 infrared spectrometer. (e) Broadband dielectric spectroscopy impedance meter.

Six tests were performed for the RTV samples, including hydrophobicity, surface morphology, surface microtopography, element content, chemical structure and dielectric properties. All the tests were conducted in the condition monitoring of power transmission and transformation equipment research center of Shaanxi Province, and the test instruments are shown in Fig 3.

#### (1) Hydrophobicity test

The OCA40Micro static contact angle measuring instrument produced by German Dataphysics Company is used to test the static contact angle of the RTV surface.

#### (2) Surface morphology

A video microscope produced by Nanjing Nanpai Technology Co., Ltd. is used to analyze the particle size of the RTV surface.

#### (3) Surface microtopography

The VEGA 3 SBH scanning electron microscope (SEM) produced by Czech TESCAN is used to characterize the RTV surface microscopic morphology, and the magnification is set to 1000 times.

#### (4) Element analysis

The Octtance Prime energy dispersive spectrum analyzer (EDS) produced by EDAX, USA is used to analyze the surface chemical elements of RTV.

#### (5) Chemical structure analysis

The Vertex70 infrared spectrometer (FTIR) produced by German Bruker is used to analyze the surface chemical structure of the RTV sample, and the analysis wavelength is set to 4000cm^-1^~400cm^-1^.

#### (6) Dielectric properties

Broadband dielectric spectroscopy impedance meter produced by Novocontrol, Germany is used to analyze the dielectric constant, dielectric loss and conductivity of RTV sample at 20°C and 50Hz.

## 3 Surface characteristic test

### 3.1. Hydrophobicity analysis

**Fig 4:**
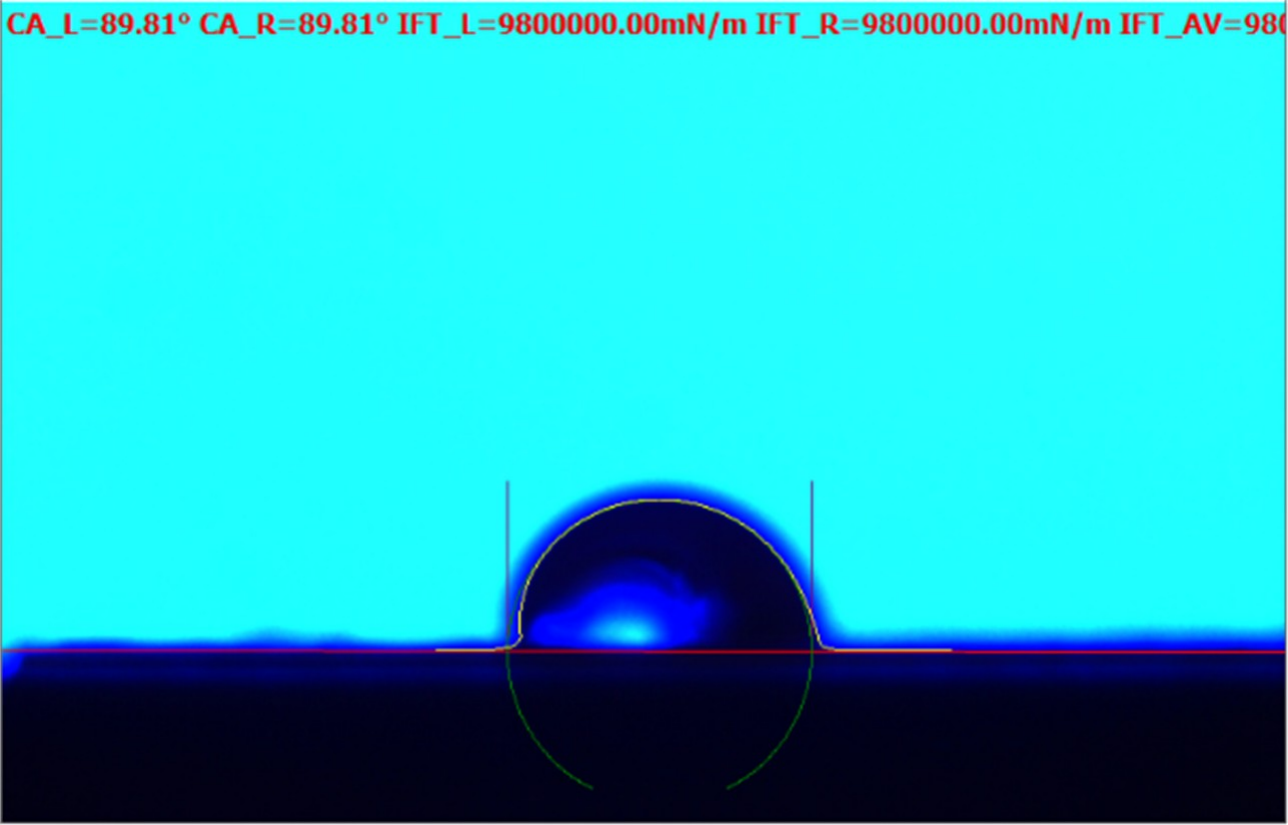
Typical image of the static contact angle test.

The static contact angle method is employed the test the RTV samples [18]. And the typical image of the static contact angle test is shown in Fig 4. The static contact angle of the RTV samples are shown in Table 1. It can be seen that the static contact angle value has big difference even though the RTV samples are removed from the same post insulator. Thus, it is not possible to only use the hydrophobicity parameter to evaluate the surface condition of the on-site RTV. Due to the complexity of outdoor influencing factors, the static contact angle method cannot accurately evaluate the surface hydrophobicity of RTV.

**Table 1:** Typical static contact angle of RTV.

Sampling site (No.)	High-voltage post insulator			Low-voltage reactor post insulator		
	1	2	3	1	2	3
**Static contact angle (°)**	87.18	127.1	144.5	109.27	127.10	139.65

### 3.2 Surface particle size test

#### 1) Surface microtopography

**Fig 5:**
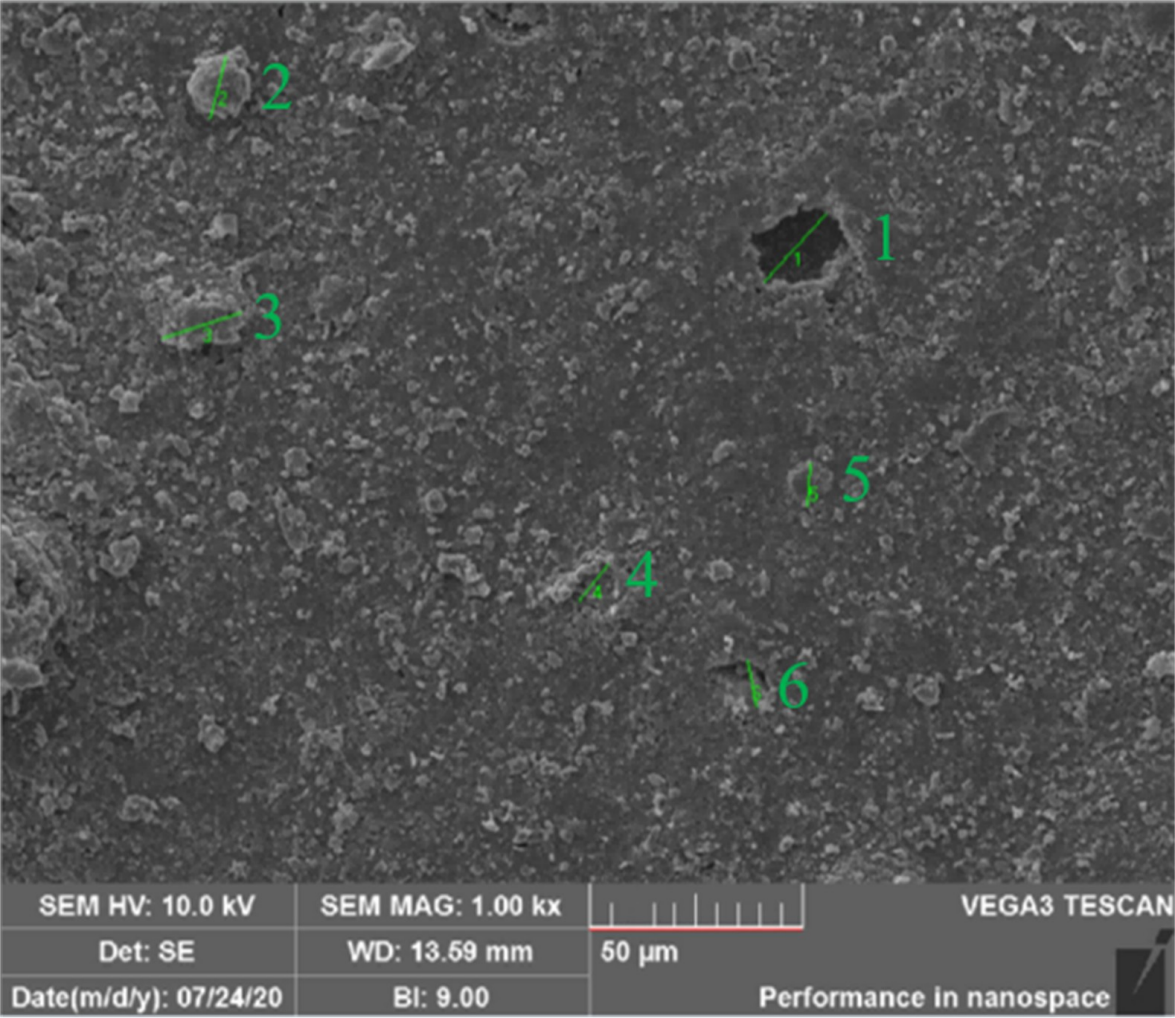
SEM image of RTV sample.

In this section, SEM is used to get the surface micromorphology of RTV samples. The typical SEM image of one single RTV sample is shown in Fig 5. The diameters of obvious particles and holes are analyzed, and the average diameter of these particles are defined to represent the surface roughness of the RTV sample. All the results of SEM analysis are also shown in Table 2.

**Table 2:** Surface roughness of the 20 pieces of RTV.

Test	No.	Average diameter (μm)	No.	Average diameter (μm)	No.	Average diameter (μm)	No.	Average diameter (μm)
**Surface microtopography**	**1**	13.48	**6**	17.38	**11**	25.86	**16**	108.03
	**2**	14.54	**7**	19.54	**12**	40.91	**17**	116.20
	**3**	16.68	**8**	20.19	**13**	41.77	**18**	128.83
	**4**	17.10	**9**	20.32	**14**	42.08	**19**	157.61
	**5**	17.38	**10**	25.27	**15**	42.76	**20**	170.30

The RTV samples with surface roughness of 10–40μm, 40–100μm and over 100μm are primarily designated as ageing level 1, ageing level 2 and ageing level 3, respectively.

**Fig 6:**
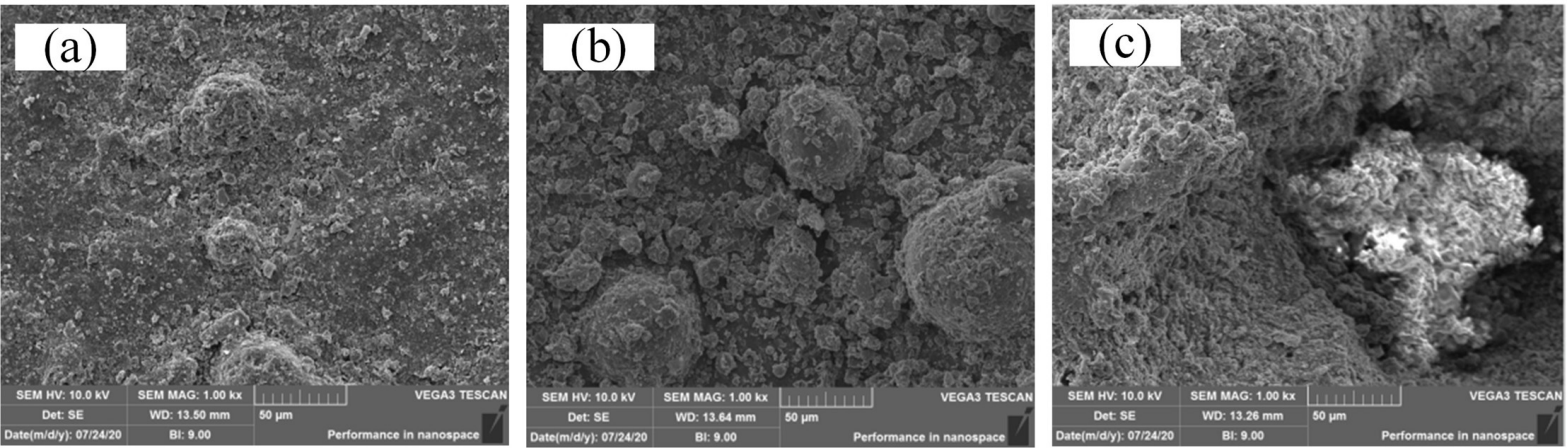
Three RTV samples with relatively different surface roughness. (a) RTV samples with surface roughness of 10–40μm; (b) RTV samples with surface roughness of 40–100μm; (c) RTV samples with surface roughness of more than 100μm.

Level 1: The microscopic morphology of RTV samples in this level with a particle diameter of 10–40μm is shown in Fig 6A. It can be seen that surface of RTV sample is relatively flat, and only some small bumps and less obvious damage exists.

Level 2: The microscopic morphology of RTV samples in this level with a particle diameter of 40–100μm is shown in Fig 6B. And it is shown that the number of particles and holes increases obviously.

Level 3: The microscopic morphology of RTV samples in this level with a particle diameter more than 100μm is shown in Fig 6C. The surface morphology of RTV sample in this level is pretty rough, and RTV sample is nearly destroyed and huge holes and gullies can be found on the surface.

#### 2) Surface morphology

**Fig 7:**
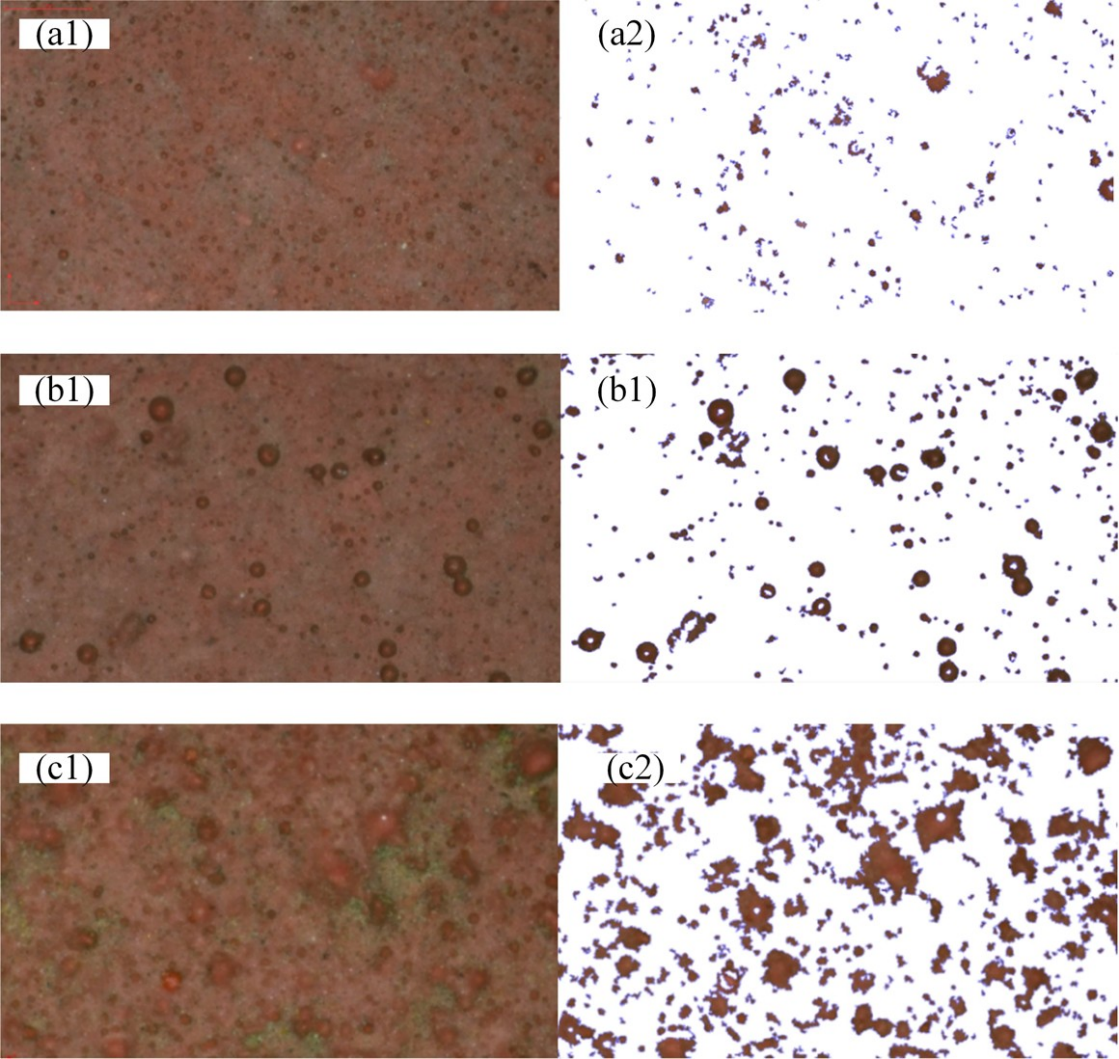
Particle size analysis. (a1), (b1), (c1) RTV pictures. (a2), (b2), (c2) Software analysis picture.

To further evaluate the surface state of RTV samples, surface morphological parameters should be acquired. A portable video microscope with the magnification ranging from 1 to 225 was used to test all the 20 samples. And the morphology pictures of RTV samples in each levels are acquired, and they are shown in Fig 7A1, 7B1 and 7C1, and representing level 1, 2 and 3. By adopting algorithms of image brightness enhancement function, image contrast function, image color adjustment function, image sharpening function and RGB color selection function, the pictures are proceed to acquire the particle size distribution. For example, Fig 7A1 becomes Fig 7A2 after the picture processing.

**Fig 8:**
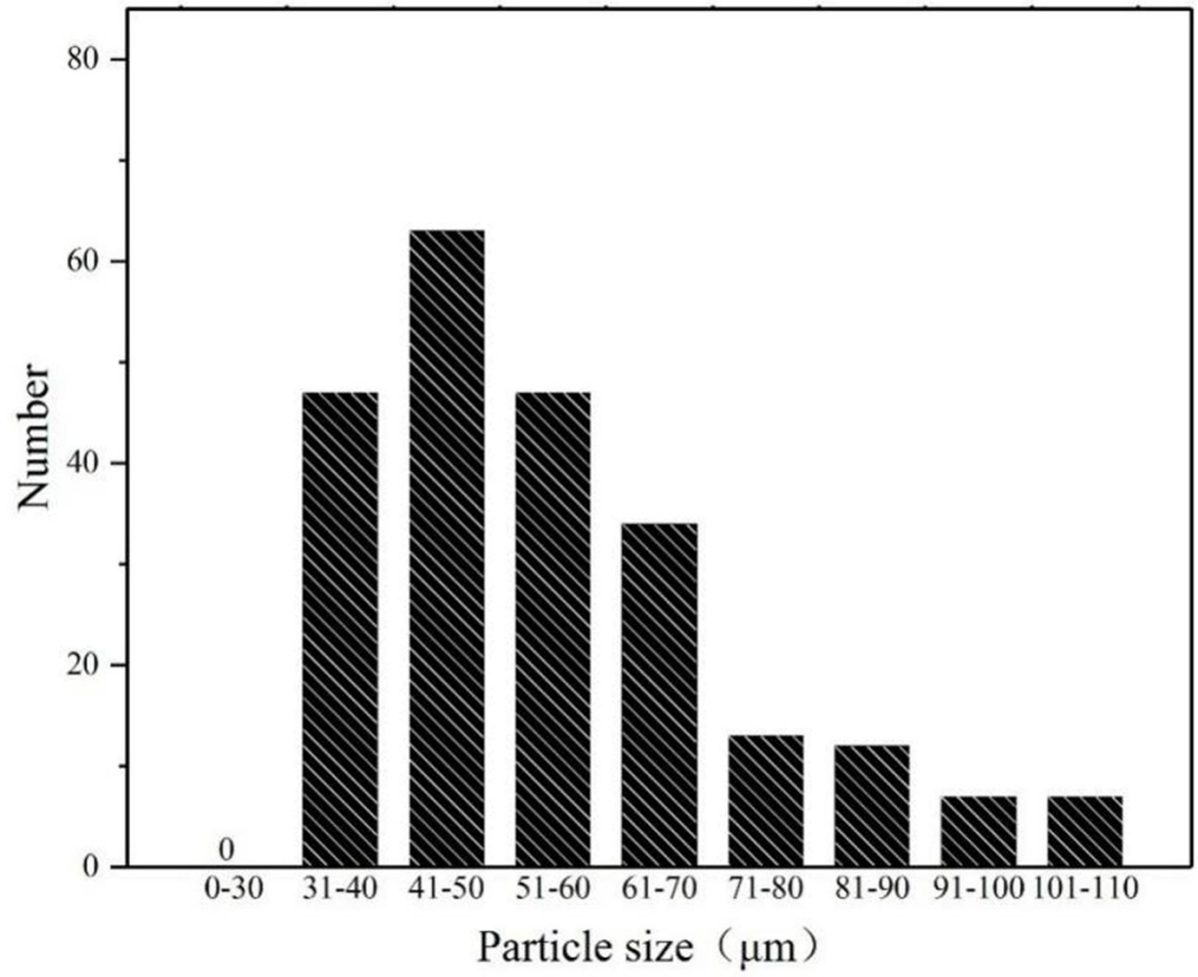
Average particle size distribution table.

The typical particle size distribution of Fig 7B is obtained and shown in Fig 8. It can be indicated that the particle diameter conforms to the law of normal distribution approximately. The average particle diameter is used to represent one RTV sample.

According to the observations in this section, comparison of average particle diameter method and SEM surface roughness method, the surface state of the sample less than 100μm observed by portable microscope is similar to the level 1 observed by SEM. The surface state of samples larger than 100μm observed by portable microscope is similar to the level 2 and 3 observed by SEM. It shows that the surface state proposed by the portable microscope used are consistent with the three aging classification proposed by SEM. And this method can be used as a supplement to the surface roughness method.

## 4 Chemical analysis of RTV samples

In the previous section, 20 pieces of RTV samples are divided into three different ageing levels according to their microscopic morphology (SEM). Multiple testing methods including element content analysis, FTIR analysis and dielectric properties analysis are conducted in this section. The chemical properties of RTV samples with different levels are also given in this section.

### 4.1. Element content

**Fig 9:**
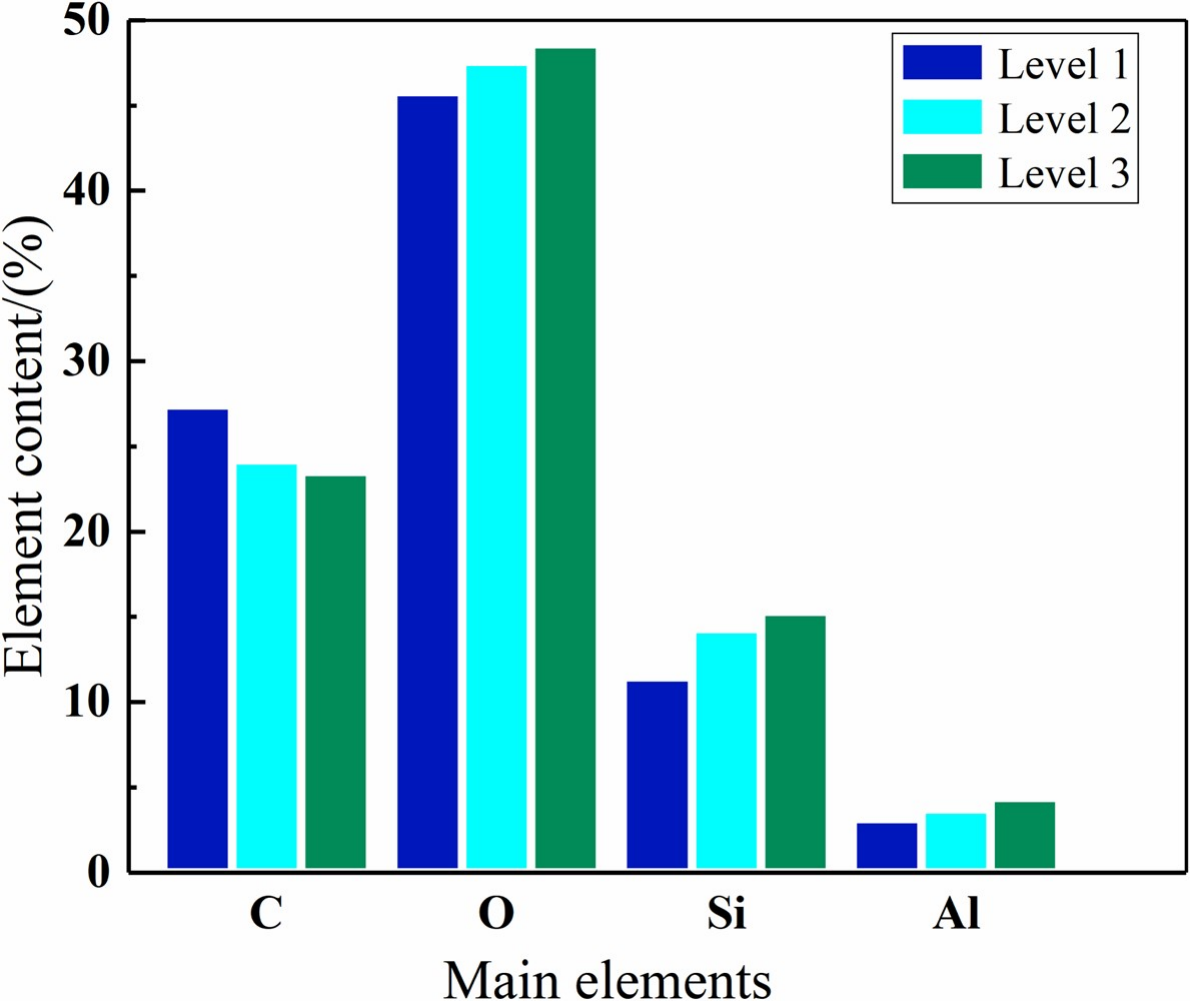
Element content ratio of the RTV samples at different ageing levels.

By analyzing the molecular structure of PDMS, the relative contents of elements C, Si, O, Al are selected as characteristic element. The average relative content of these four elements at three different ageing levels are shown in Fig 9. It can be seen from Fig 9 that the contents of four characteristic elements in three levels are quite different. The contents of O, Si and Al shows a growth trend the RTV sample aging level increases, while the content of C element shows a decline trend. It is indicated that C element in the molecular chain of the samples decreases while O element increases at the same time, which is caused by the serious oxidation with aging level increases [19]. The change of Al is mainly related to the leakage of ATH [12].

**Fig 10:**
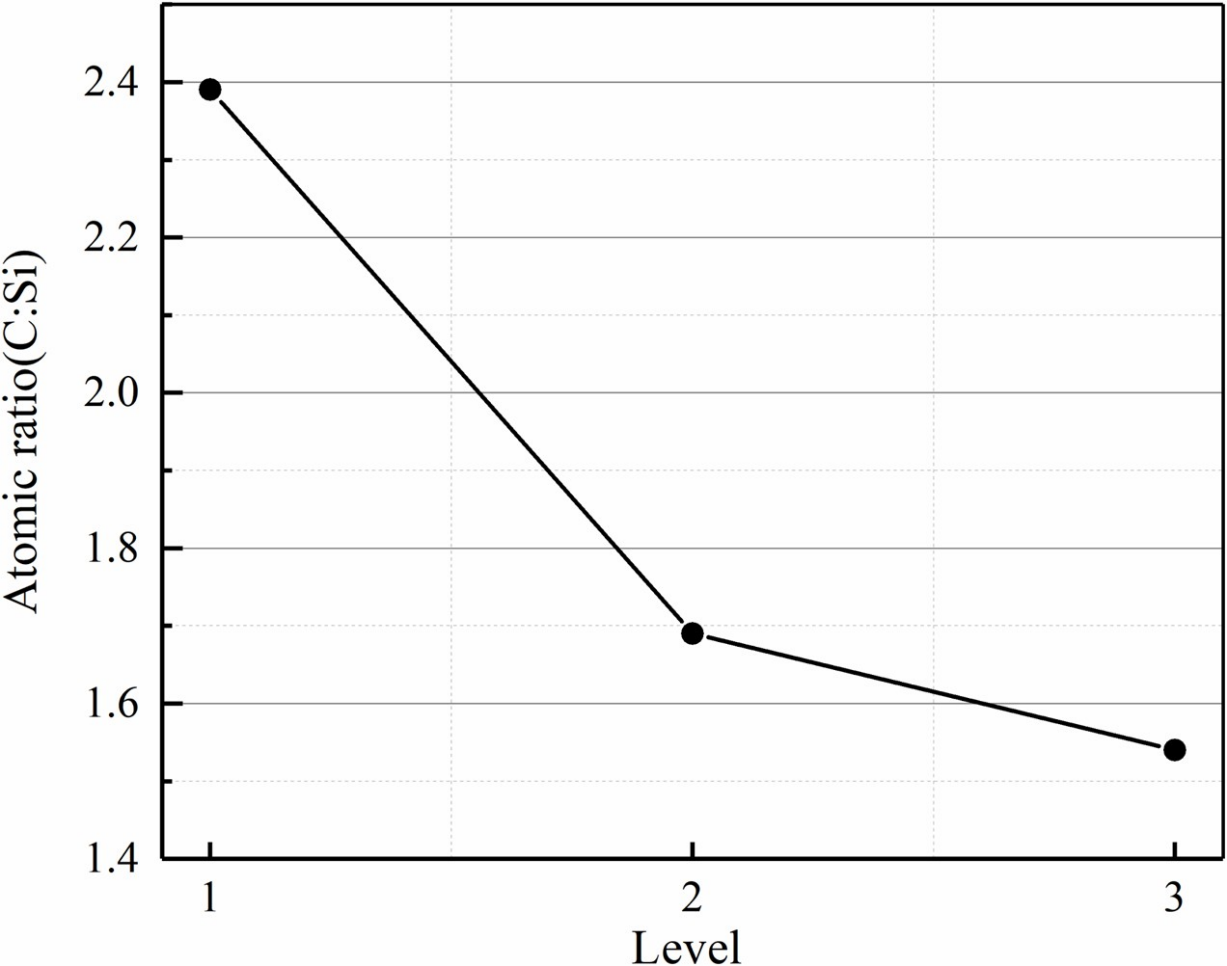
Atomic ratio (C:Si) of the RTV samples at different ageing levels.

According to the PMDS molecular formula, the relative ratio of C:Si should be more than 2, which is consistent with the results of EDS analysis. The average content ratio of C:Si of RTV samples with different ageing levels are shown in Fig 10. With increasing ageing level of the RTV samples, the ratio of C:Si drops from 2.39 to 1.54 significantly. The ratio of C:Si is less than 2 at level 2 (1.69) and level 3 (1.54), which proves that the molecular structure of the RTV surface has been severely damaged. Hence, the RTV coating is suggested to be replaced or recoated when the roughness of the RTV coating reaches 40μm or above.

### 4.2. FTIR analysis of RTV samples

**Fig 11:**
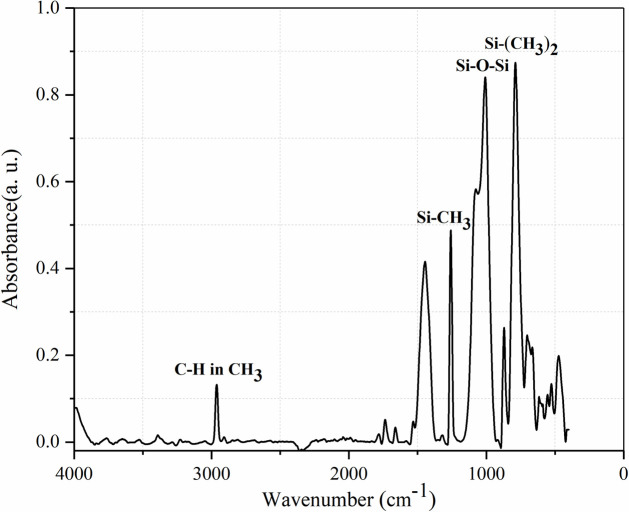
FTIR spectrum of distribution of main chemical groups in the RTV samples.

The main components of this type of RTV formula are PDMS, Aluminum Trihydrate (ATH) and Silica. In FTIR, the aging level of RTV can be evaluated by the absorbance peaks of several chemical groups. The absorbance of different chemical groups are shown in Fig 11, and the appropriate wave number of different chemical groups are shown in Table 3.

**Table 3:** Appropriate wave numbers of chemical groups.

Wave number (cm^-1^)	Chemical groups
750–850	Si-(CH_3_)_2_
1000–1100	Si-O-Si
1255–1270	Si-CH_3_
2960–2965	C-H in CH_3_

**Fig 12:**
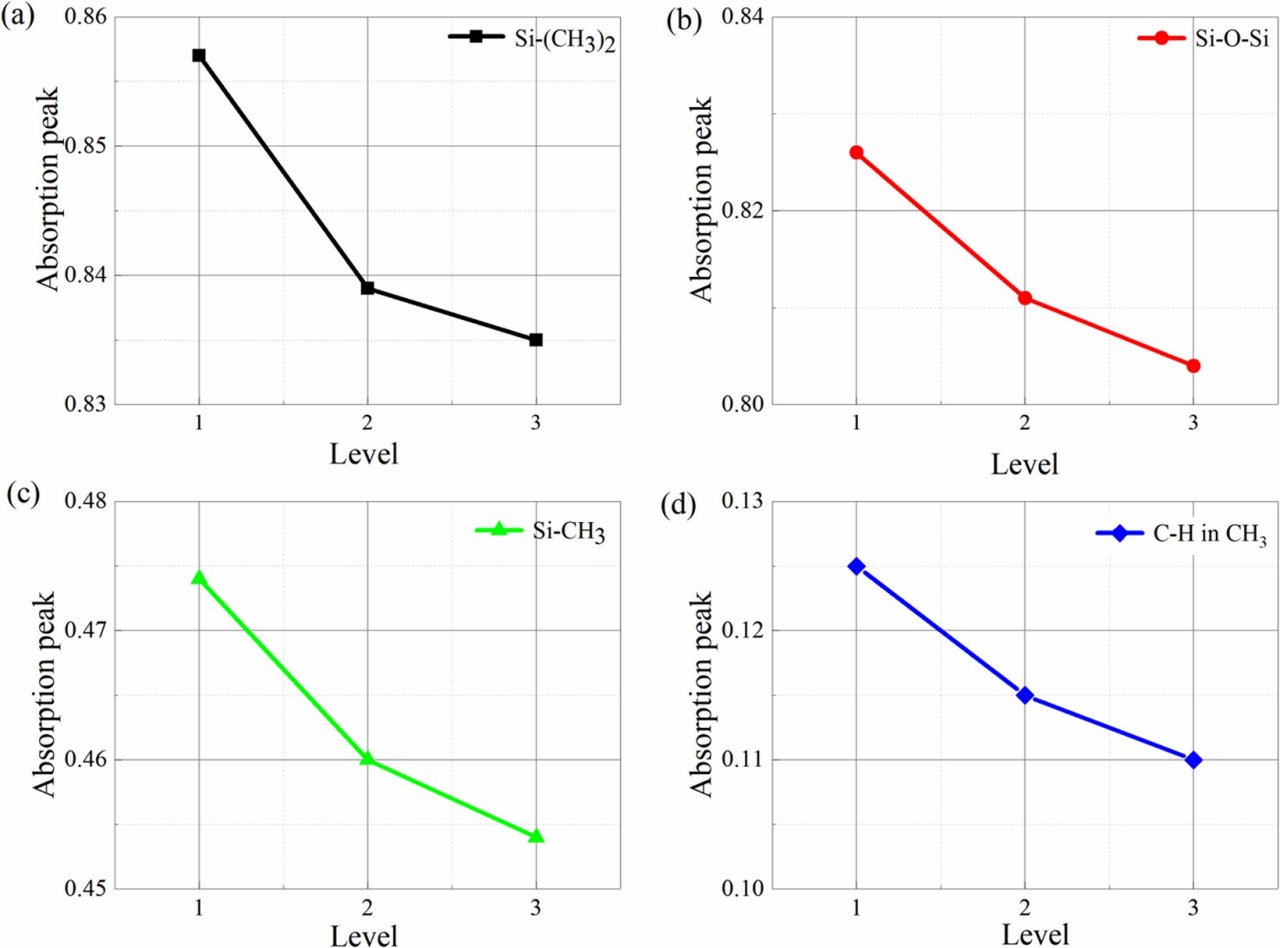
Change of absorption peak in chemical groups with ageing level. (a) absorption peak change graph of Si-(CH_3_)_2_; (b) absorption peak change graph of Si-O-Si; (c) absorption peak change graph of Si-CH_3_; (d) absorption peak change graph of C-H in CH_3_.

The average absorption peaks of four chemical groups Si-(CH_3_)_2_, Si-O-Si, Si-CH_3_, C-H in CH_3_ of RTV samples under different ageing levels are analyzed, as shown in Fig 12. It is indicated that absorption peaks of the four chemical groups decreases with the ageing level increases. It can be concluded that the difference of absorption peaks between level 1 and 2 is much larger than the difference between level 2 and level 3. When the aging level changes from level 1 to level 2, the small particle groups and holes appears, resulting in the accumulation of contamination and moisture. In this case, corona discharge will occur under high humidity condition, leading to the destruction of the molecular structure of the RTV material [20]. When the aging level changes from level 2 to level 3, large holes and cracks occurs, resulting in the accumulation of a lot of moisture. This may lead to the production of trace amounts of nitric acid, which is dominant in the change of level 2 to level 3 [21].

### 4.3. Dielectric properties

Two RTV samples from each aging level are chosen for the dielectric spectroscopy analysis. Three parameters-dielectric constant, dielectric loss and conductivity are obtained under the test condition 20°C and 50Hz, as shown in Table 4.

**Table 4:** Two method correspondence table.

Aging level	Sample number	Dielectric constant	Dielectric loss	Conductivity (S/cm)
**Level 1**	1	6.11	3.62×10^−3^	6.14×10^−13^
	2	5.11	5.75×10^−3^	8.17×10^−13^
**Level 2**	3	3.57	4.19×10^−3^	4.16×10^−13^
	4	9.33	3.38×10^−3^	8.76×10^−13^
**Level 3**	5	8.08	4.98×10^−3^	1.12×10^−12^
	6	3.91	2.04×10^−3^	2.22×10^−13^

It can be seen from Table 4 that the dielectric properties of RTV samples shows no obvious rule with the change of aging levels. The results are quite different from former researches [22]. Compared with the laboratory experiments, the environment where the actual RTV coatings are located is much more complicated. The aging process is formed due to the combined effect of many factors, and this will be further studied in our further study.

## 5 Conclusion

In this paper, the experiment focused on the aging characteristics and evaluation methods of RTV silicone rubber in high humidity area. In this experiment, the hydrophobicity, surface morphology, surface microtopography and physicochemical properties of 20 RTV were analyzed. Based on the microstructure of RTV surface, the new classification methods are proposed. Through a variety of complex analysis methods for RTV samples, the main findings are summarized below.

This paper proposes two RTV surface state classification methods. The first method is based on SEM’s surface roughness method (×1000) in more detail. According to the particles, holes and cracks on the RTV surface under 1000 times, the RTV aging state is divided into three levels. Other method is the average particle diameter analysis method based on a portable microscope (×50), this method divides the RTV aging state into three levels based on the RTV surface particle diameter.

As the surface roughness and the average particle diameter of RTV increases, atom content of C, O, Si and Al and the ratio of C:Si of RTV coating change significantly. The ratio of C:Si drops to 1.69 at level 2, indicating that the PMDS molecular structure is severely damaged. In addition, the absorption peaks of chemical groups such as Si-(CH_3_)_2_, Si-O-Si, Si-CH_3_ and C-H in CH_3_ gradually decrease with increasing roughness level of RTV. Therefore, the surface roughness level is effective to reflect the ageing level of RTV.

This study can be used to predict the life of RTV based on its surface state, the study also can provide an effective engineering advice for the substations to replace or recoat the RTV on insulators.

## Supporting information

S1 Dataset (DOCX)
