# Supplementary material for: Study on ageing characteristics and evaluation methods of RTV silicone rubber in high humidity area
Source: PLoS One. 2021 Jun 4;16(6):e0251092. doi: 10.1371/journal.pone.0251092 (PMC8177440; doi:10.1371/journal.pone.0251092)

**Hydrophobicity Test Results**

| No. | Static contact angle (°) |
| --- | --- |
| **1** | 129.31 |
| **2** | 89.5 |
| **3** | 127.1 |
| **4** | 133.01 |
| **5** | 130.11 |
| **6** | 130.91 |
| **7** | 128.52 |
| **8** | 129.69 |
| **9** | 133.01 |
| **10** | 127.1 |
| **11** | 87.18 |
| **12** | 109.27 |
| **13** | 119.76 |
| **14** | 130.11 |
| **15** | 144.5 |
| **16** | 136.78 |
| **17** | 133.01 |
| **18** | 134.61 |
| **19** | 127.1 |
| **20** | 139.65 |

**SEM Test Results**

Sample No.1


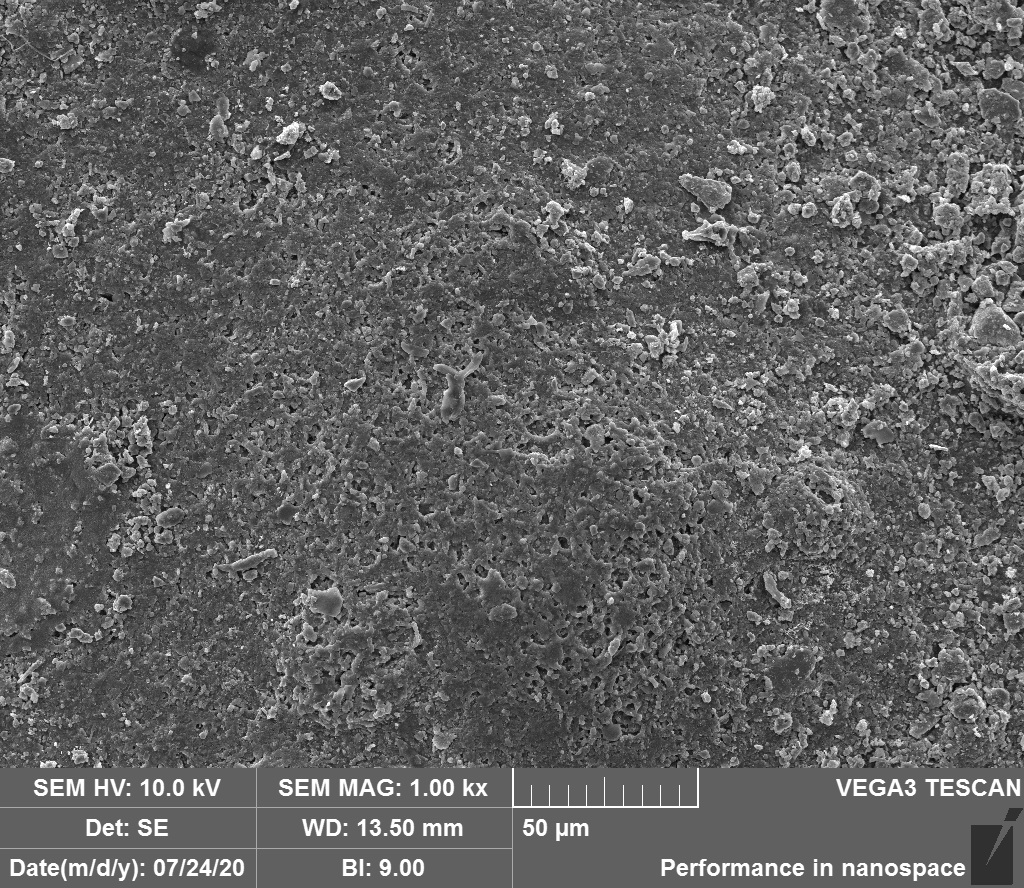


Sample No.2.


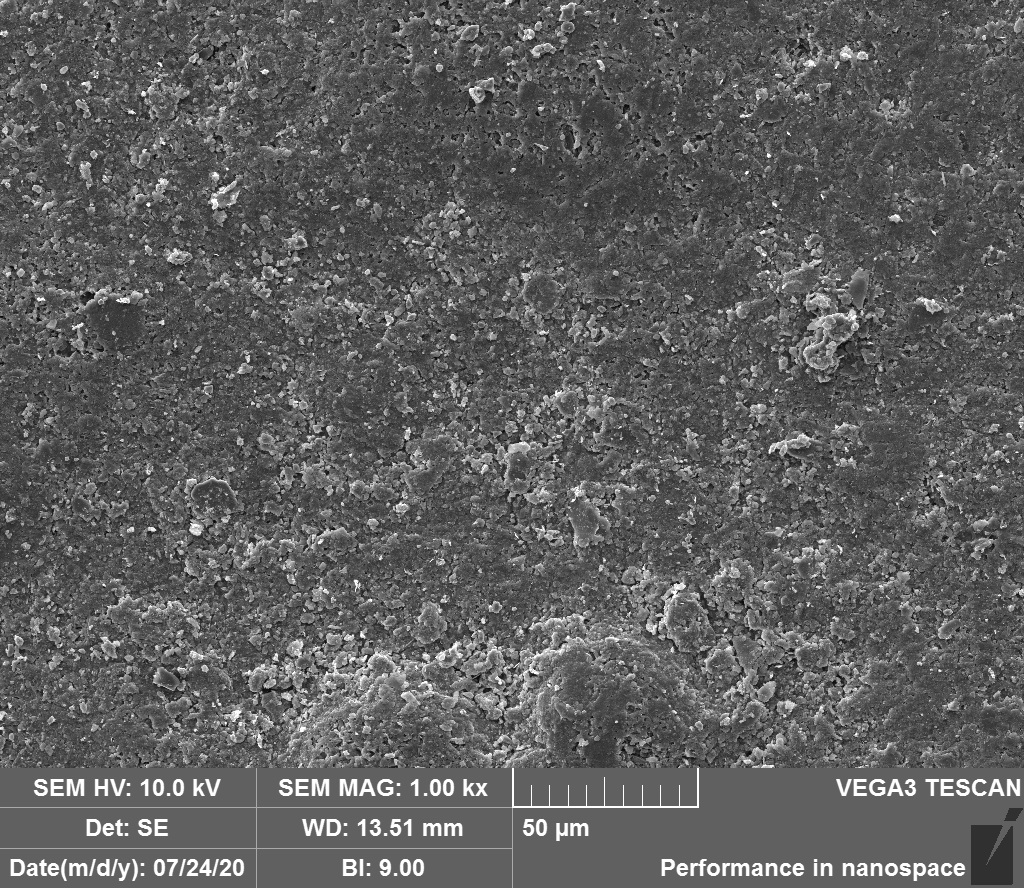


Sample No.3.


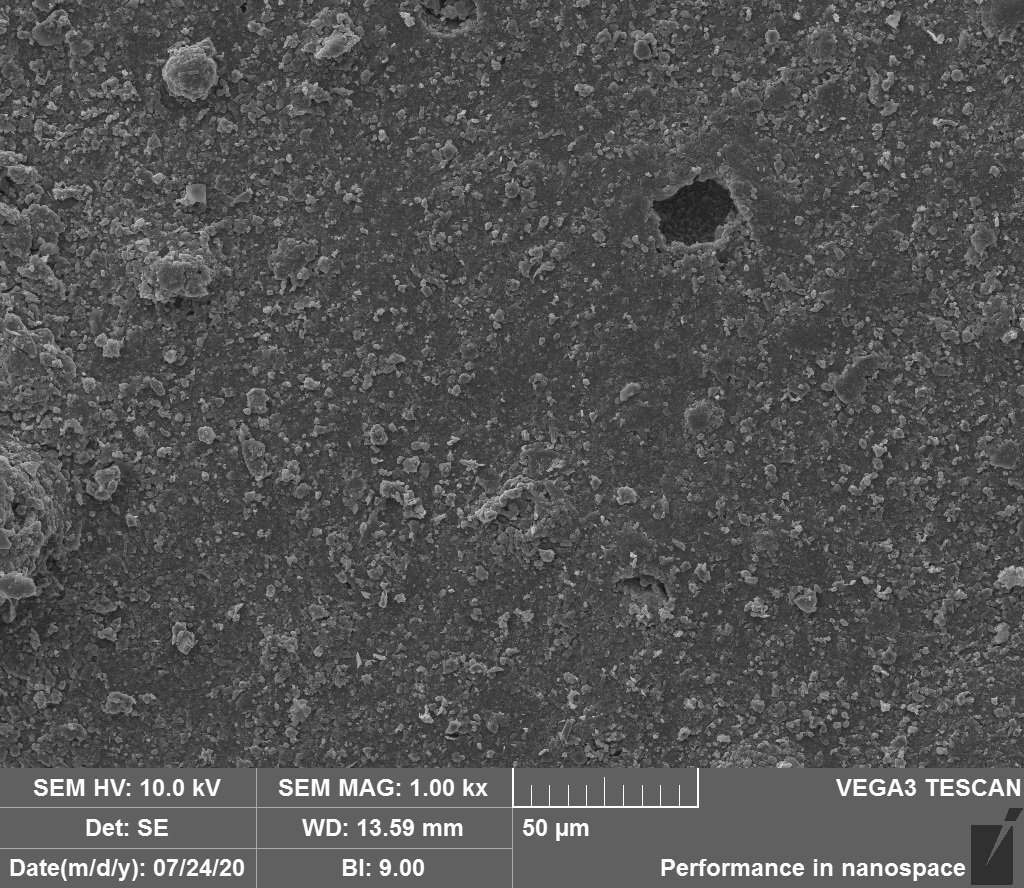


Sample No.4.


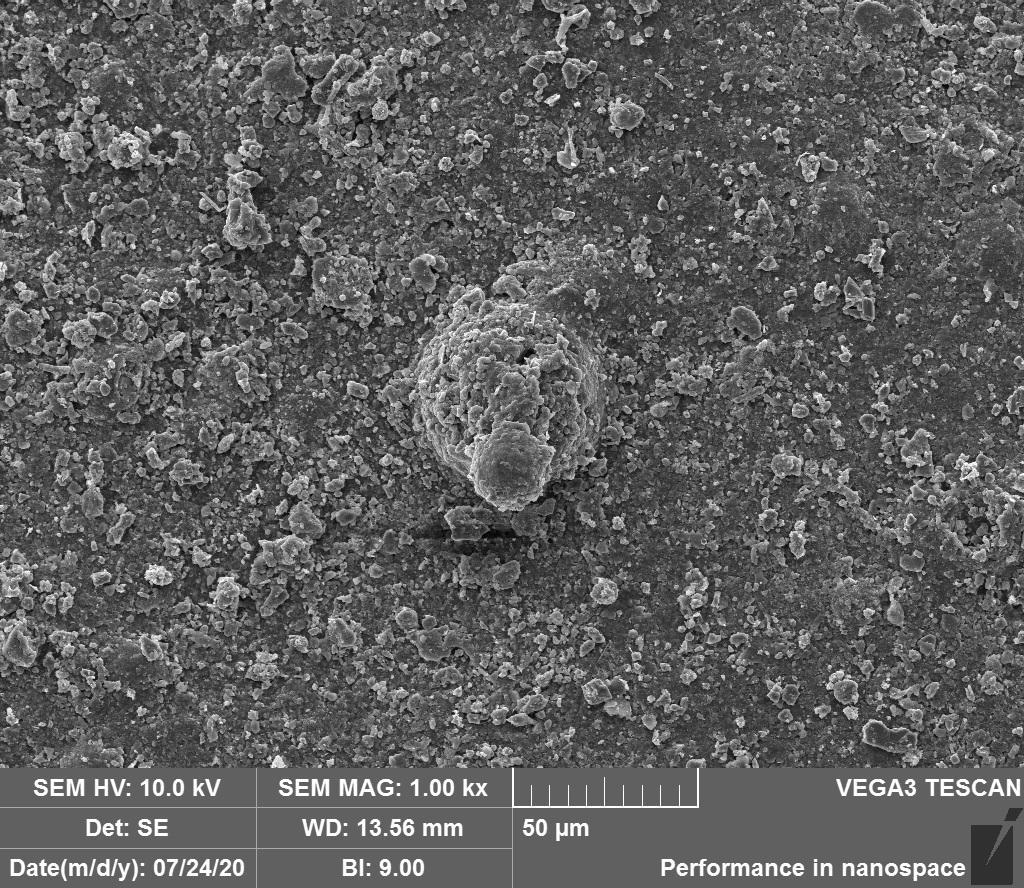


Sample No.5.


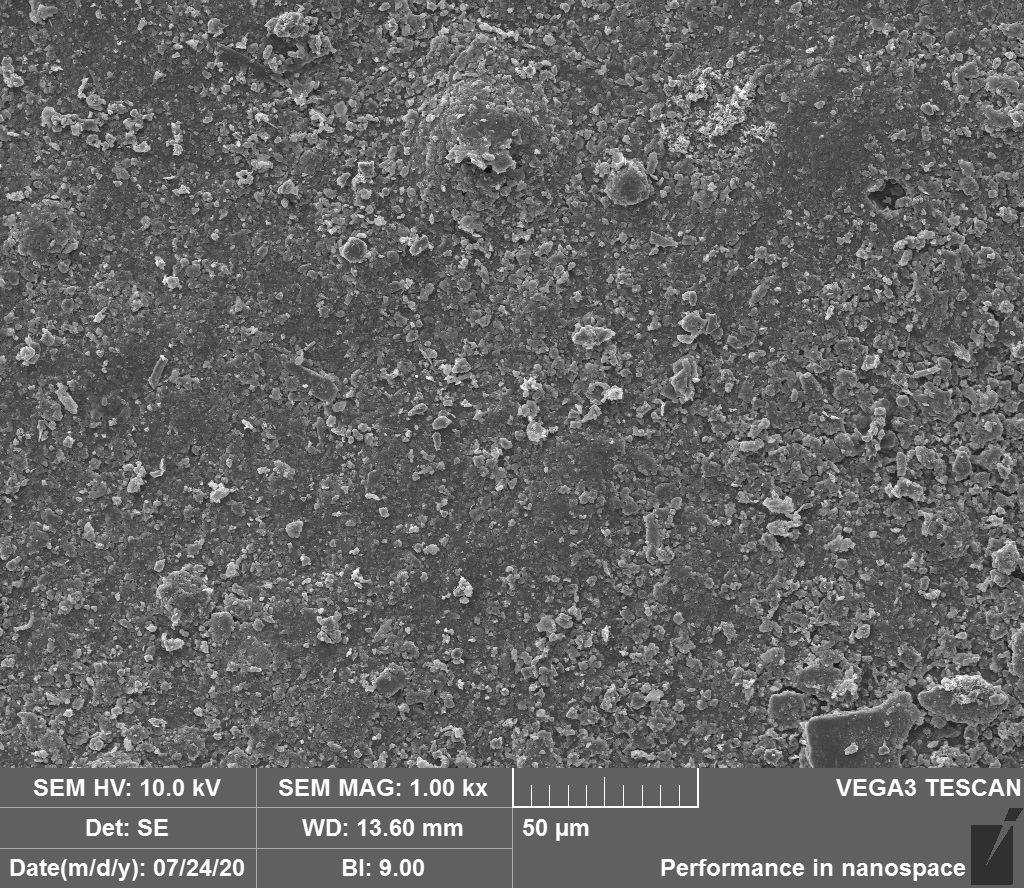


Sample No.6.


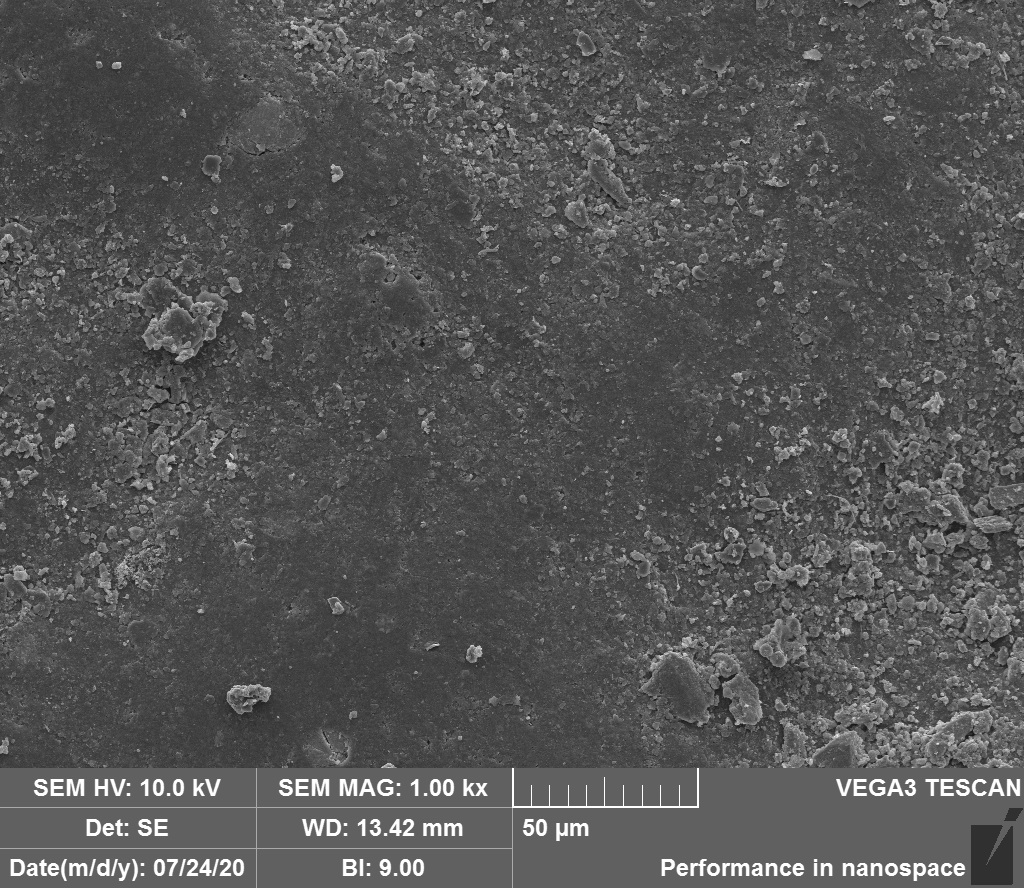


Sample No.7.


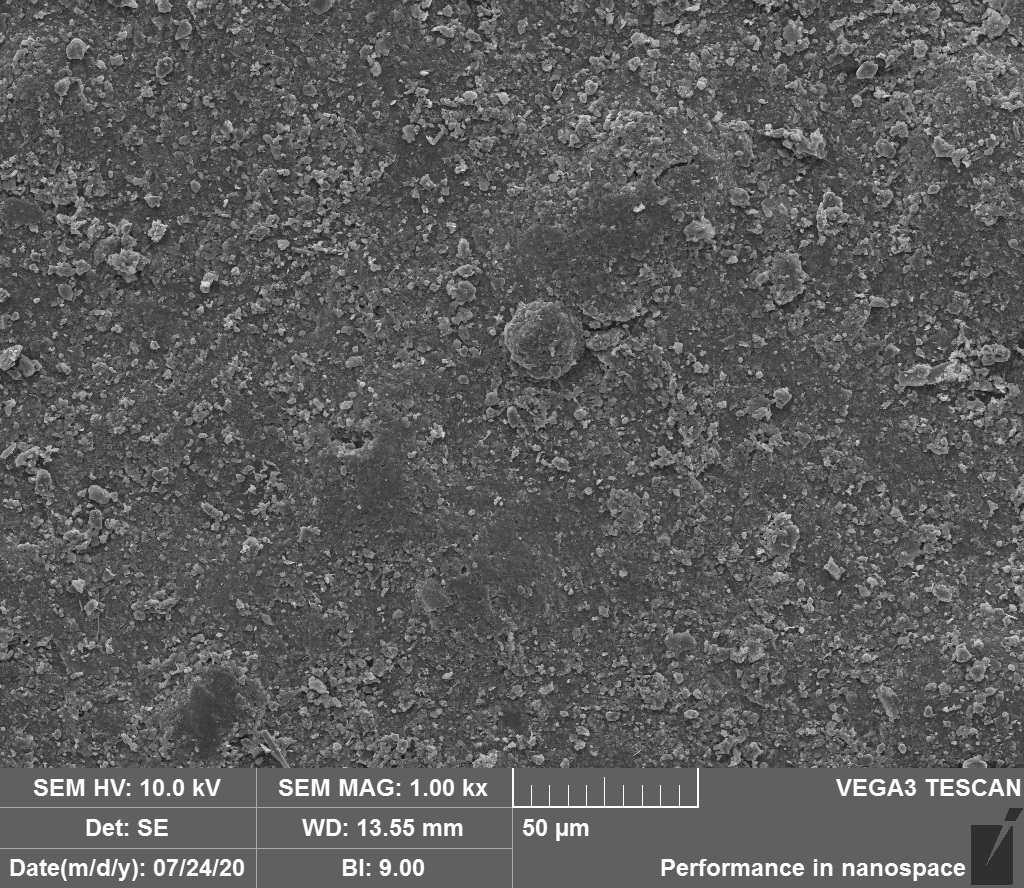


8.


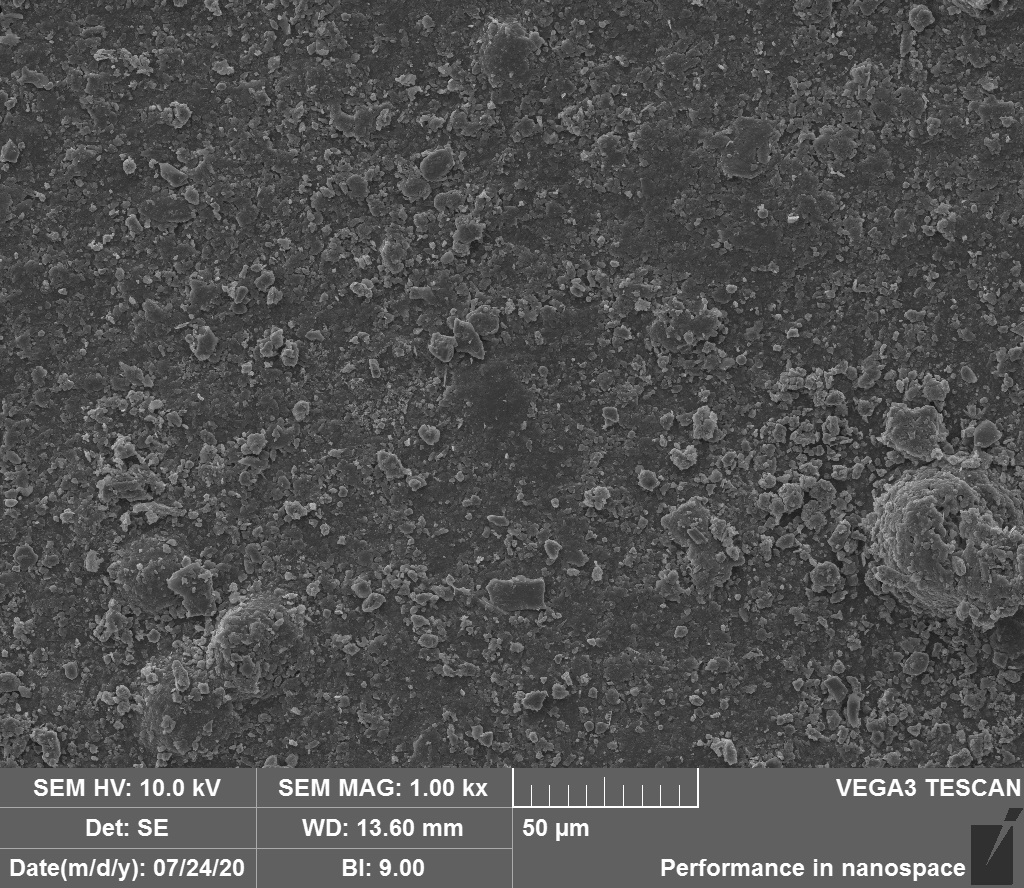


Sample No.9.


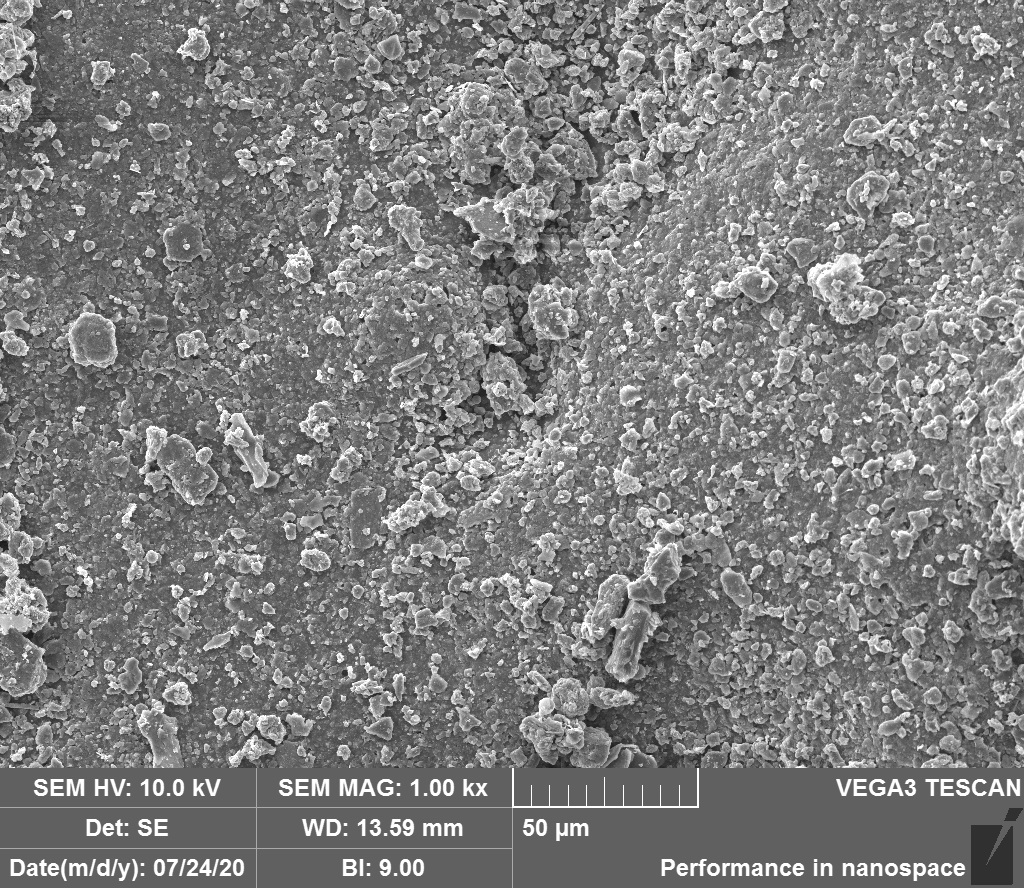


Sample No.10.


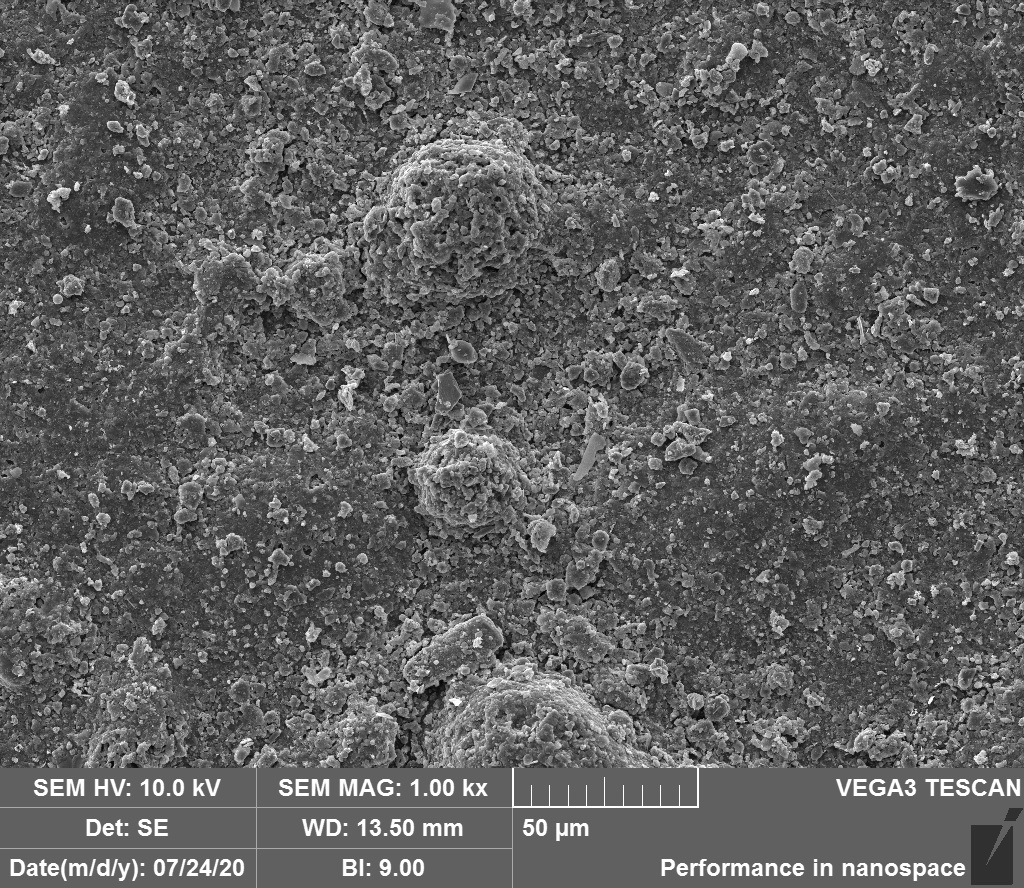


Sample No.11.


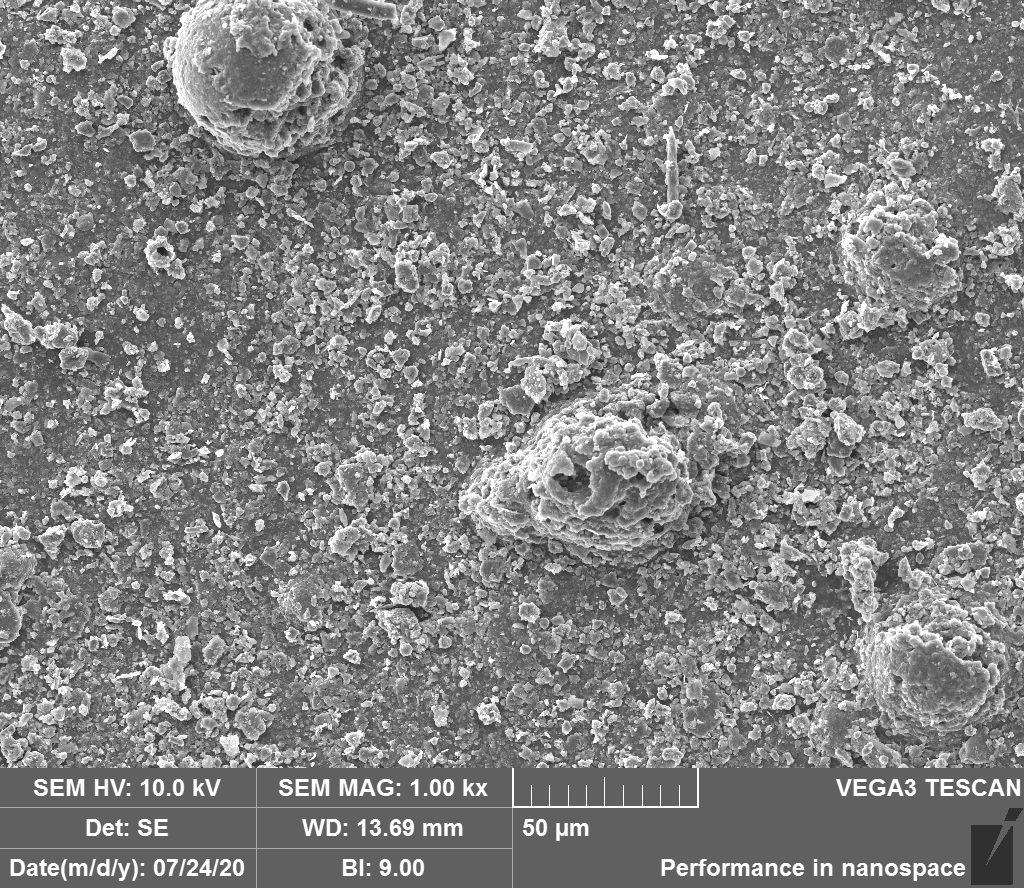


Sample No.12.


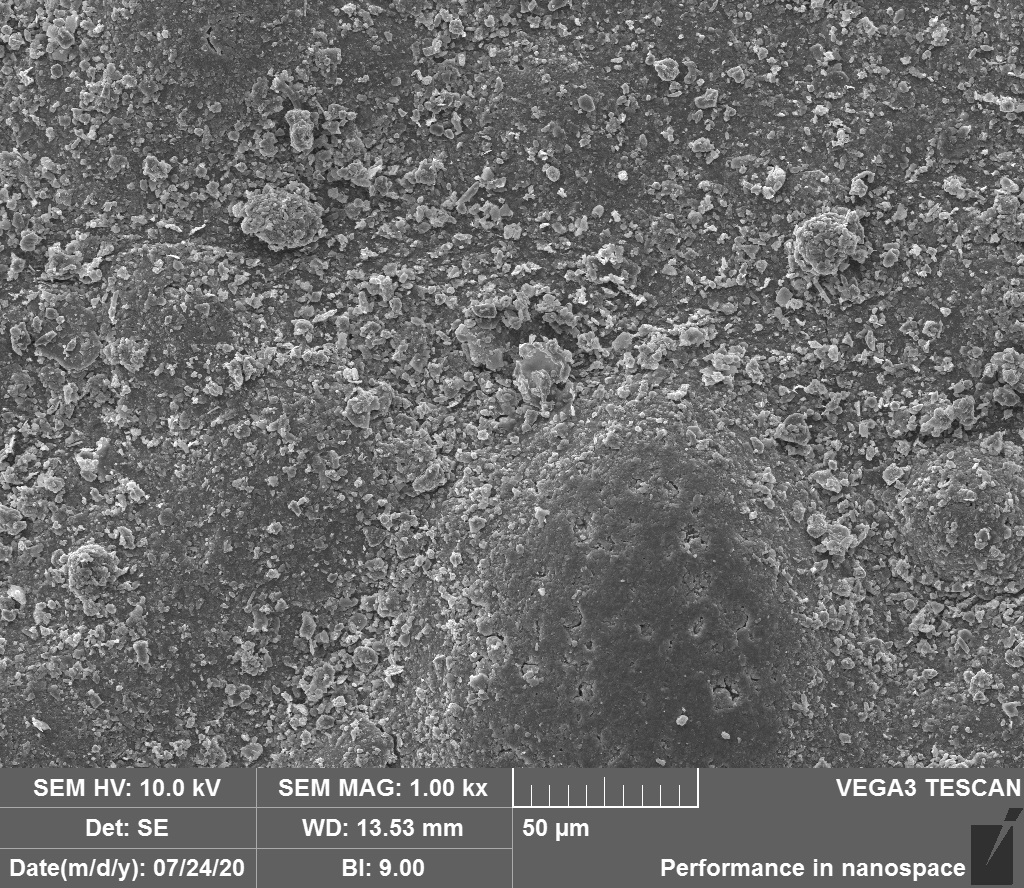


Sample No.13.


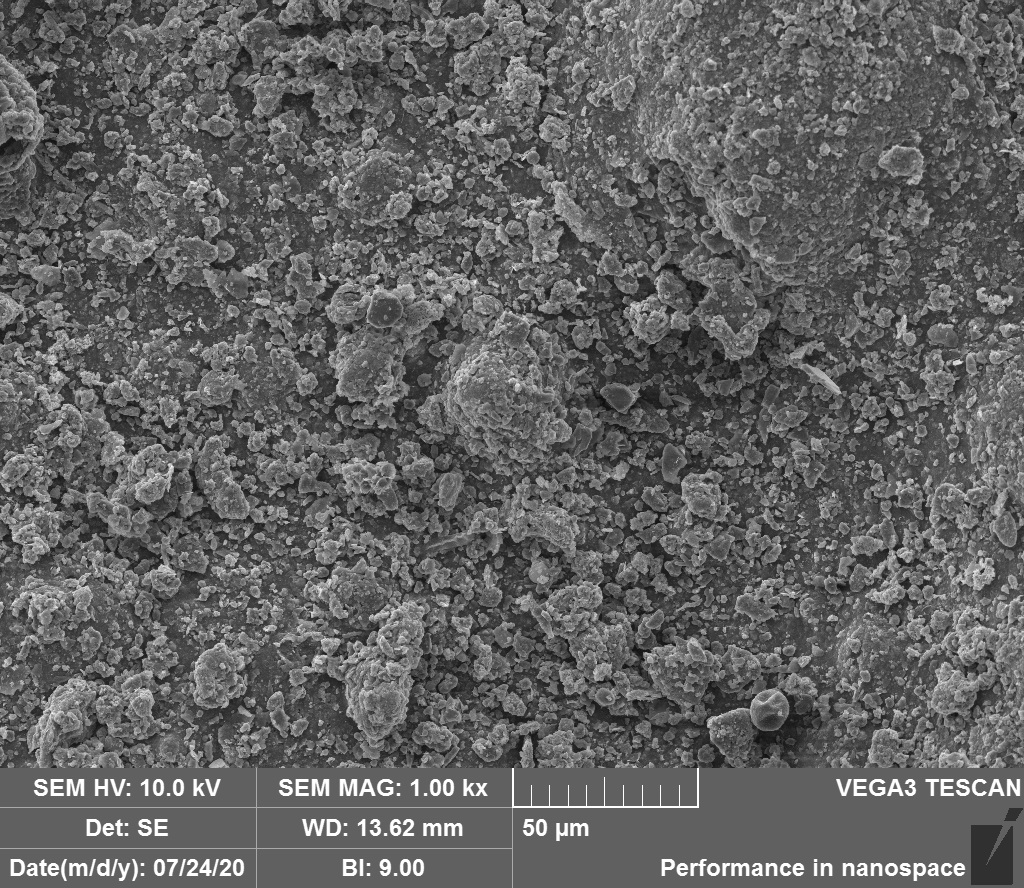


Sample No.14.


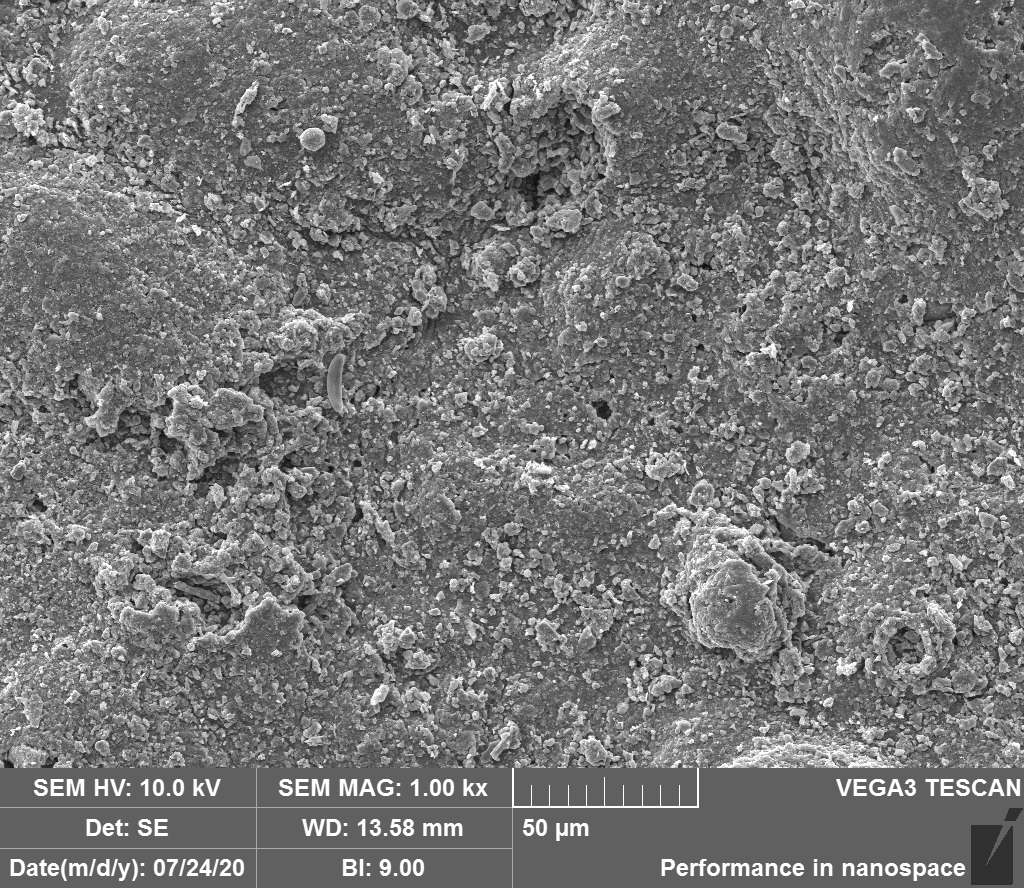


Sample No.15.


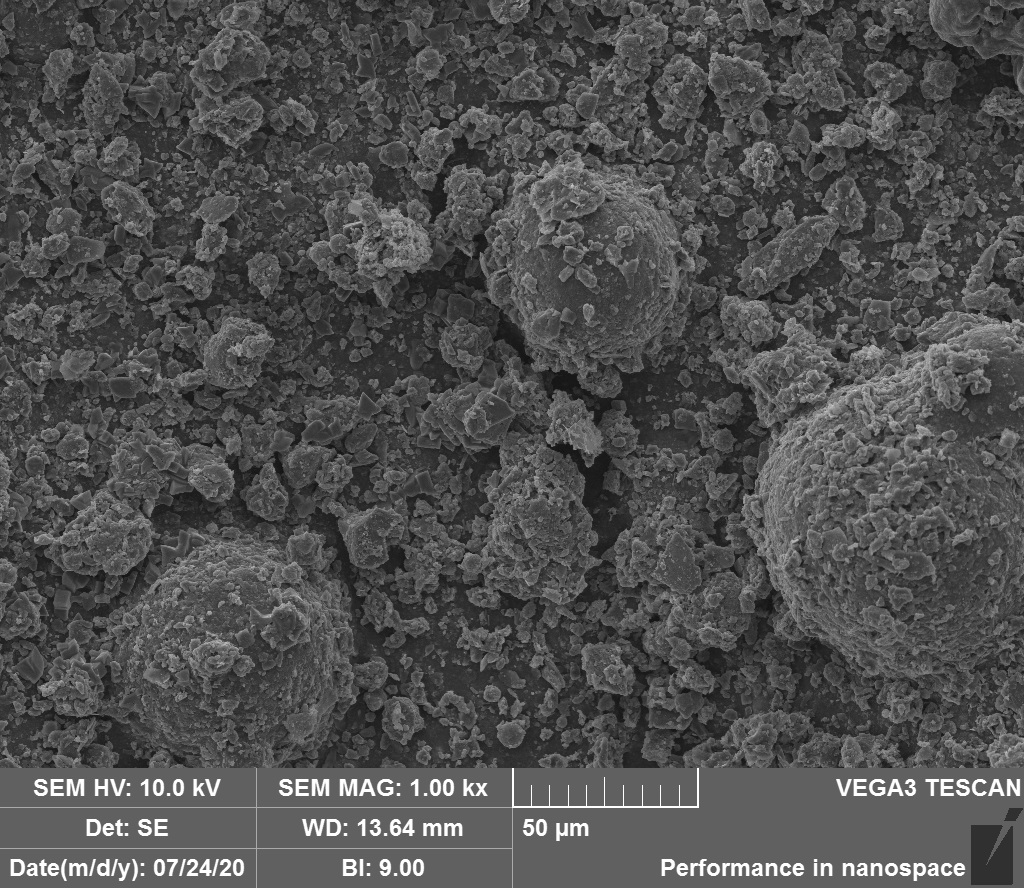


Sample No.16.


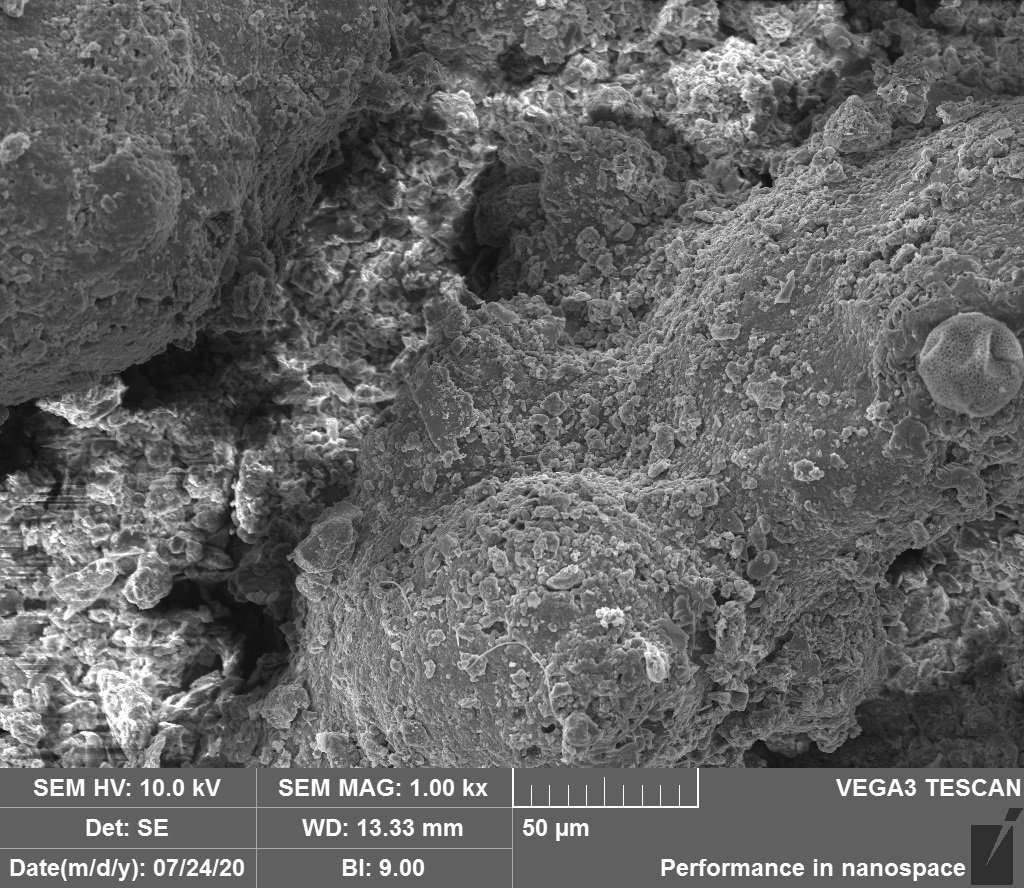


Sample No.17.


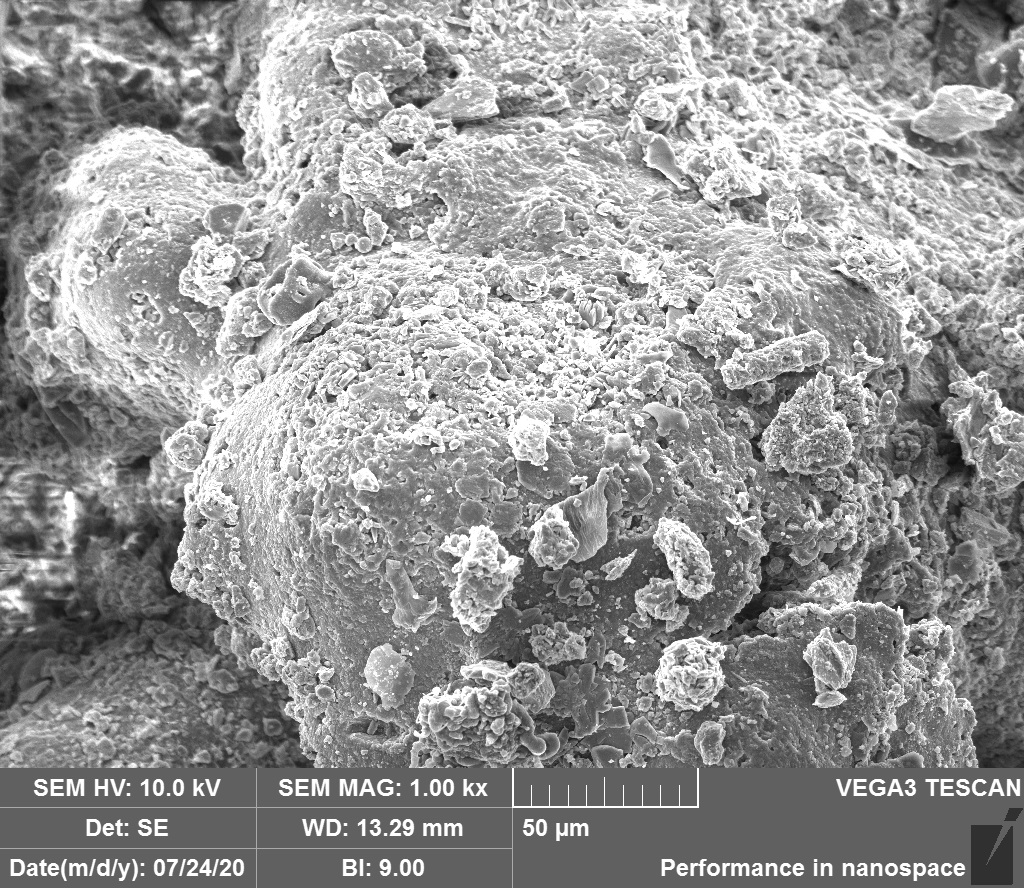


Sample No.18.


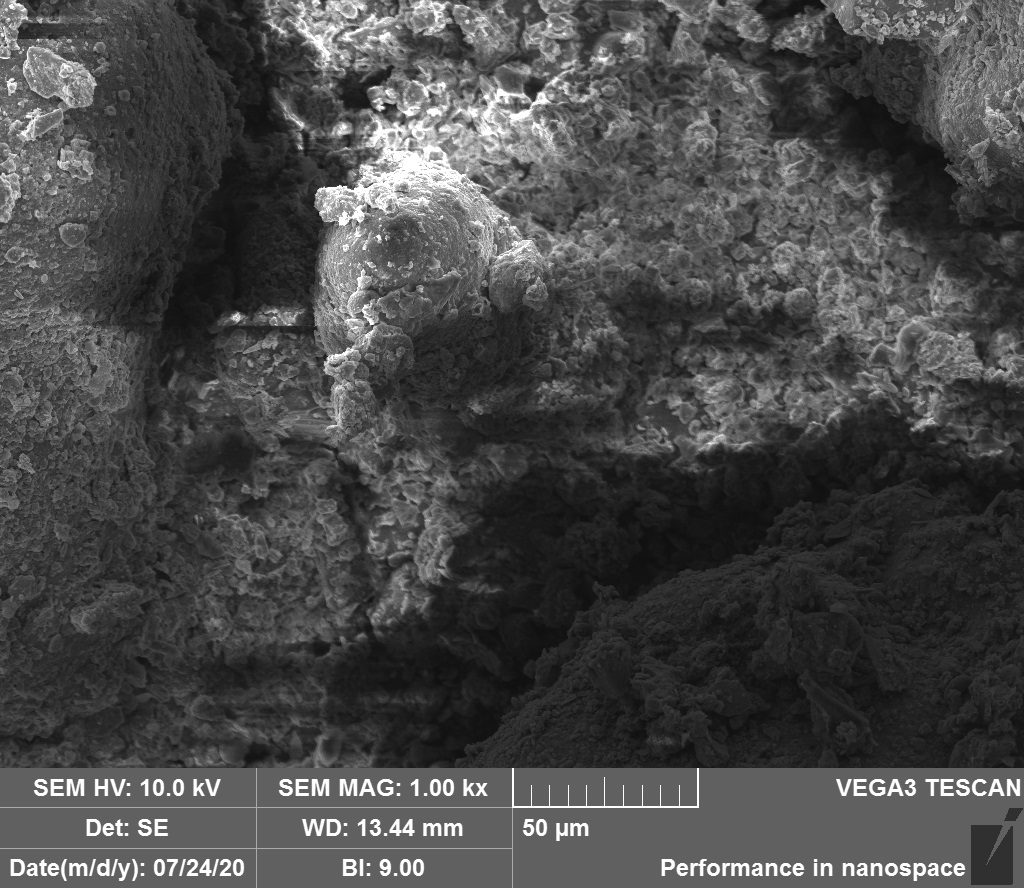


Sample No.19.


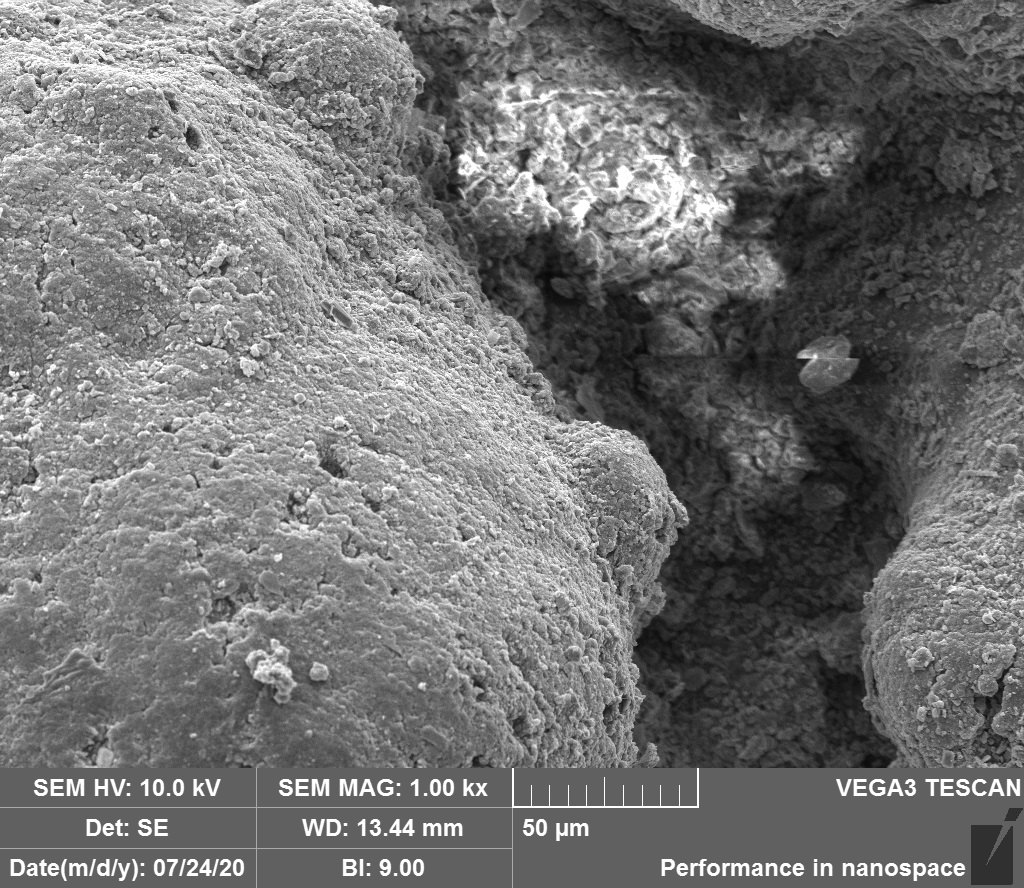


Sample No.20.


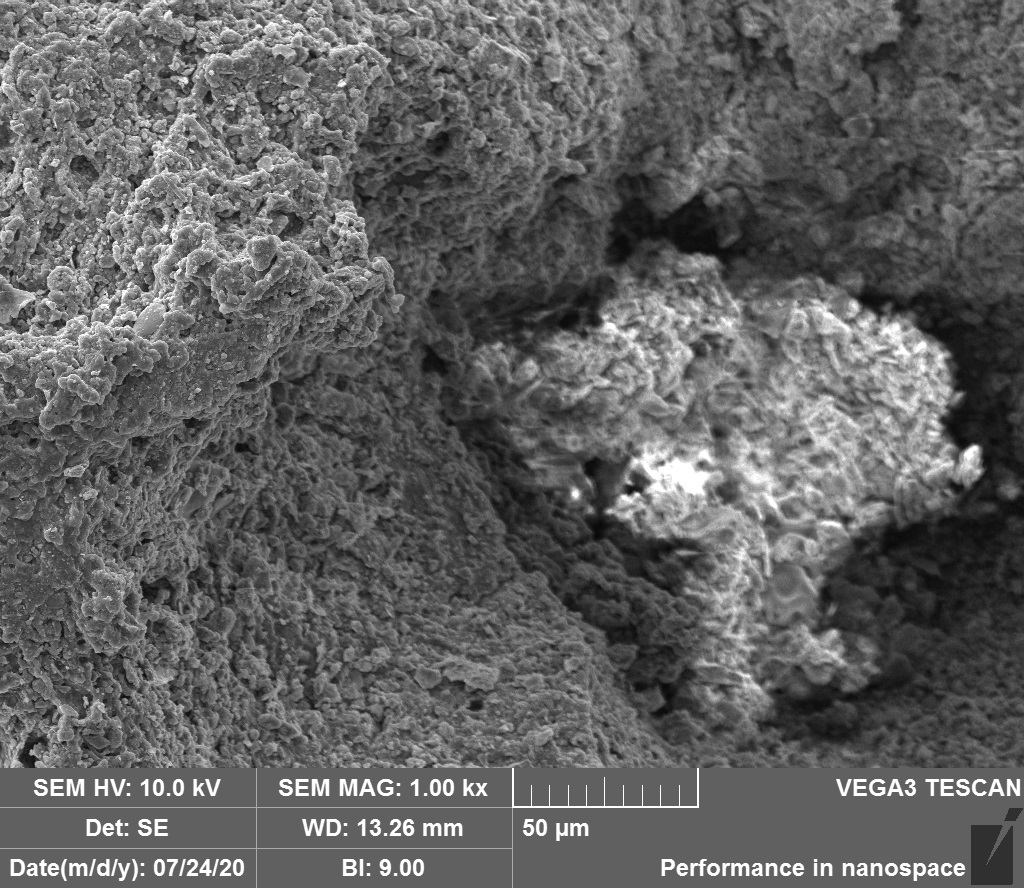


**EDS Test Results**

Sample No.1.


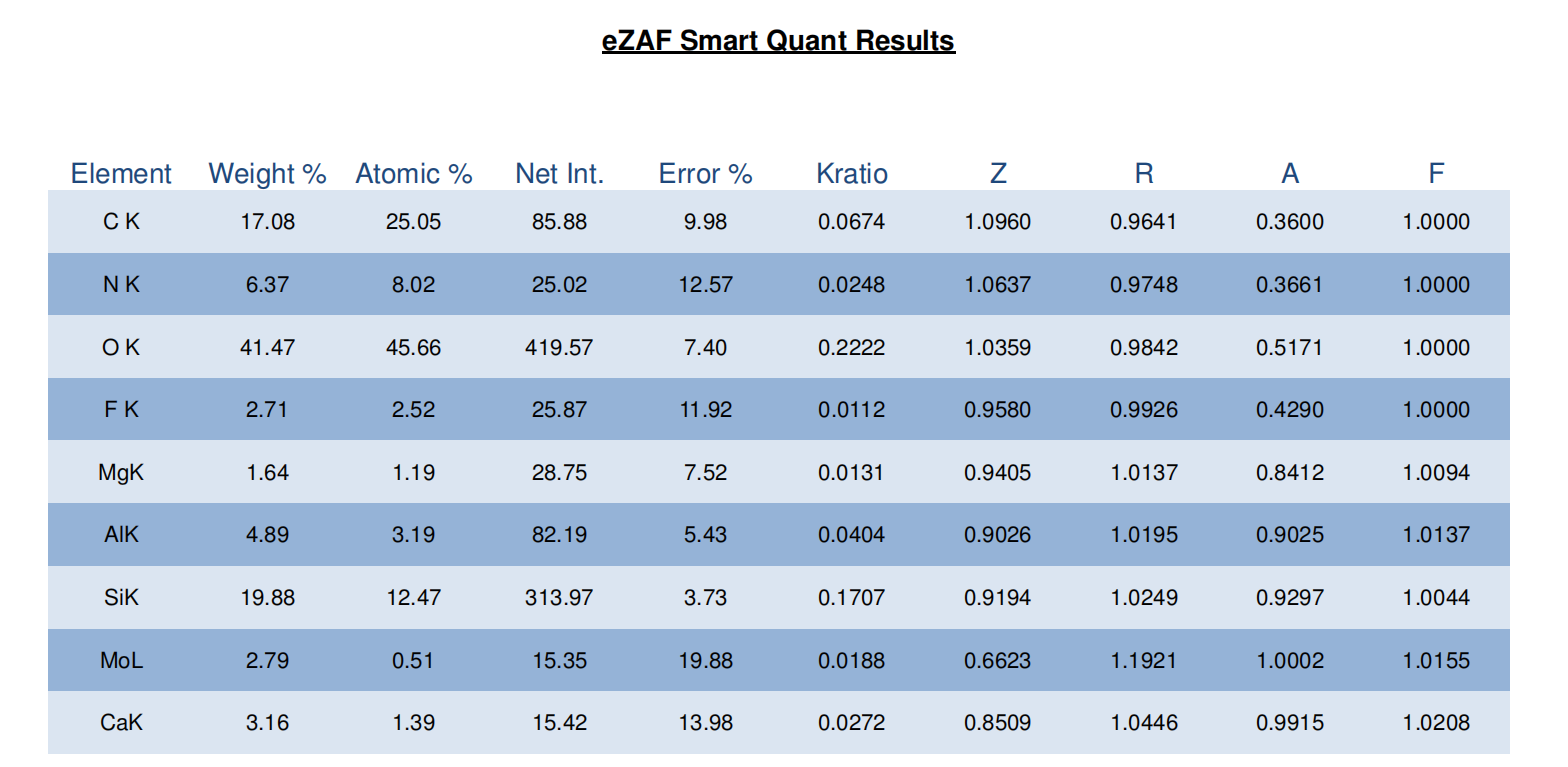


Sample No.2.


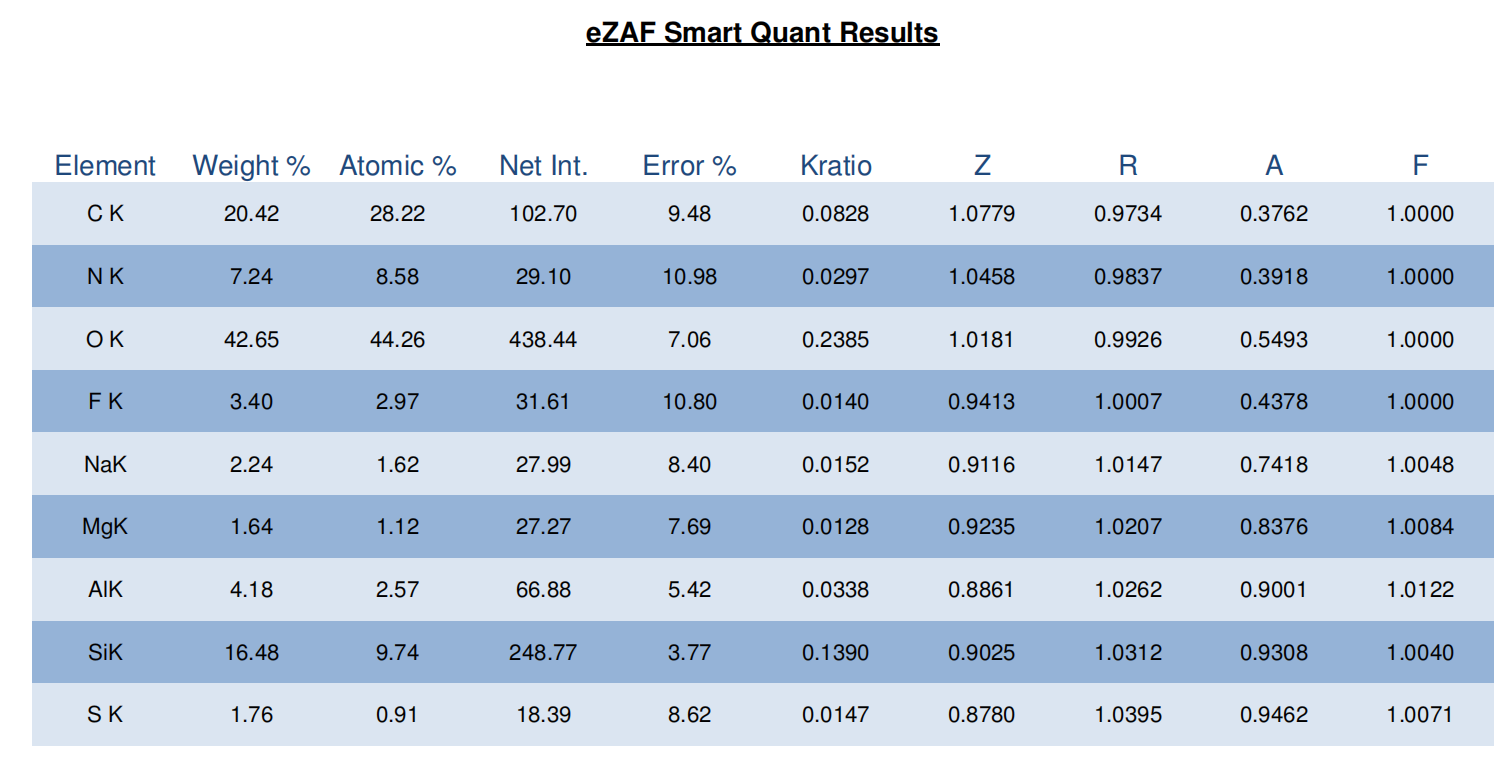


Sample No.3.


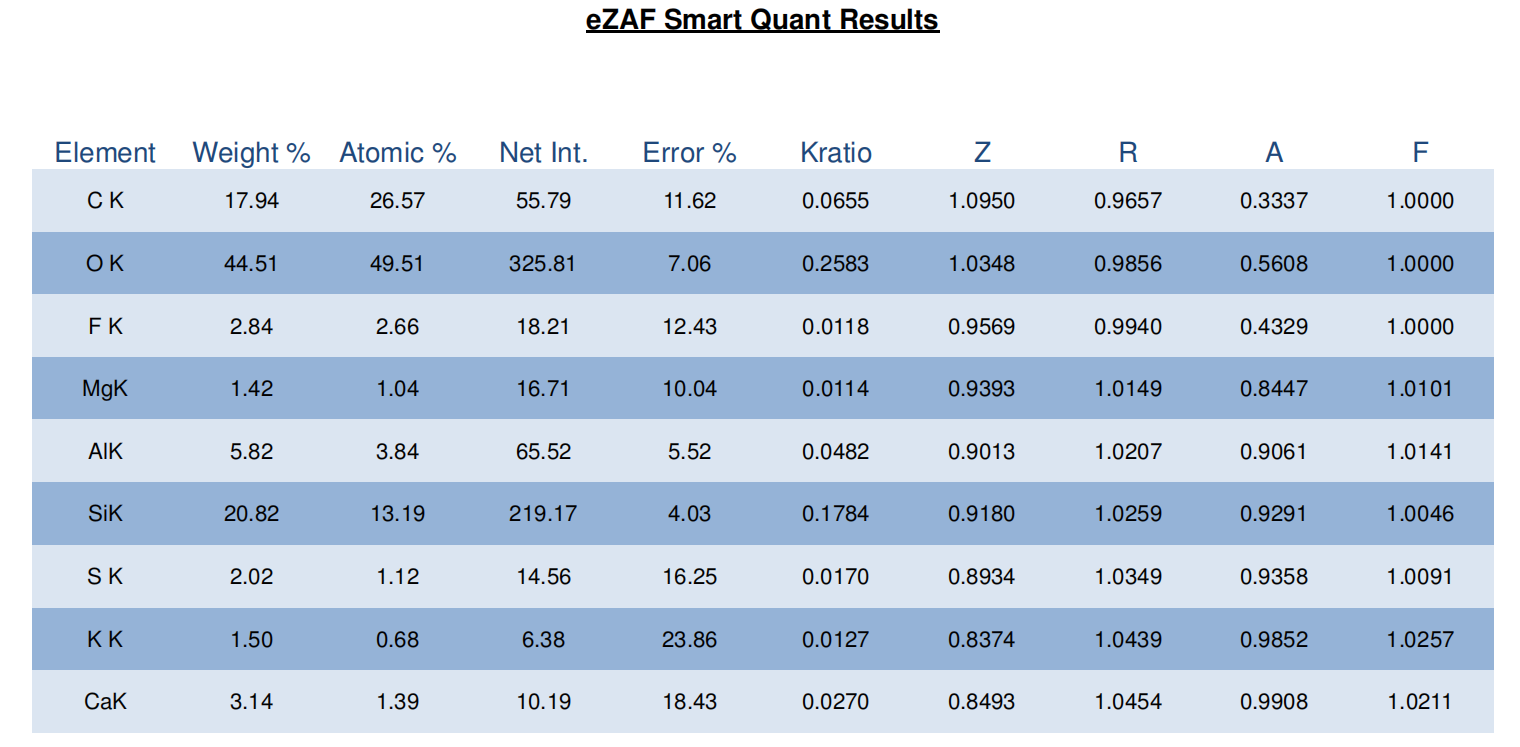


Sample No.4.


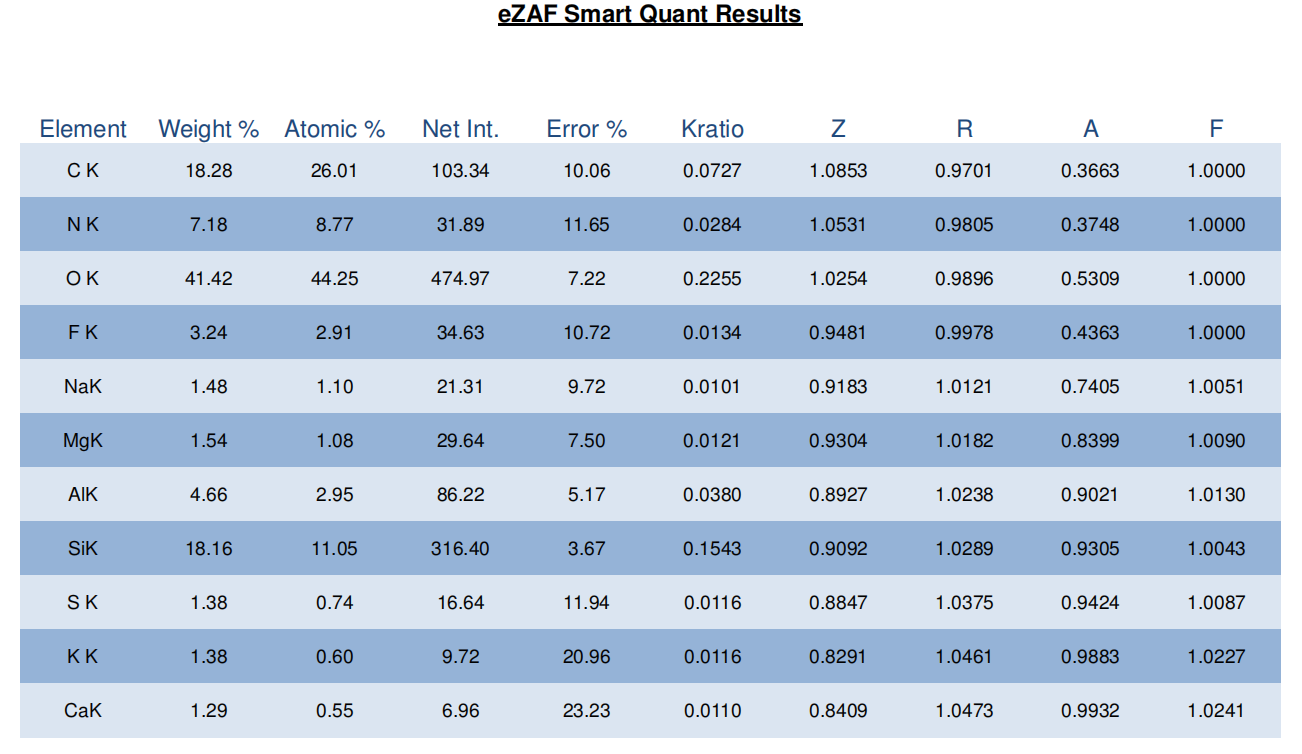


Sample No.5.


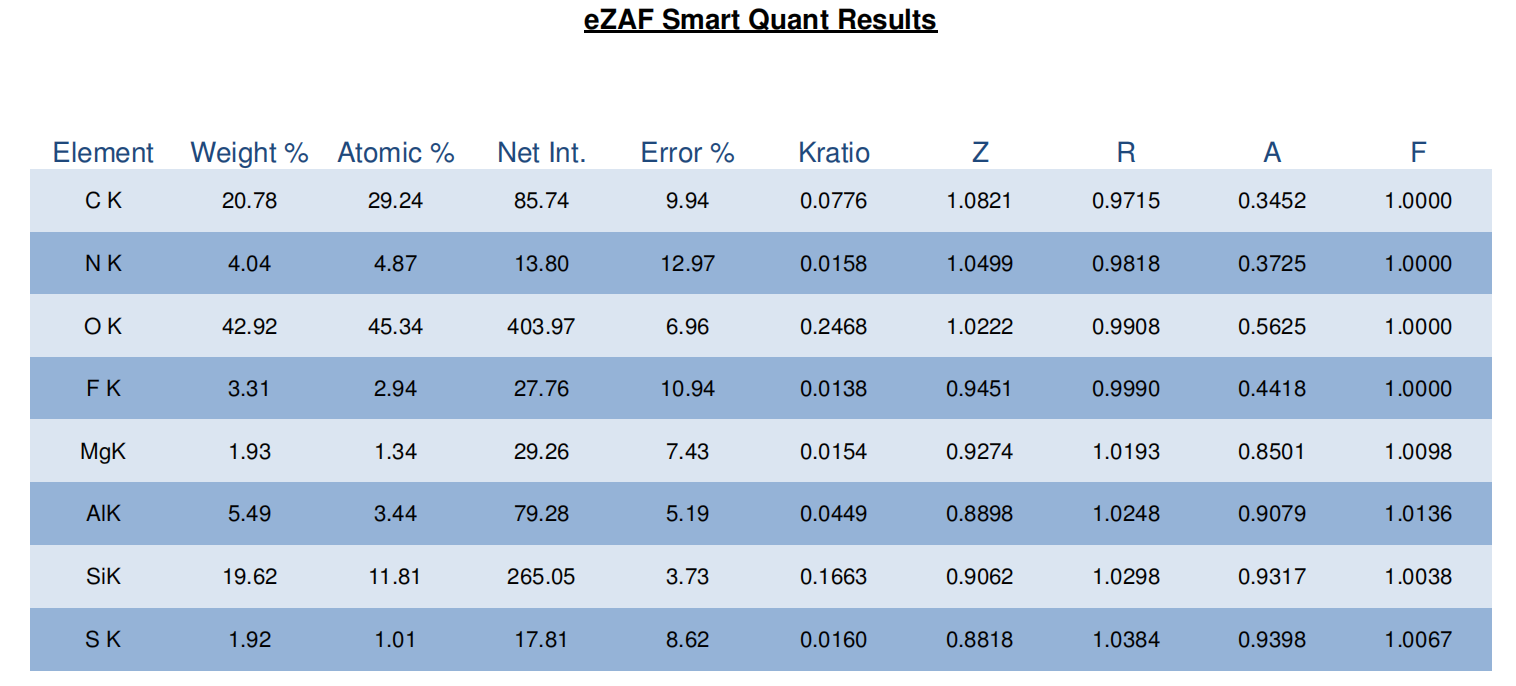


Sample No.6.


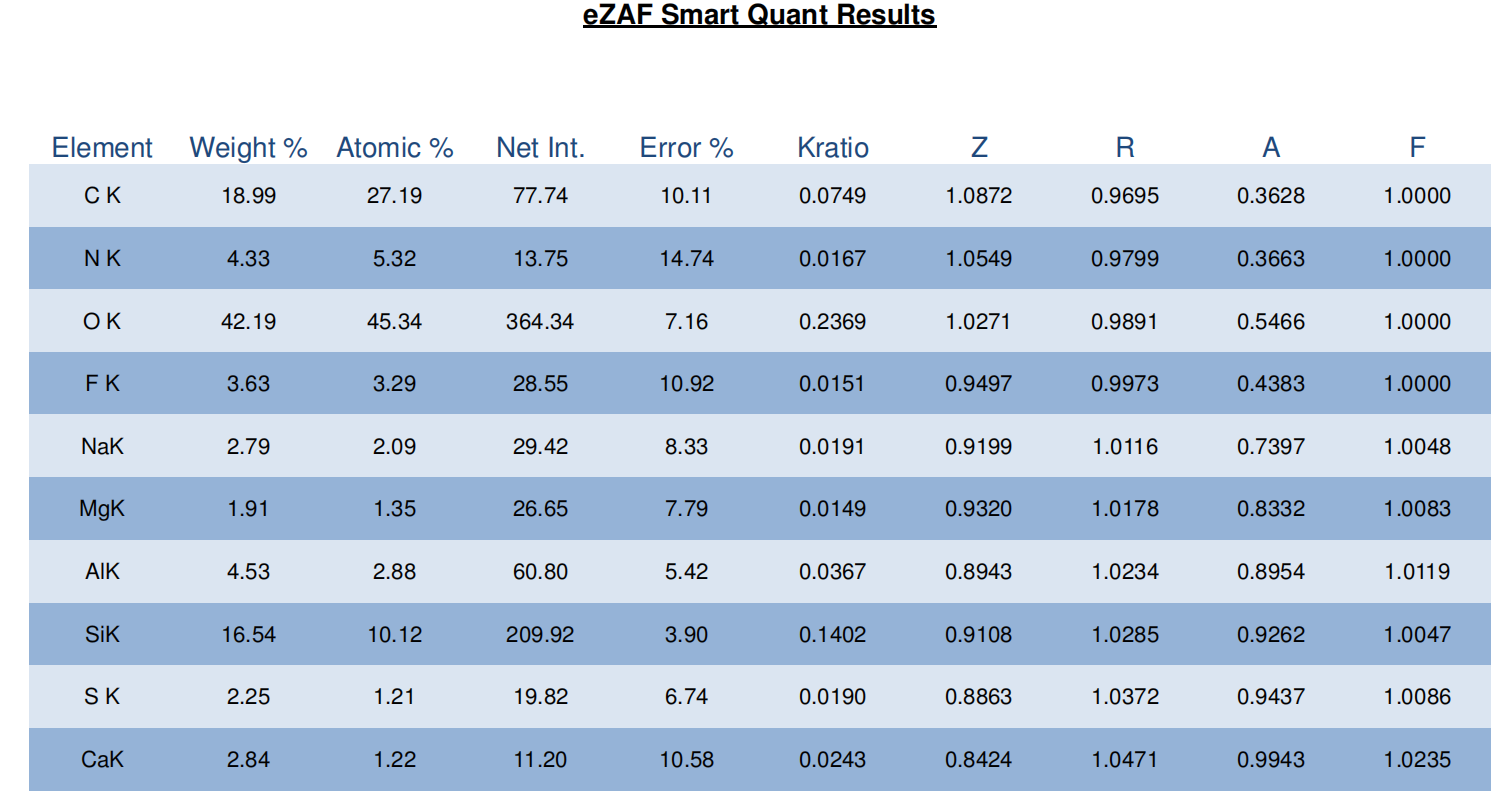


Sample No.7.


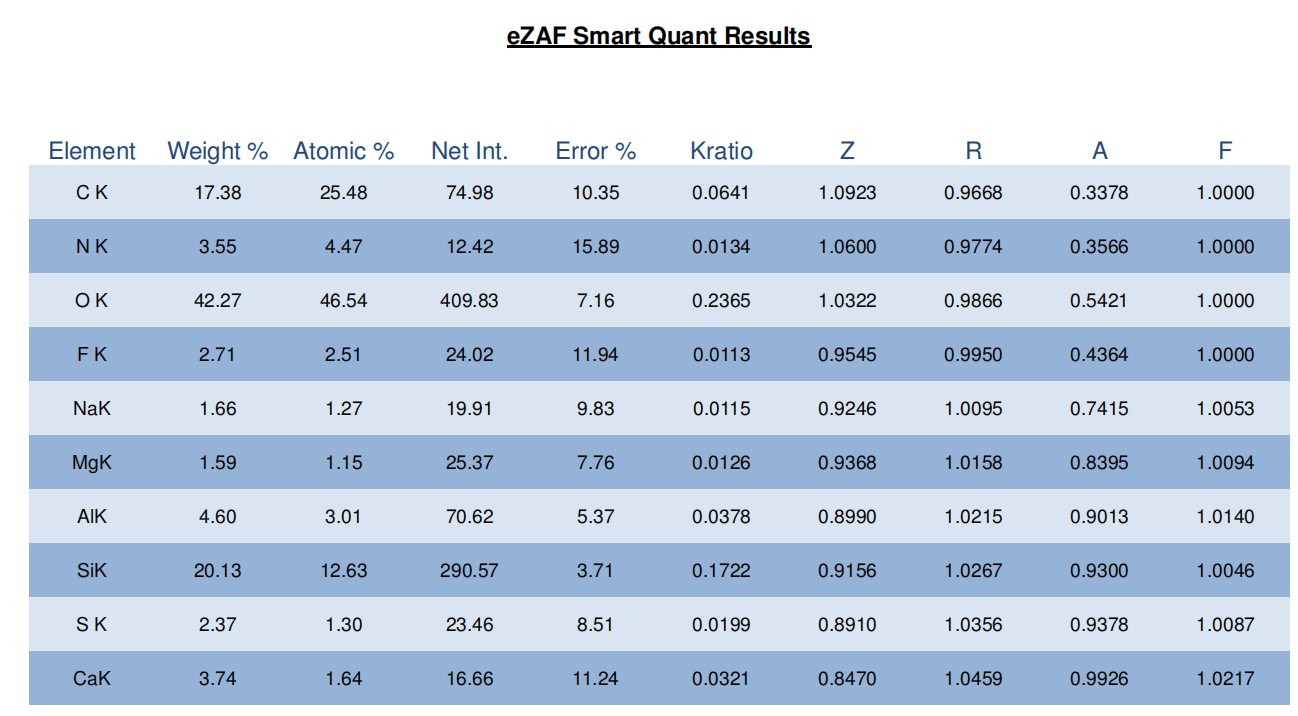


Sample No.8.


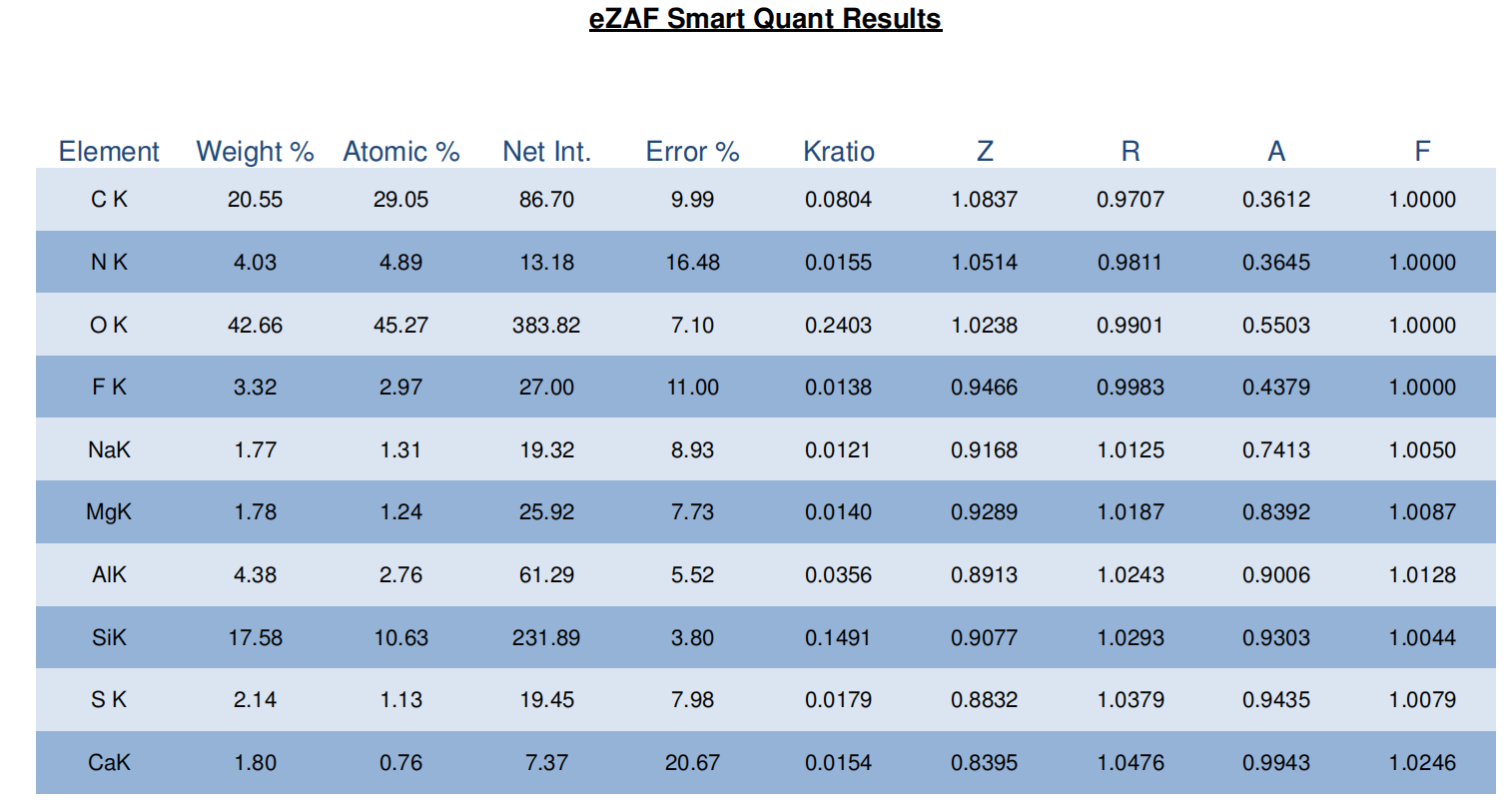


Sample No.9.


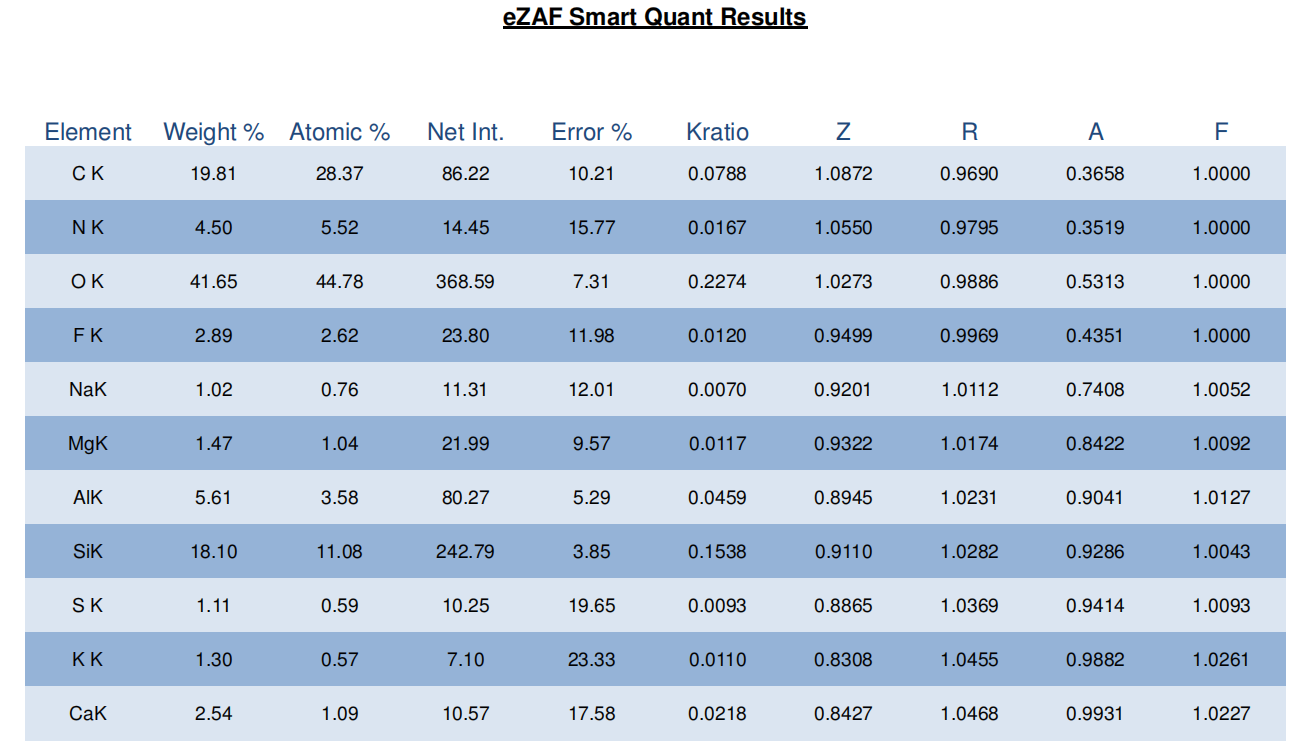


Sample No.10.


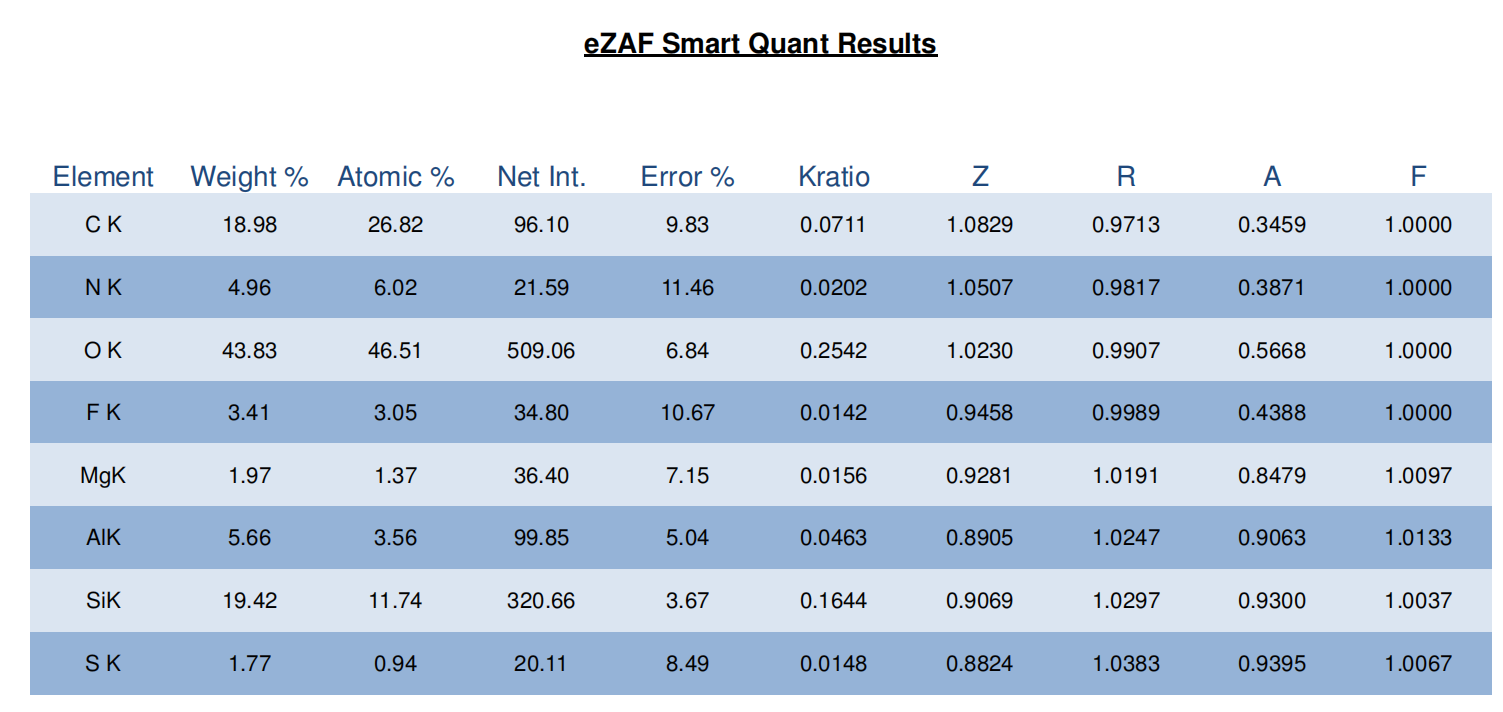


Sample No.11.


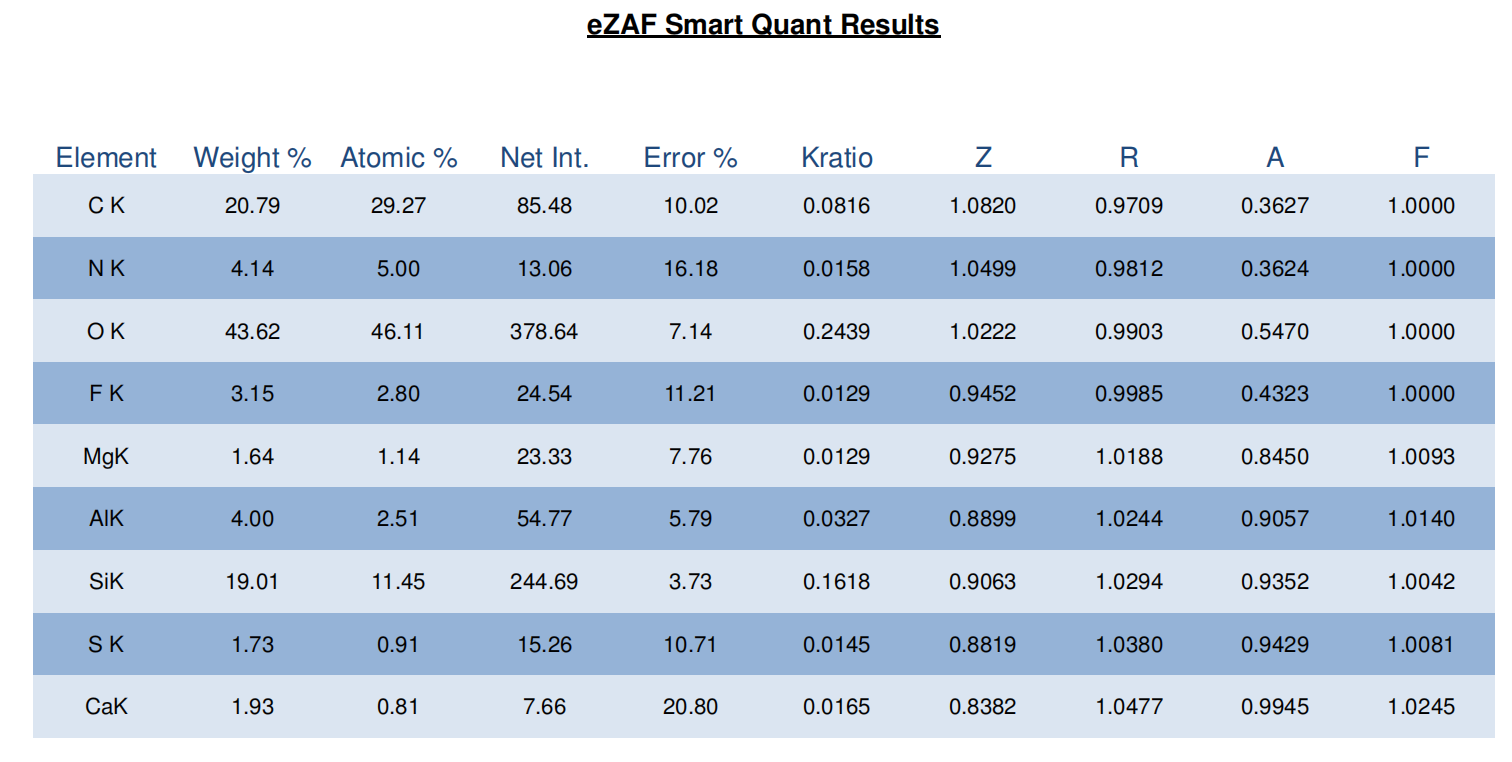


Sample No.12.


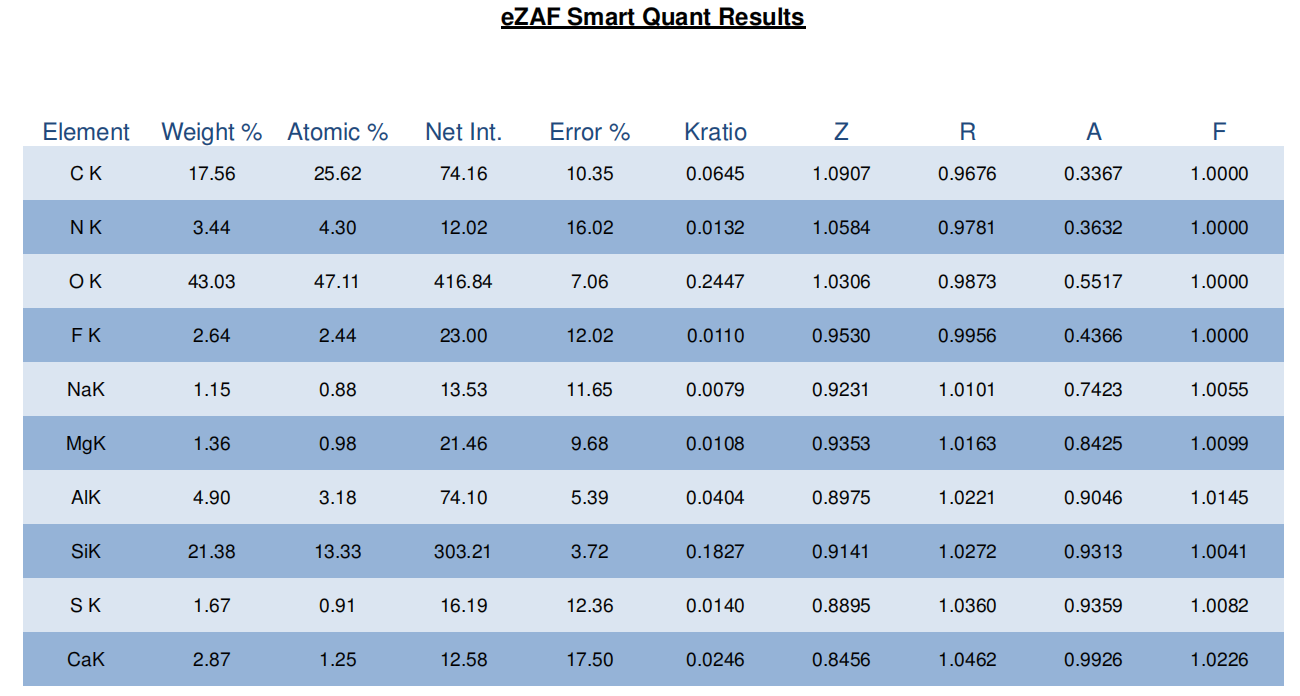


Sample No.13.


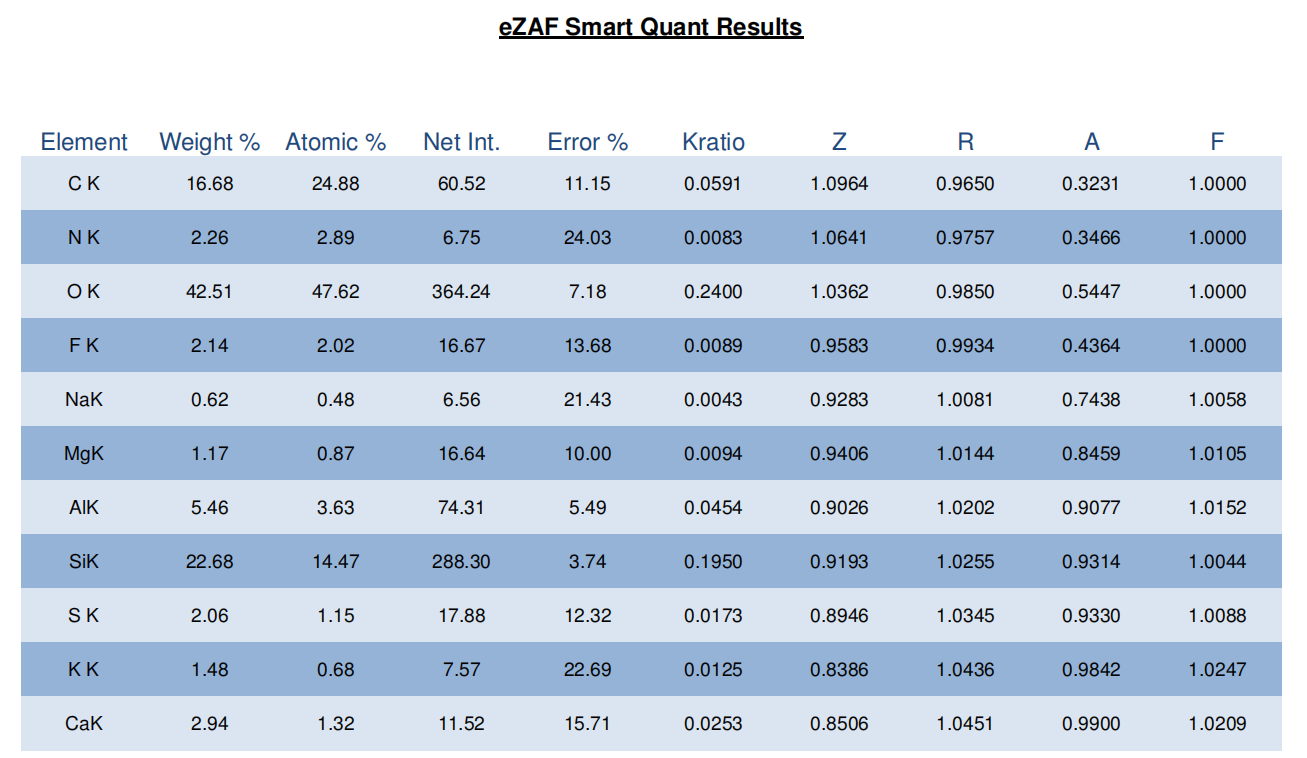


Sample No.14.


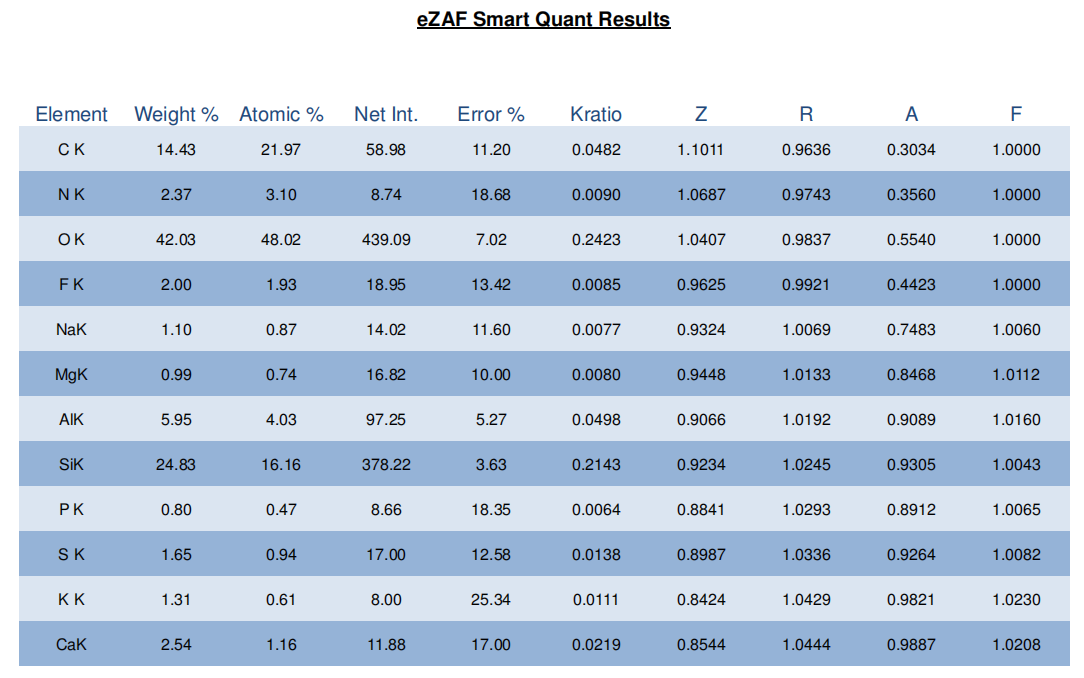


Sample No.15.


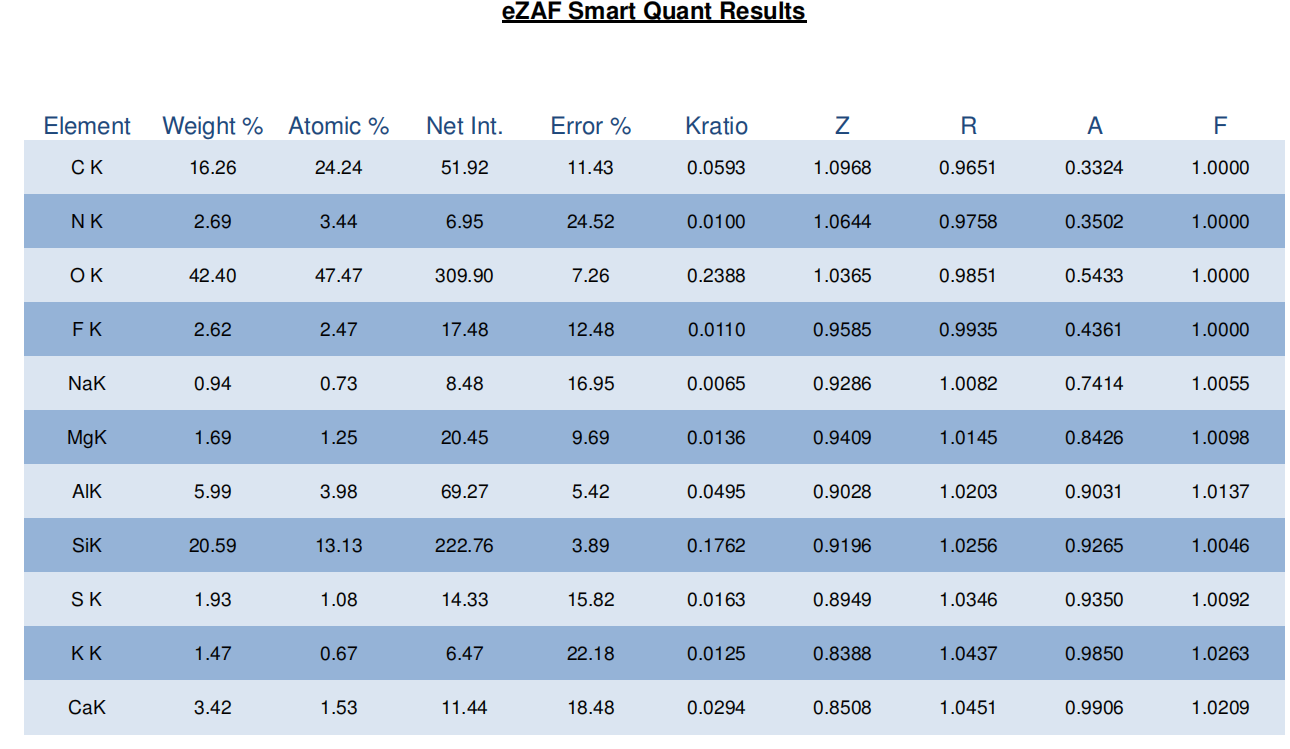


Sample No.16.


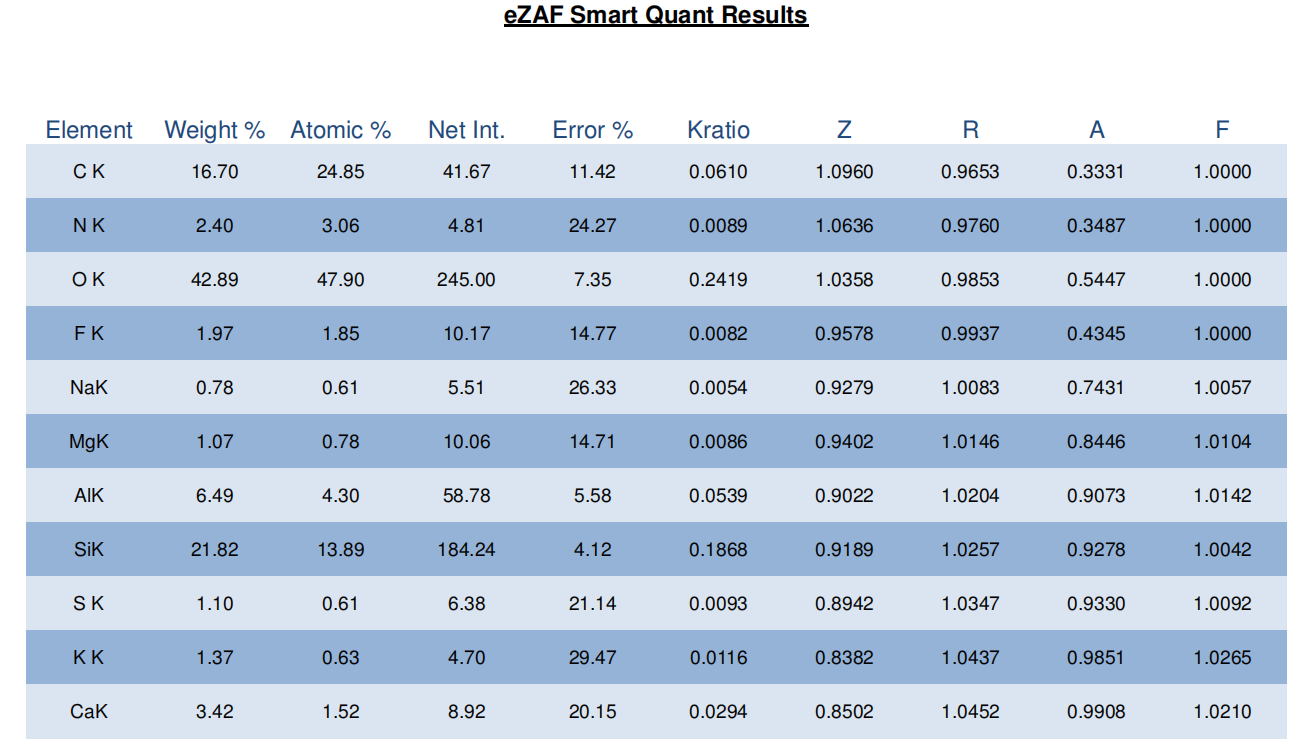


Sample No.17.


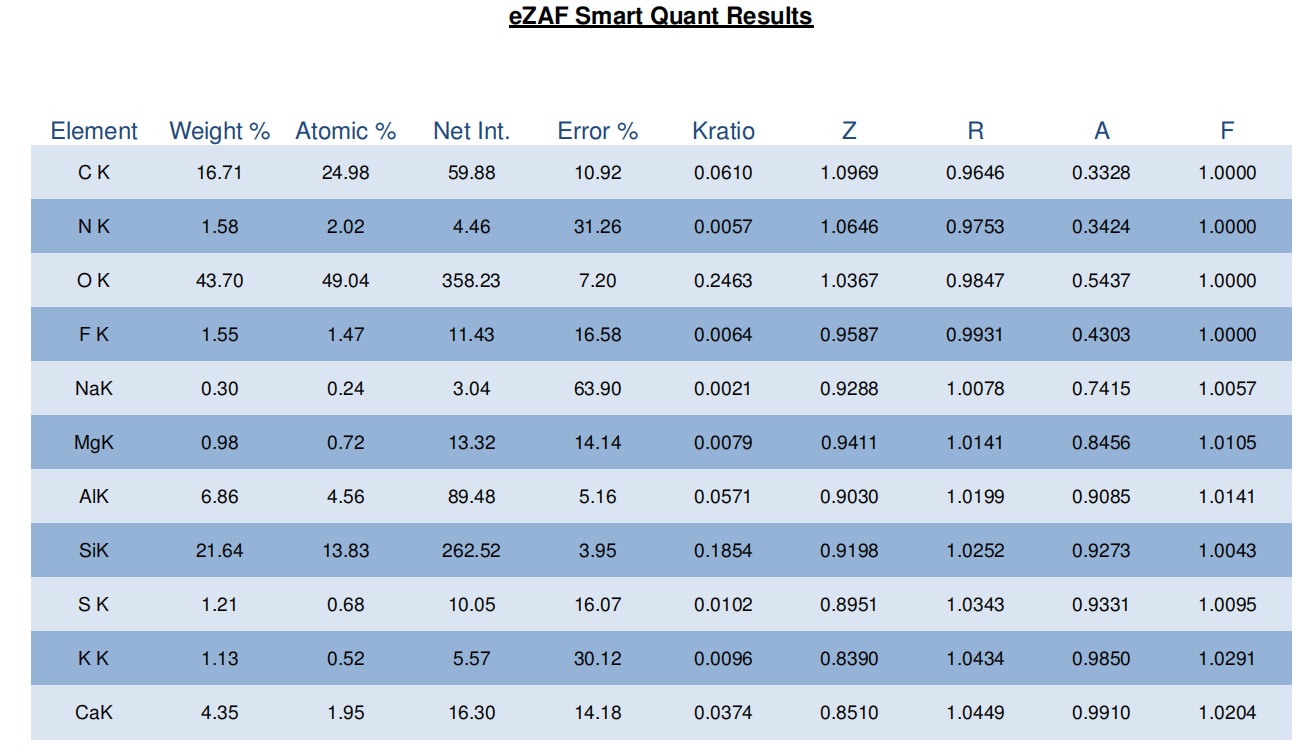


Sample No.18.


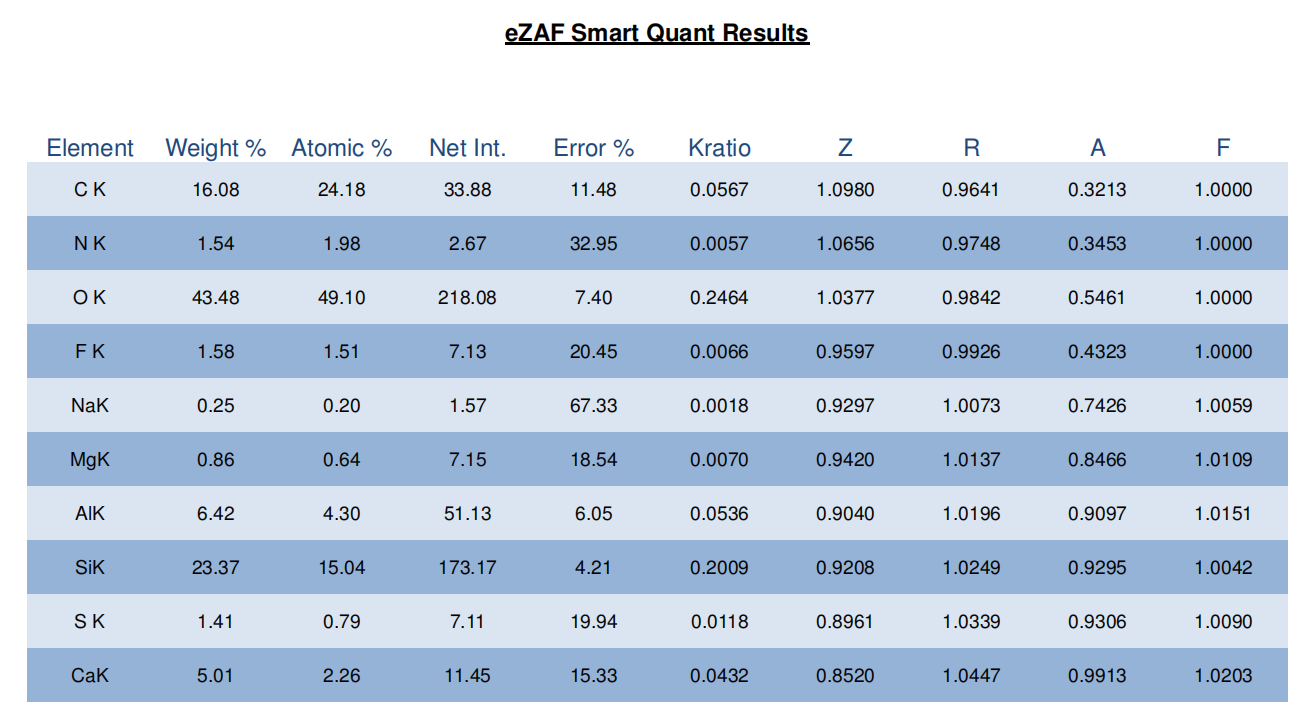


Sample No.19.


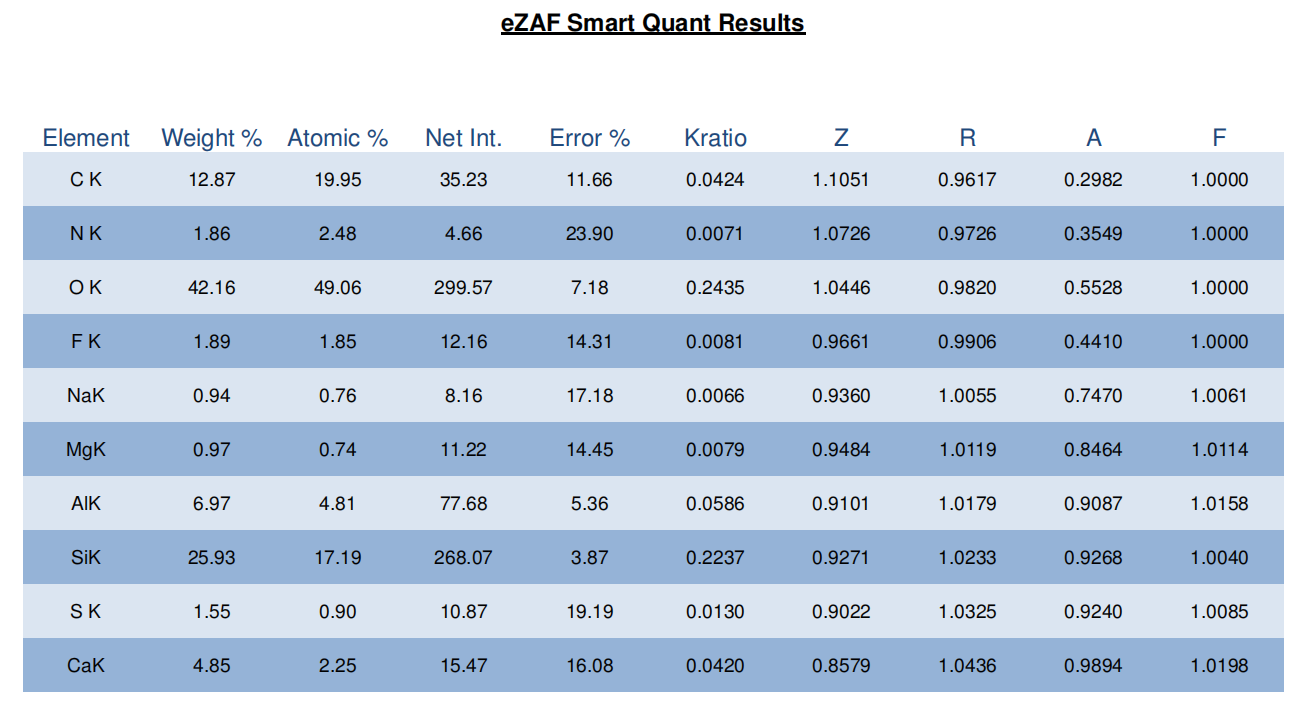


Sample No.20.


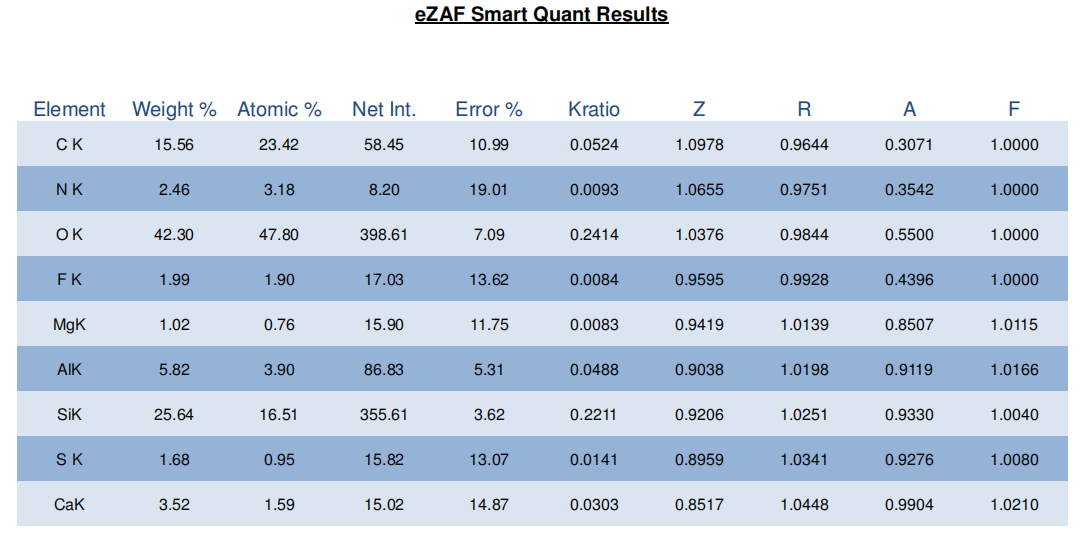


**FTIR Test Results**

Sample No.1.


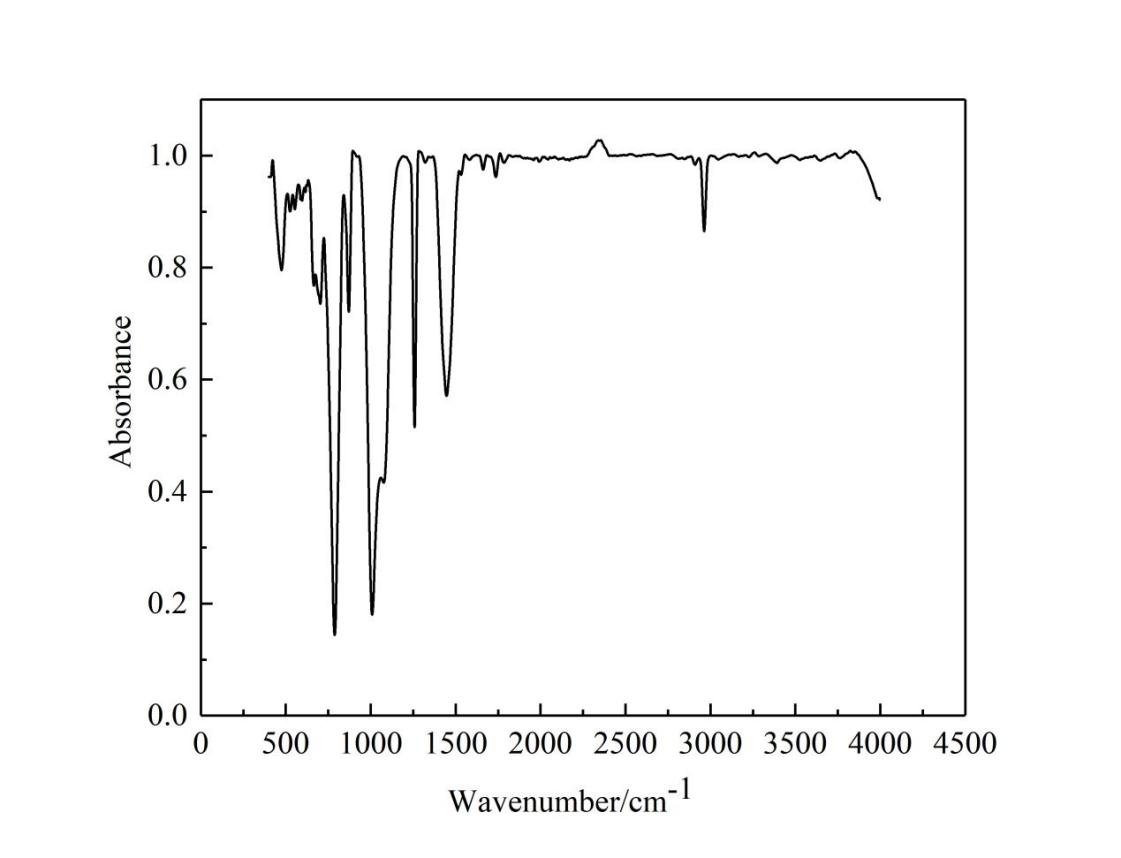


Sample No.2.


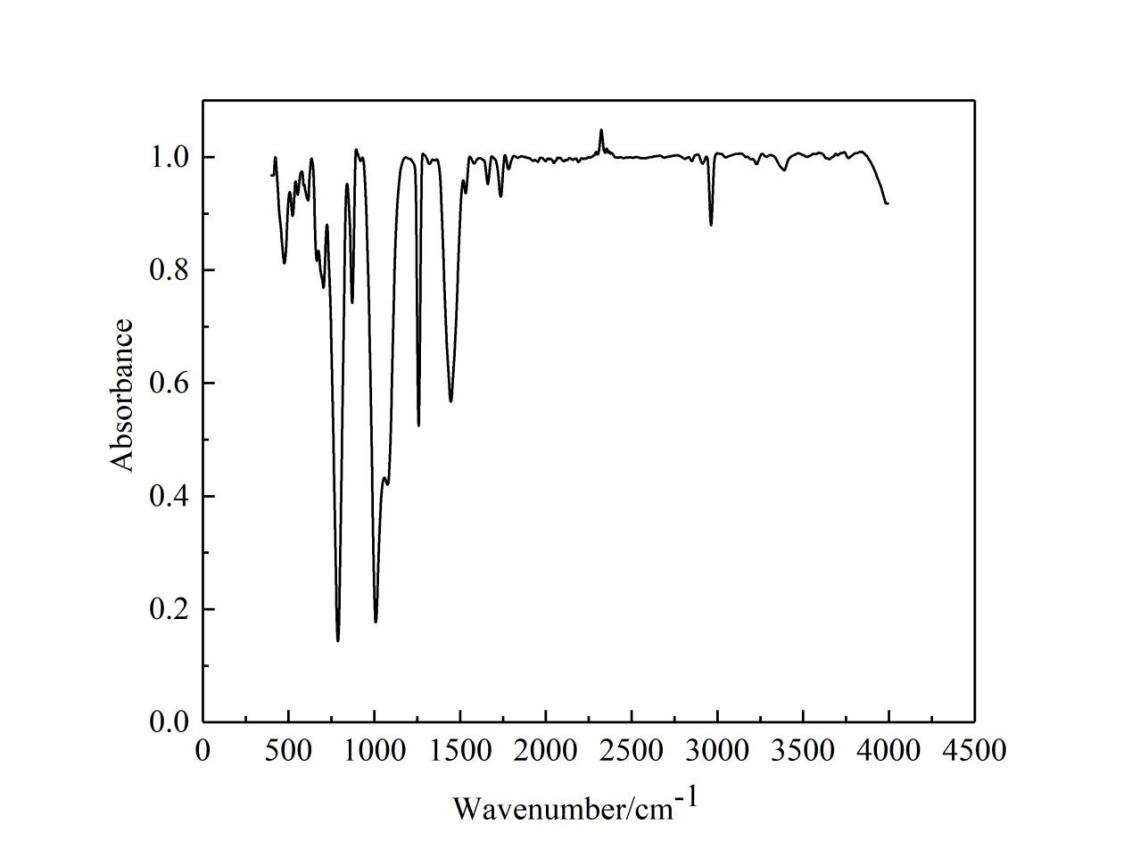


Sample No.3.


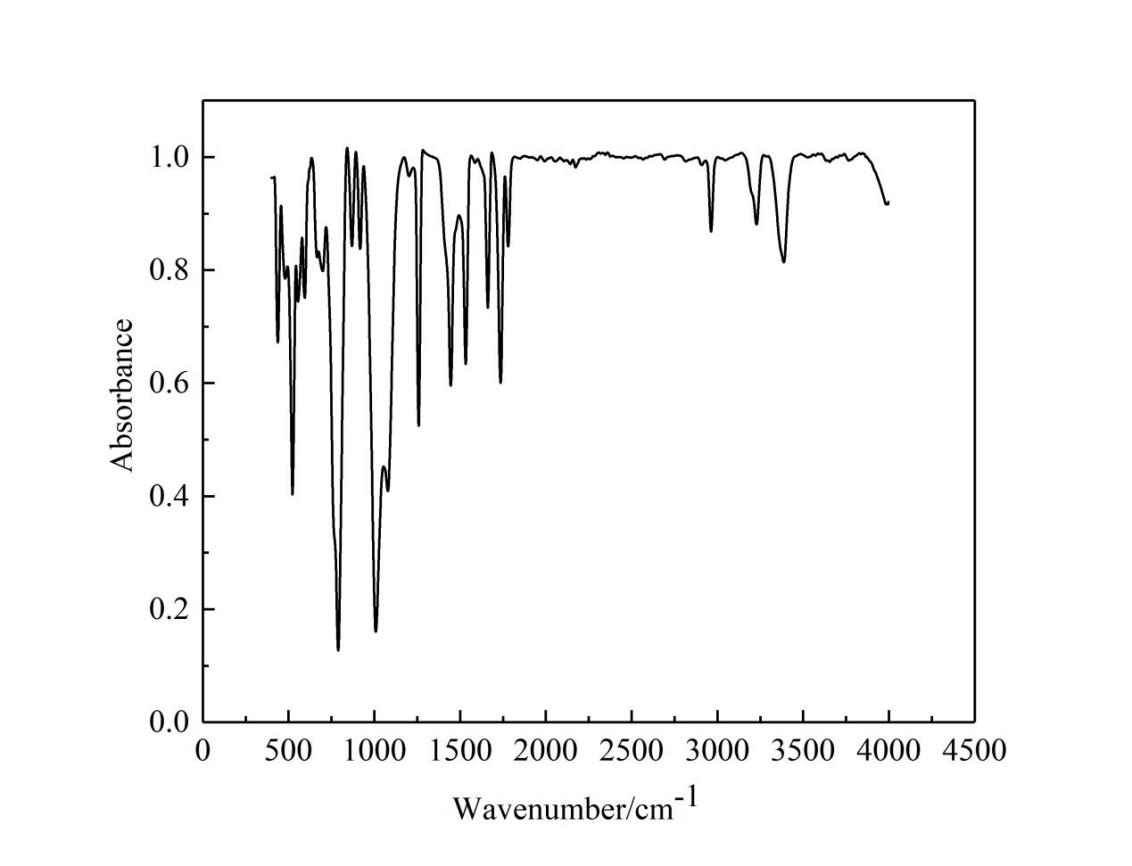


Sample No.4.


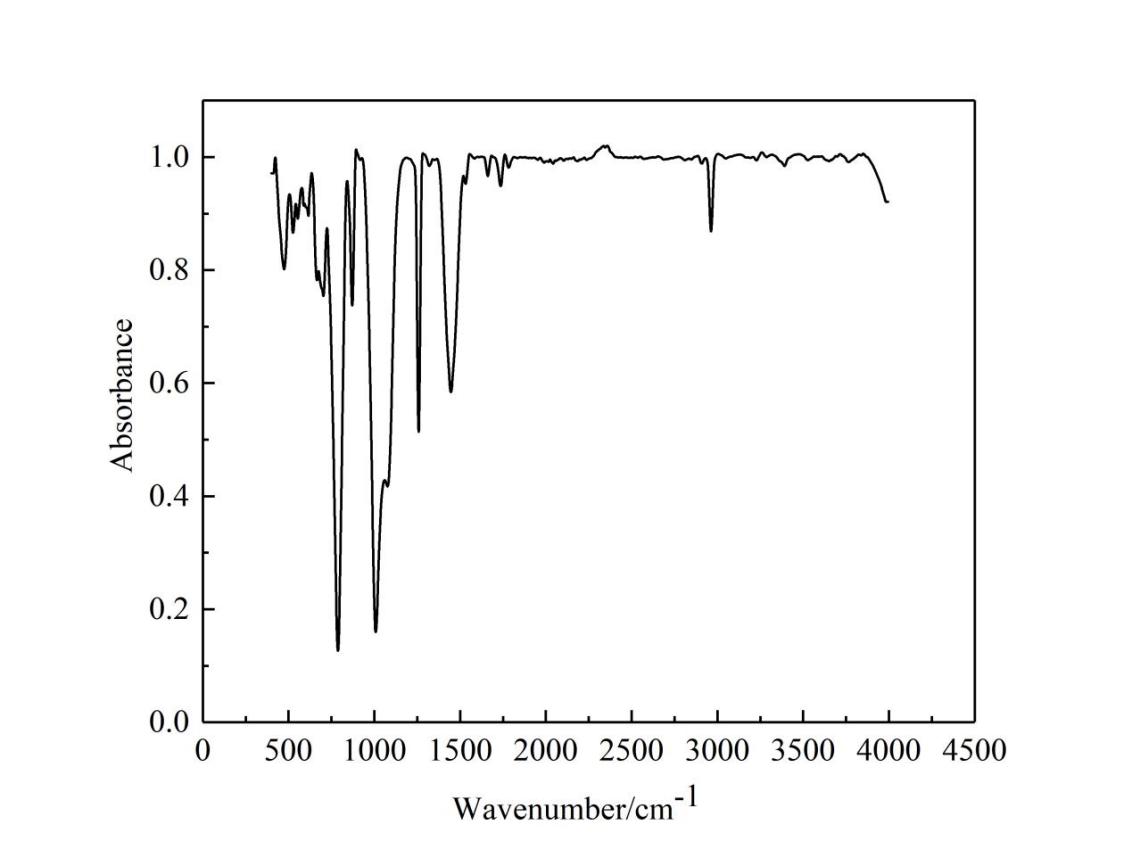


Sample No.5.


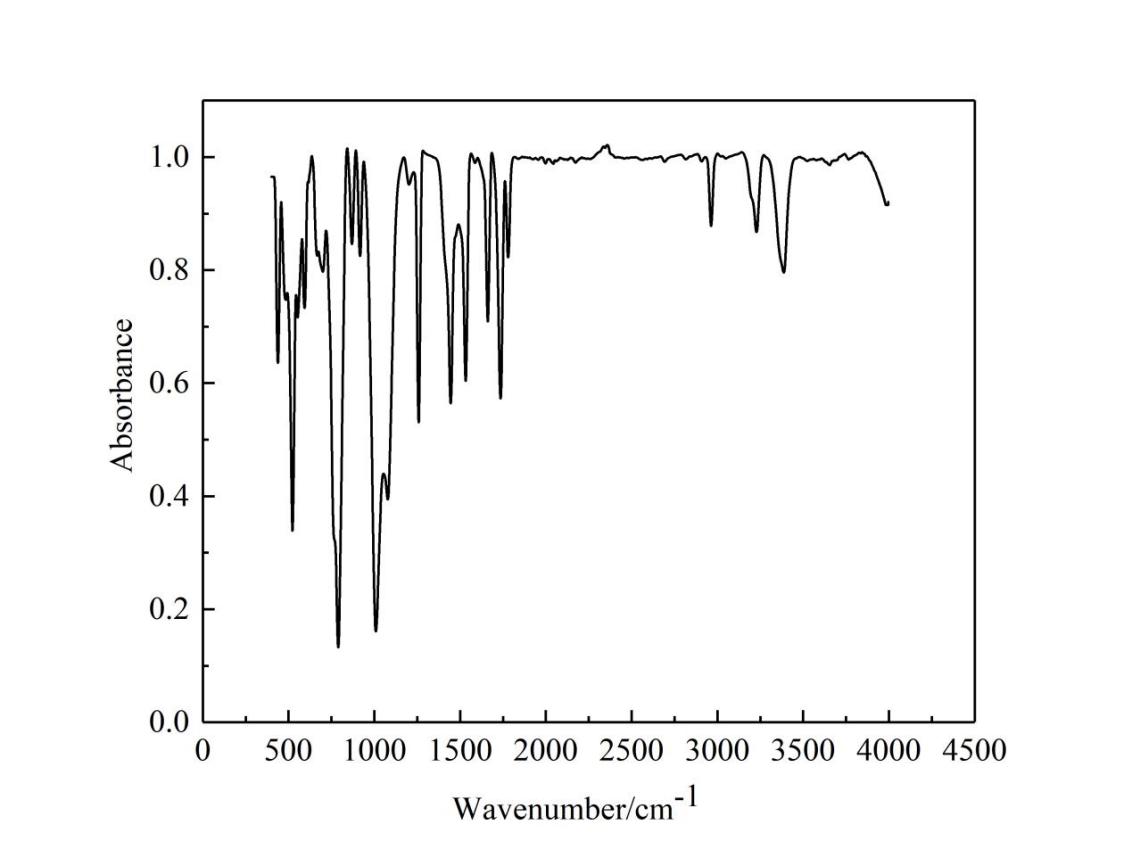


Sample No.6.


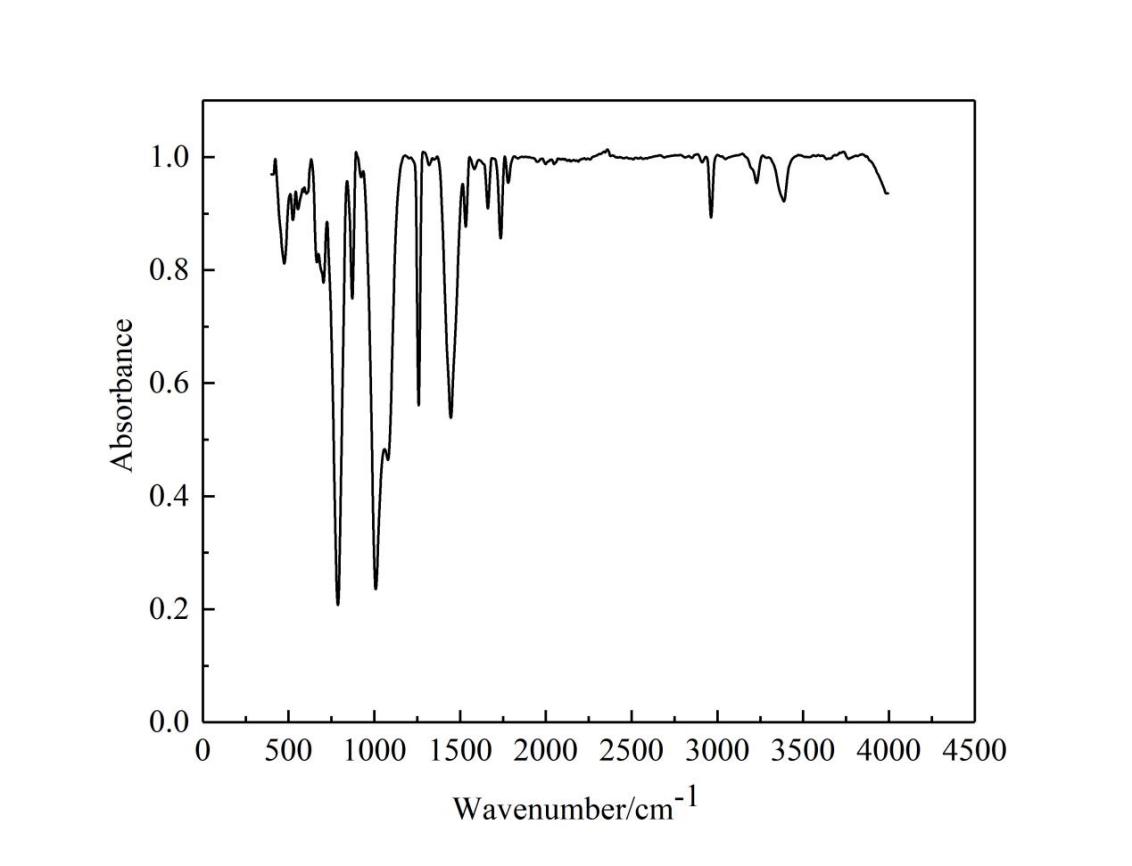


Sample No.7.


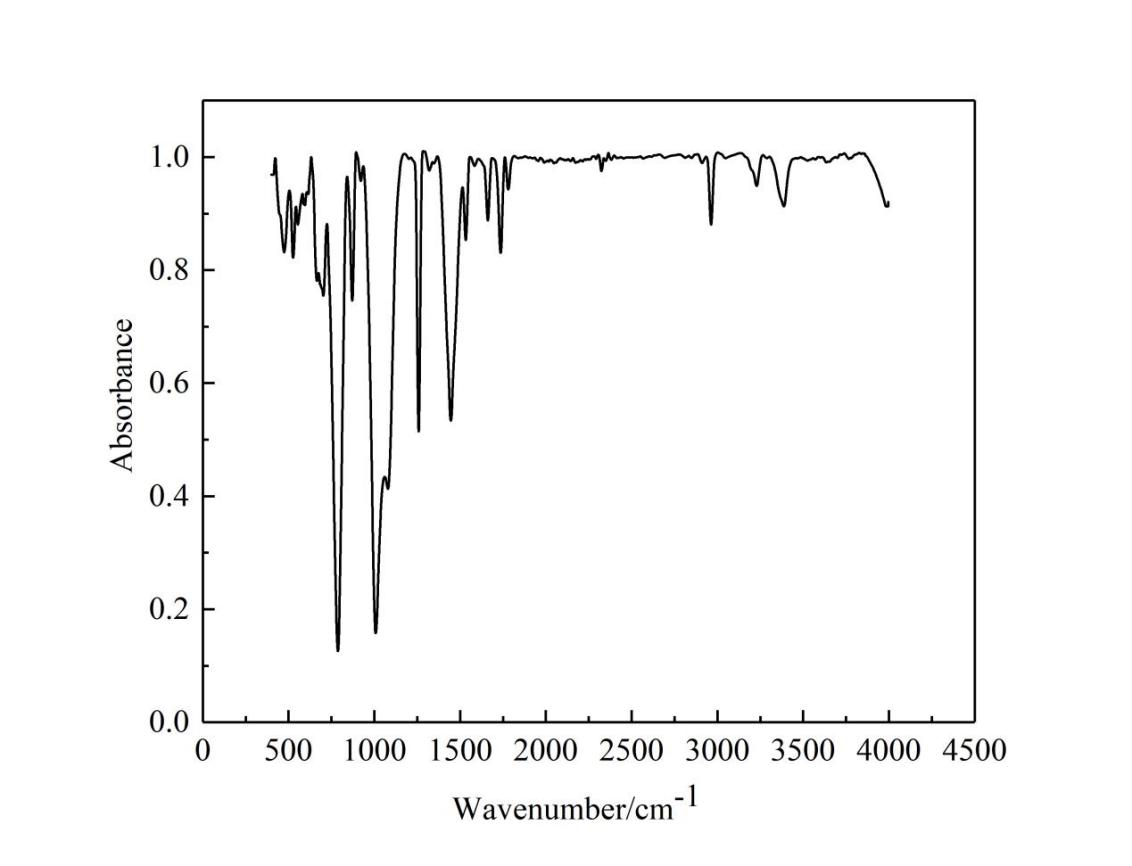


Sample No.8.


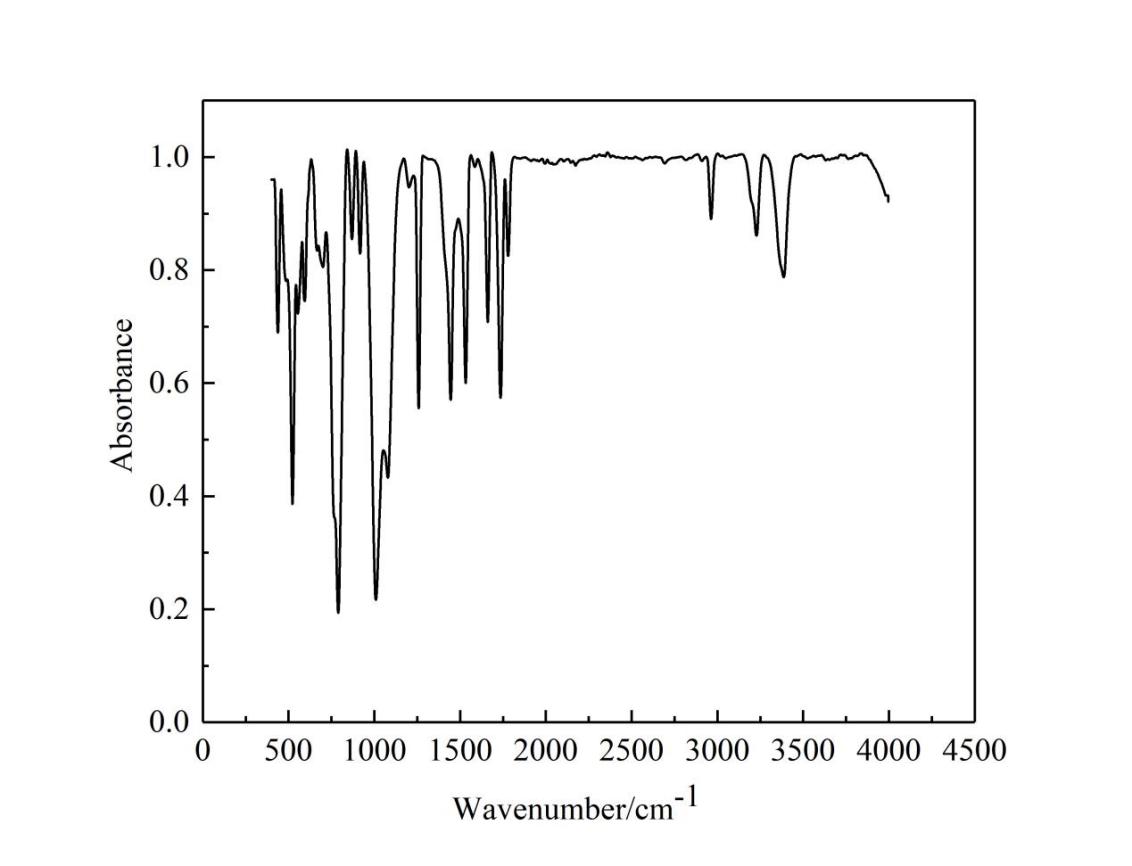


Sample No.9.


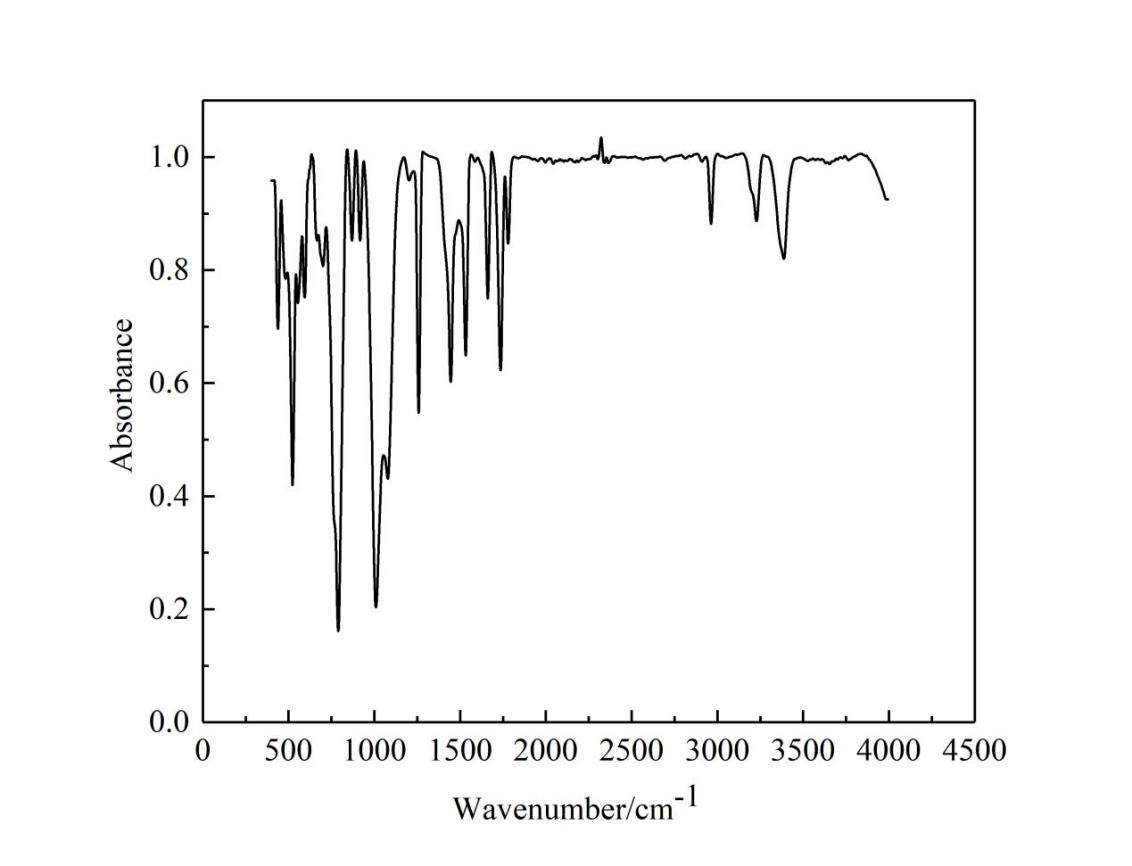


Sample No.10.


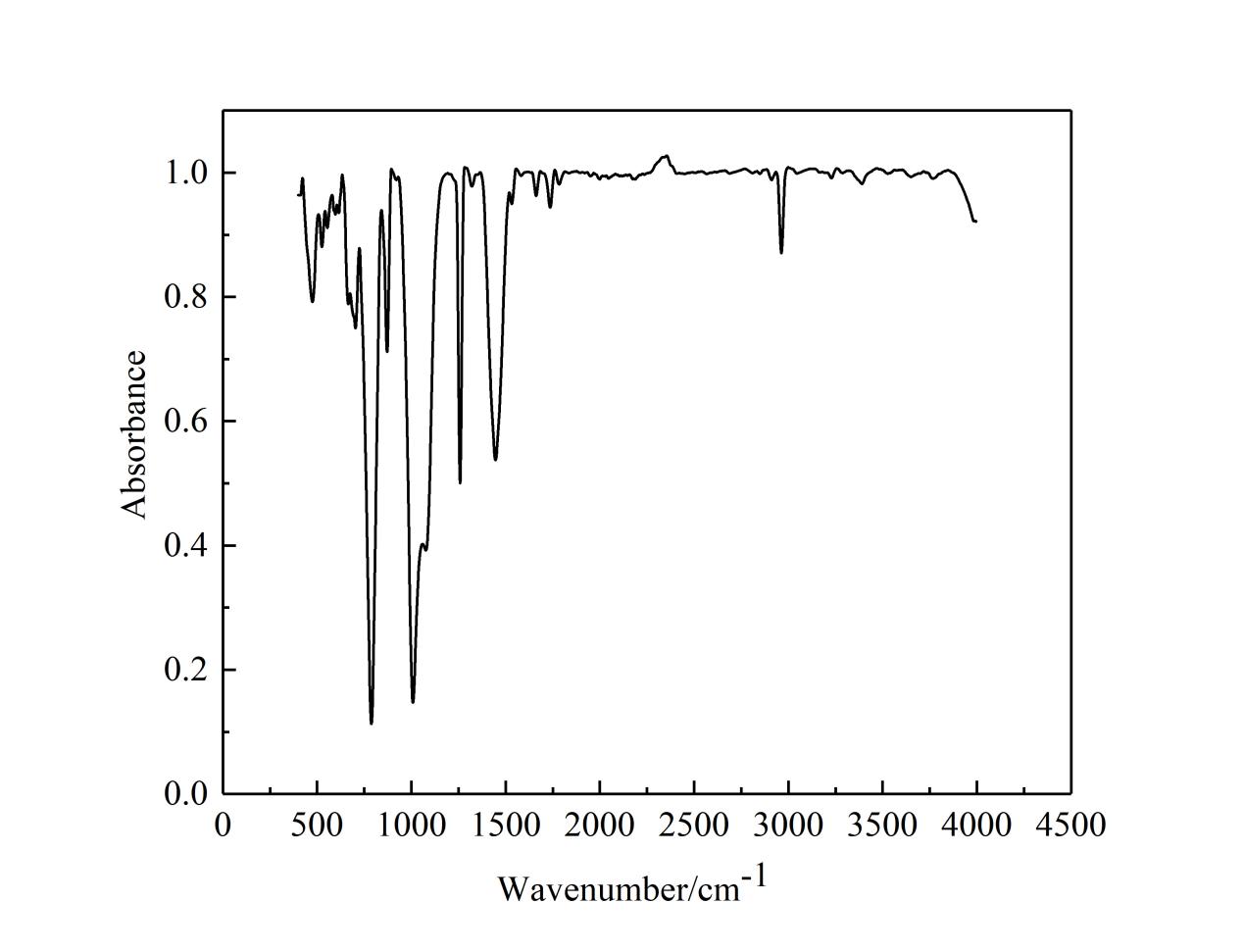


Sample No.11.


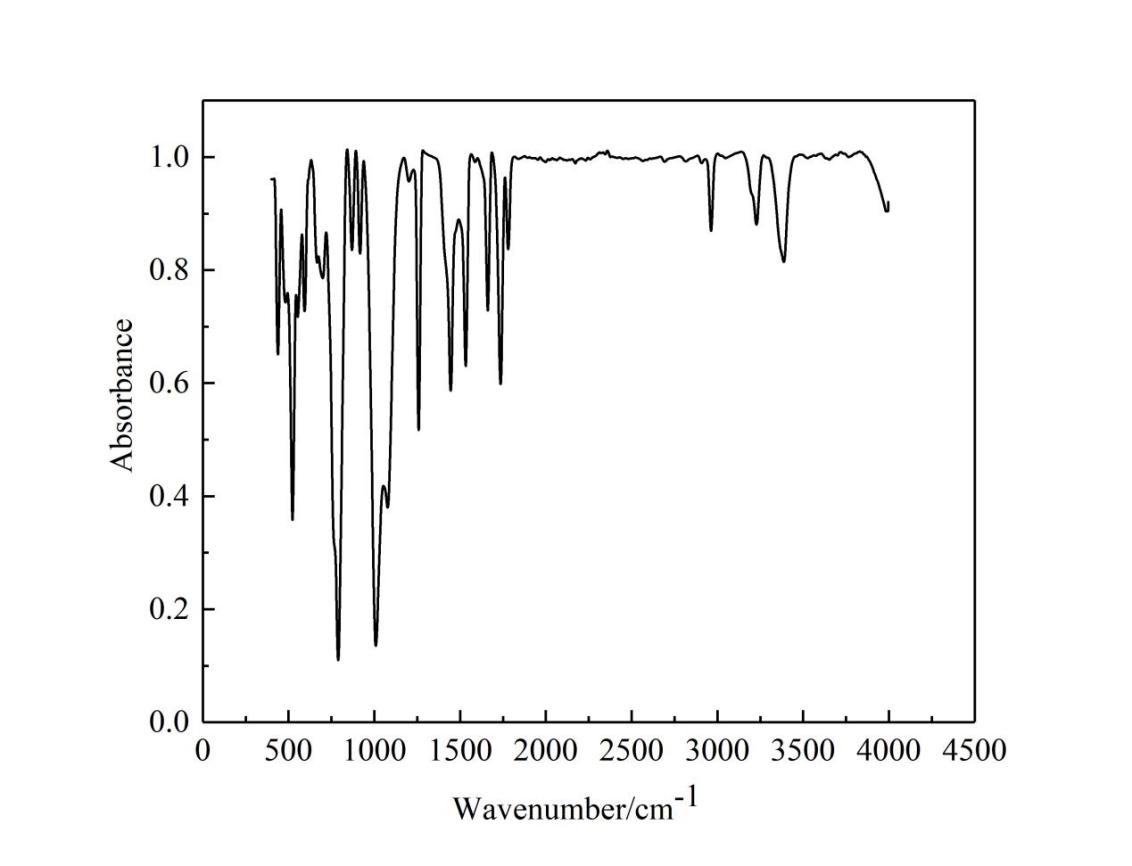


Sample No.12.


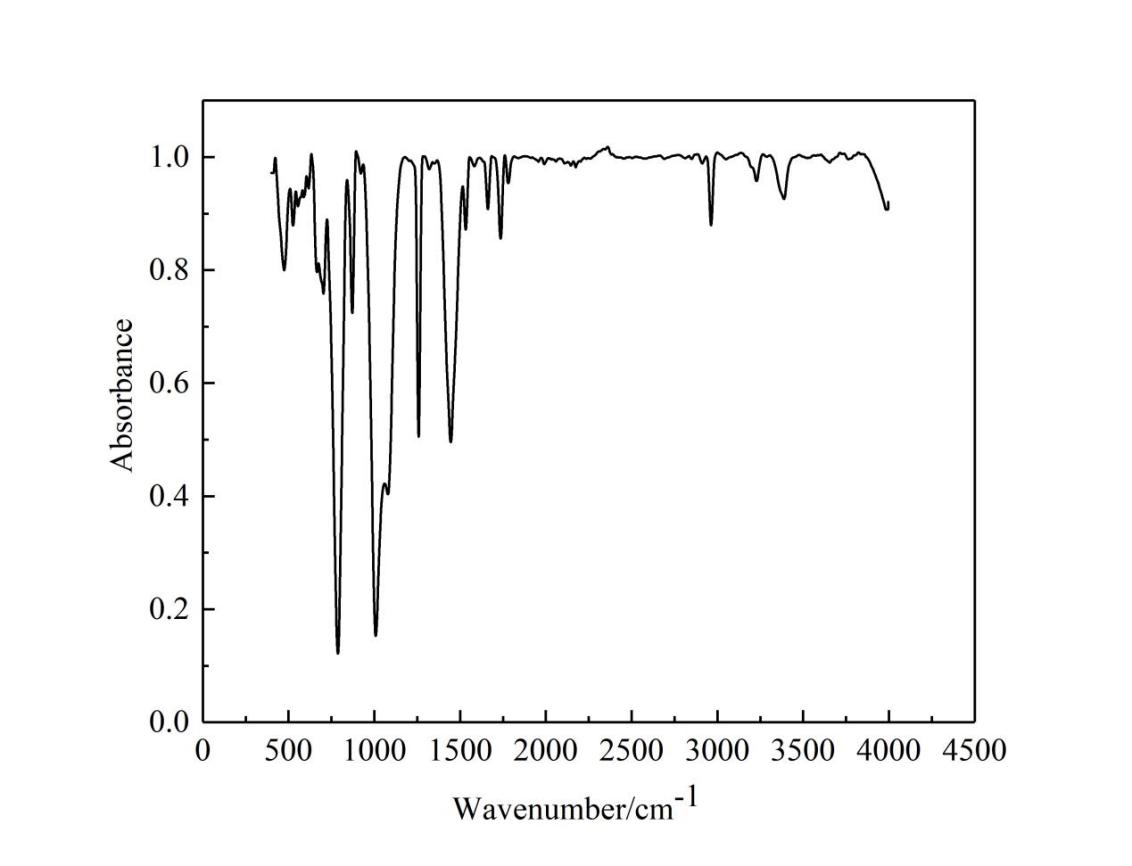


Sample No.13.


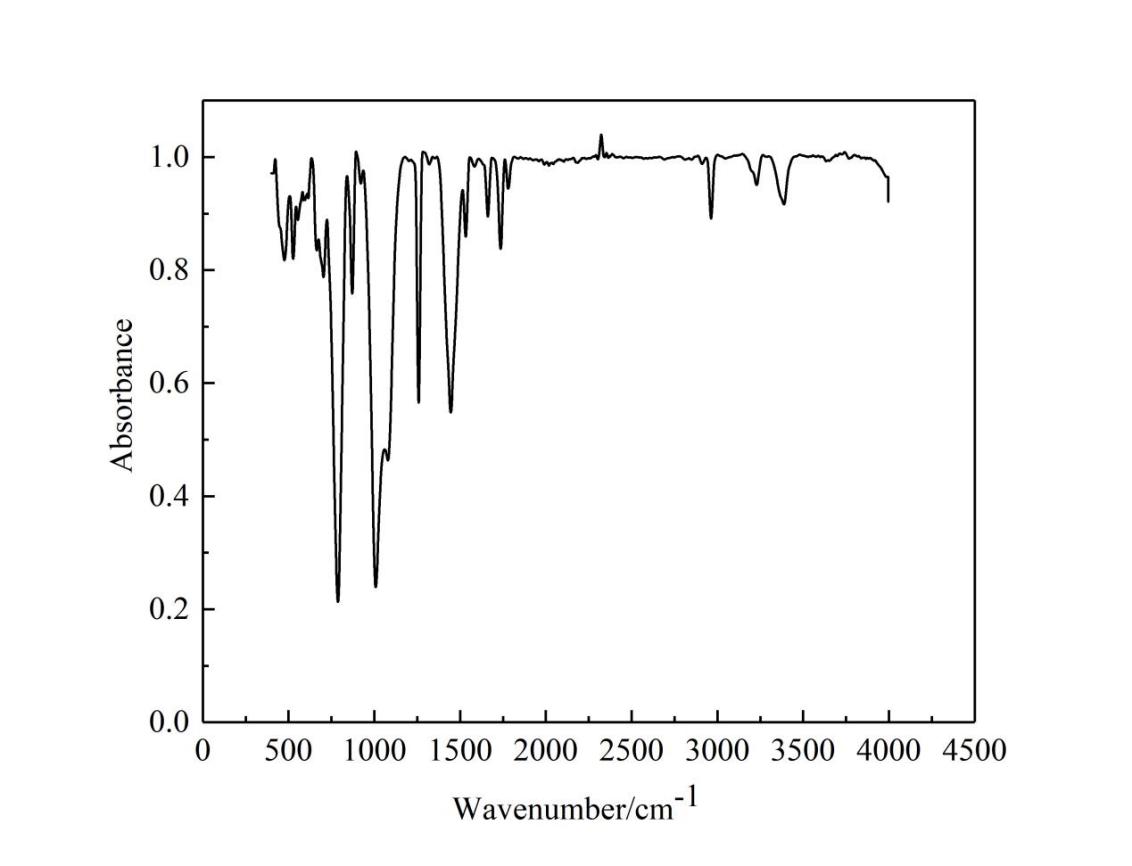


Sample No.14.


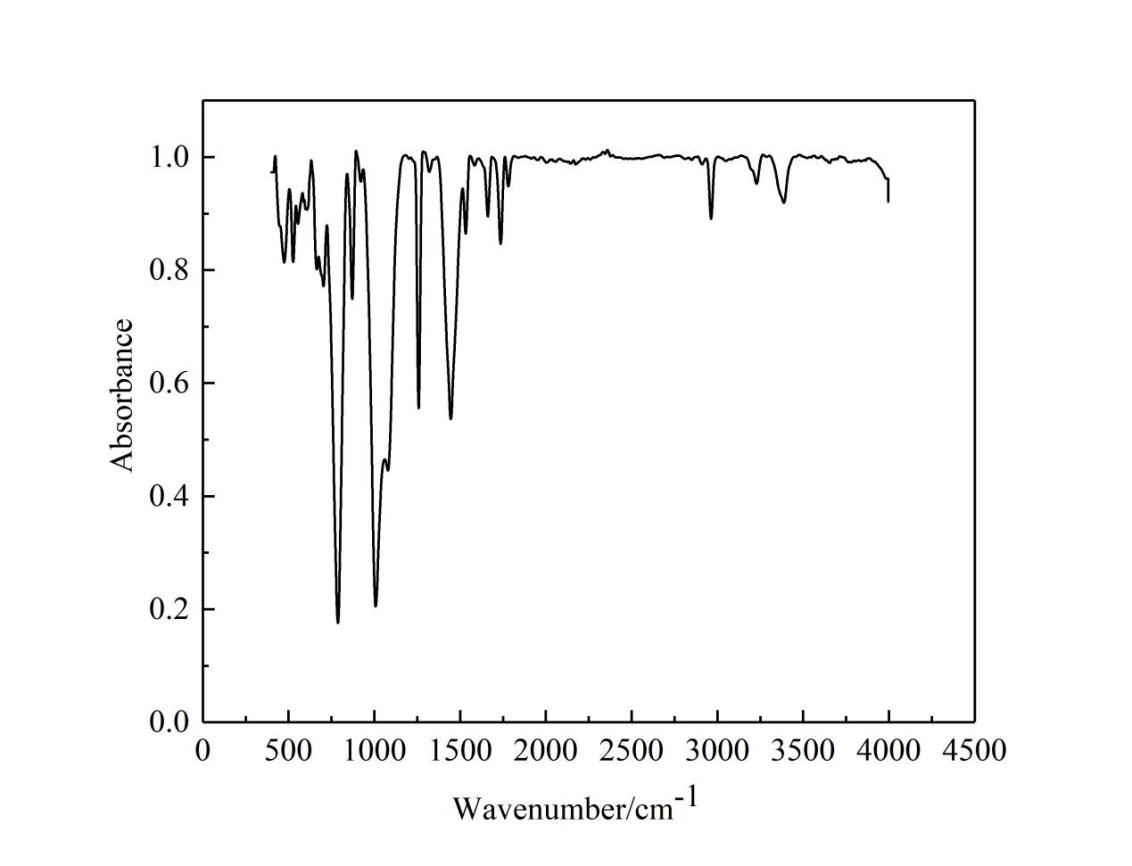


Sample No.15.


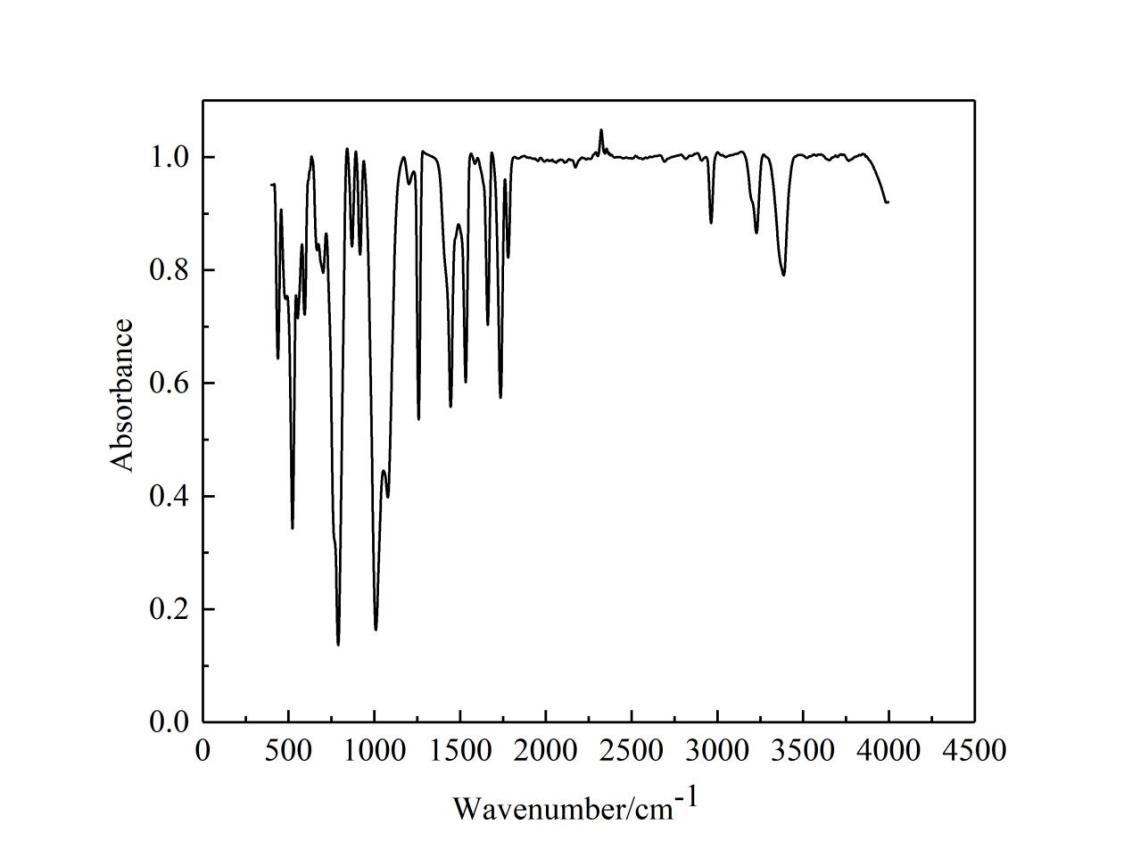


Sample No.16.


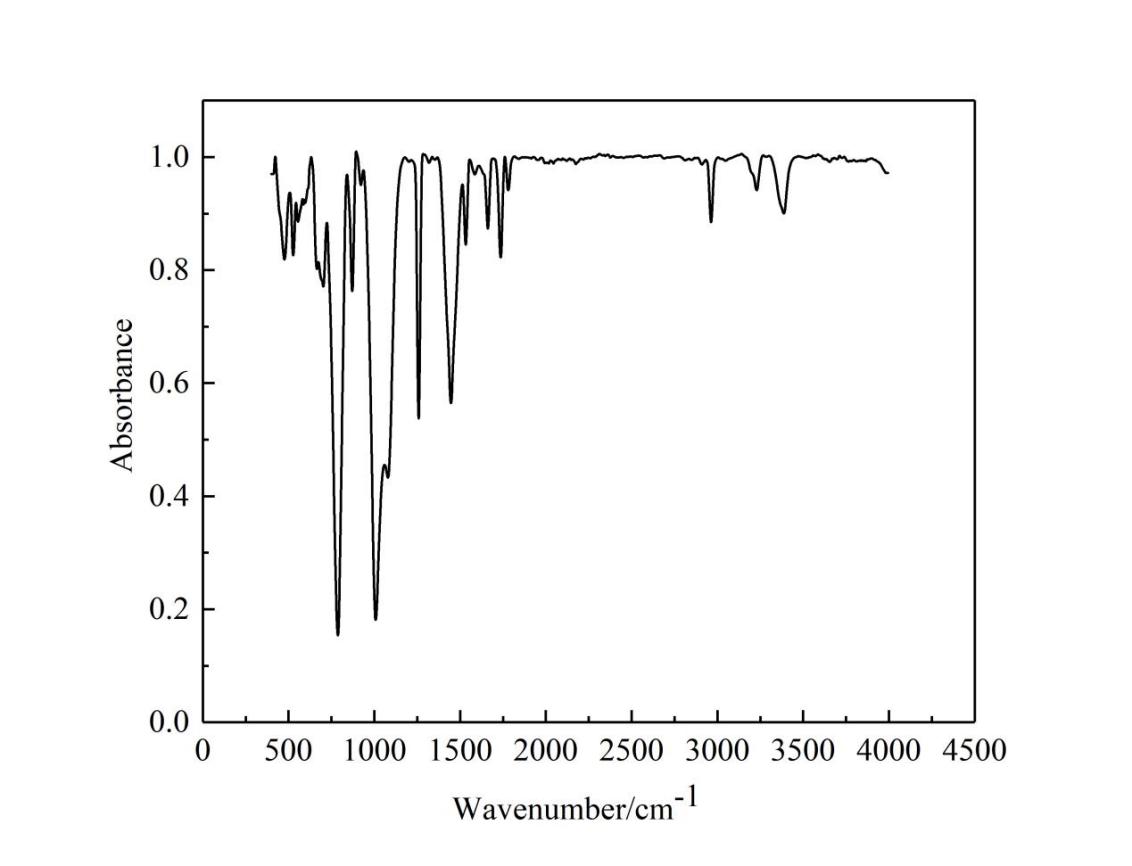


Sample No.17.


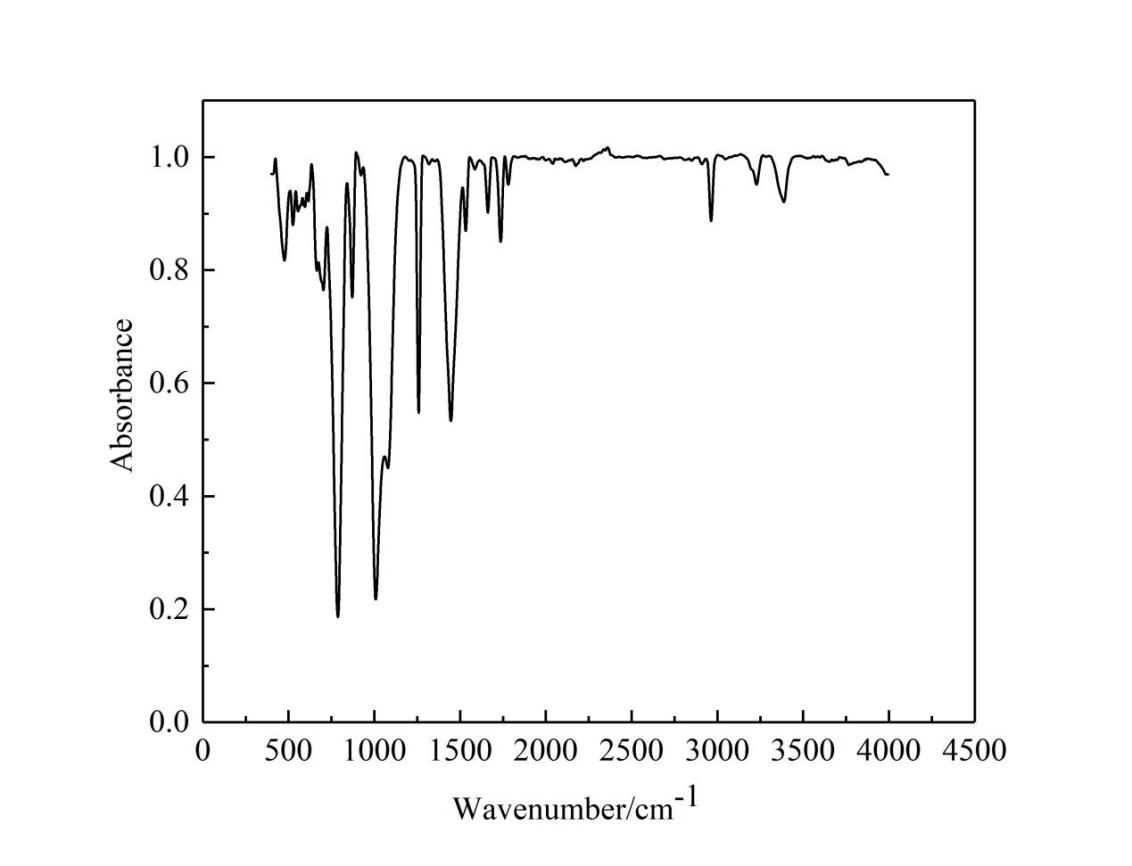


Sample No.18.


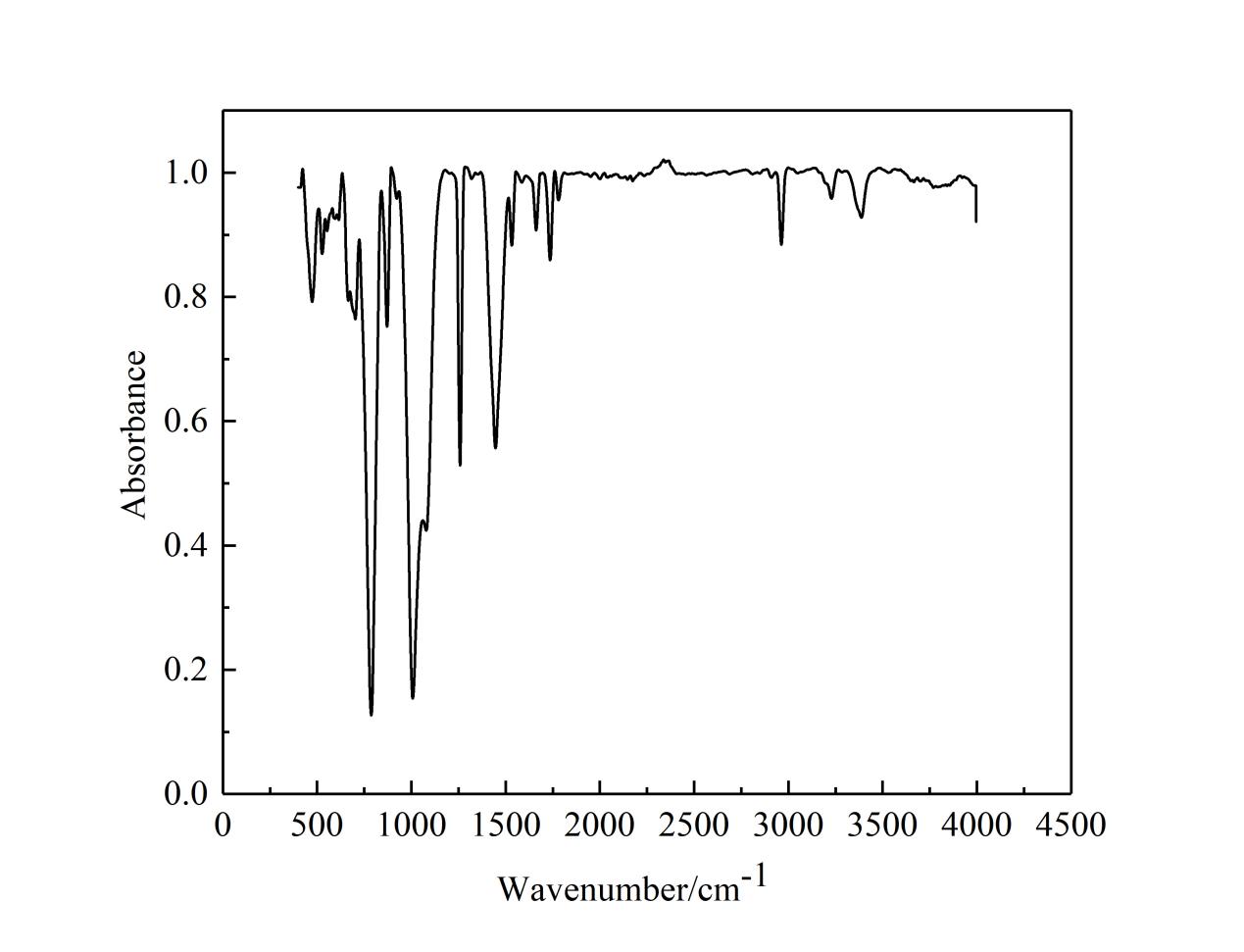


Sample No.19.


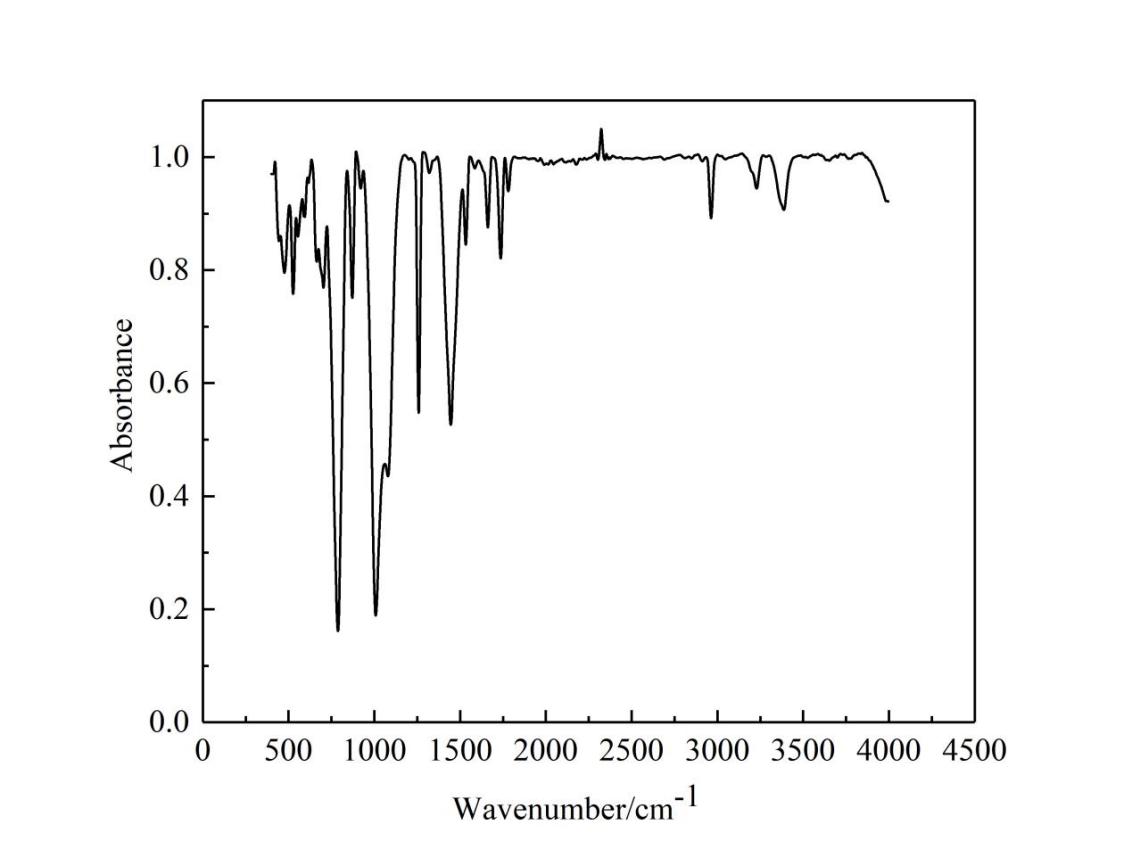


Sample No.20.


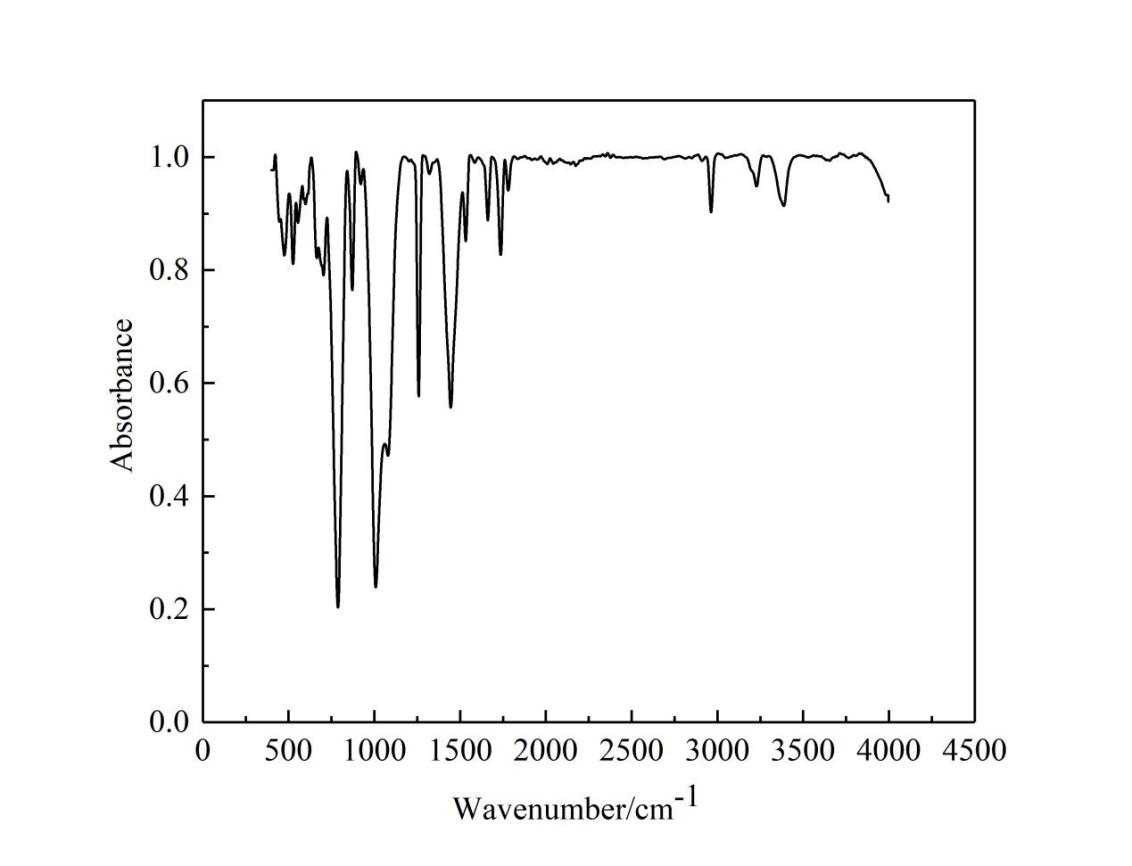

Supplement: S1 Dataset — (DOCX) [file pone.0251092.s001.docx]
